# Supplementary material for: Charge-altering releasable transporters enhance mRNA delivery in vitro and exhibit in vivo tropism
Source: Nat Commun. 2023 Nov 1;14:6983. doi: 10.1038/s41467-023-42672-x (PMC10620205; doi:10.1038/s41467-023-42672-x)
Supplement: Supplementary file 1 — Supplementary Information [file 41467_2023_42672_MOESM1_ESM.pdf]

Supplementary Information

Charge-altering releasable transporters enhance mRNA delivery in vitro and exhibit in vivo tropism

Zhijian Li[1 †], Laura Amaya[2,3 †], Ruoxi Pi[4], Sean K. Wang[2,5], Alok Ranjan[1], Robert M. Waymouth[1], Catherine A. Blish[4,6], Howard Y. Chang[2,7], Paul A. Wender[1,8,*]

[1]Department of Chemistry, Stanford University, Stanford, CA 94305, USA

[2] Center for Personal Dynamic Regulomes, Stanford University, Stanford, CA 94305, USA

[3] Institute for Stem Cell Biology and Regenerative Medicine, Stanford University School of Medicine, Stanford, CA 94305, USA

[4] Division of Infectious Diseases and Geographic Medicine, Department of Medicine, Stanford, CA 94305, USA

[5] Department of Ophthalmology, Stanford University School of Medicine, Stanford, CA 94305, USA

[6] Chan Zuckerberg Biohub, San Francisco, CA

[7]Howard Hughes Medical Institute, Stanford University, Stanford, CA 94305, USA

[8]Department of Chemical and Systems Biology, Stanford University, Stanford, CA 94305, USA

TABLE OF CONTENTS

Supplementary Tables	3
Supplementary Figures	4
NMR spectra	20
Supplementary Methods	46
General procedures	46
Reagent list (commercial source and abbreviations)	47
Synthetic procedures and chemical characterization data	48
Supplementary References	68

Supplementary Tables

Supplementary Table 1. Polymerization conditions of CARTs.

Abbreviation	BnOH (1 equiv[a])	Lipid block equiv[a]	Cationic block equiv[a]	PDI[b]	Yield%[c]
bAC-1a	1mg	17	10	1.315	54.1%
bAC-1b	1mg	10	11	1.367	72.4%
bAC-1c	1mg	10	18.5	1.223	65.7%
bAC-2a	1mg	17	10	1.256	77.3%
bAC-2b	1mg	10	11	1.145	65.7%
bAC-2c	1mg	10	18.5	1.252	77.1%
bAC-3a	0.5mg	17	10	1.182	70.2%
bAC-3b	1mg	10	11	1.247	60.3%
bAC-3c	1mg	10	18.5	1.173	71.6%
bAC-4a	1mg	17	10	1.174	58.4%
bAC-4b	1mg	10	11	1.24	81.1%
bAC-4c	1mg	10	18.5	1.196	73.3%
bAC-5a	0.65mg	17	10	1.299	91.7%
bAC-5b	0.75mg	10	11	1.37	90.0%
bAC-5c	0.6mg	10	18.5	1.319	96.8%
bAC-6a	0.6mg	17	10	1.246	61.1%
bAC-6b	0.6mg	10	11	1.175	73.2%
bAC-6c	1mg	10	18.5	1.232	61.0%
bAC-7a	0.6mg	17	10	1.193	70.9%
bAC-7b	0.6mg	10	11	1.319	77.1%
bAC-7c	0.6mg	10	18.5	1.173	85.2%
bAC-8a	0.6mg	17	10	1.266	93.6%
bAC-8b	0.6mg	10	11	1.186	76.3%
bAC-8c	0.6mg	10	18.5	1.168	72.5%
MTC-7b	0.65mg	10	11	1.312	86.9%
MTC-8b	0.8mg	10	11	1.213	80.9%

[a]Mole equivalent, [b]Polydispersity index (PDI) was obtained from gel permeation chromatography (GPC), [c]Yields of polymers were calculated from mass recovered after dialysis.

Supplementary Figures

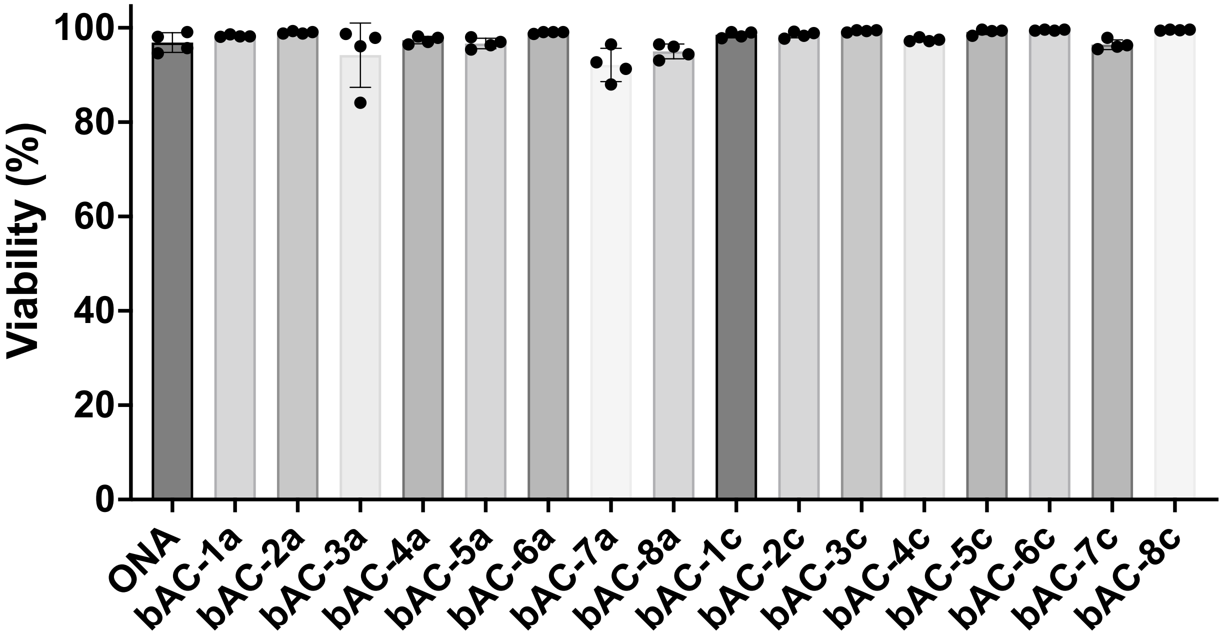

Supplementary Figure 1. Jurkat cell viability 24 hours after treatment with CART/eGFP mRNA complexes. Viability was calculated as the percentage of cells negative for Near-IR viability dye (n=4, bars represent mean with SD).

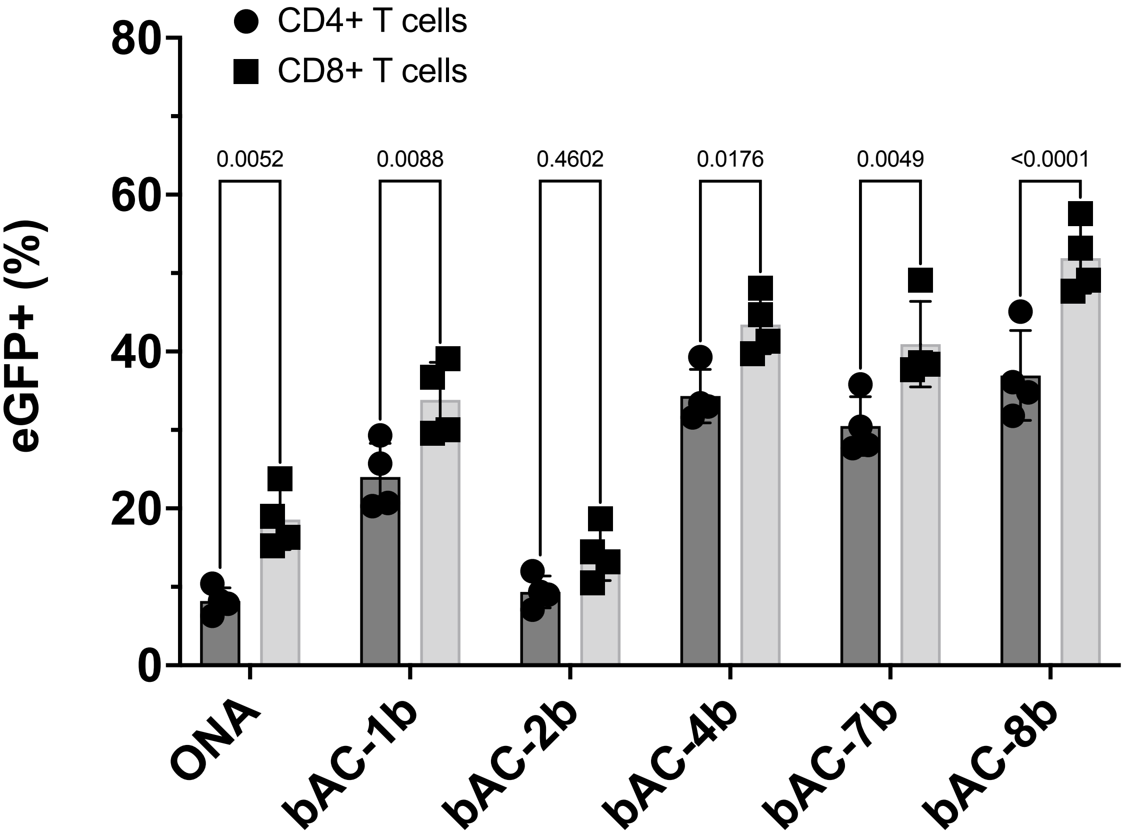

Supplementary Figure 2. Transfection of CD4+ and CD8+ primary T lymphocytes by CART/eGFP mRNA complexes (10:1 ratio) 24 hours after transfection (n=4, bars represent mean with SD). Statistical significance was calculated using 2way ANOVA.

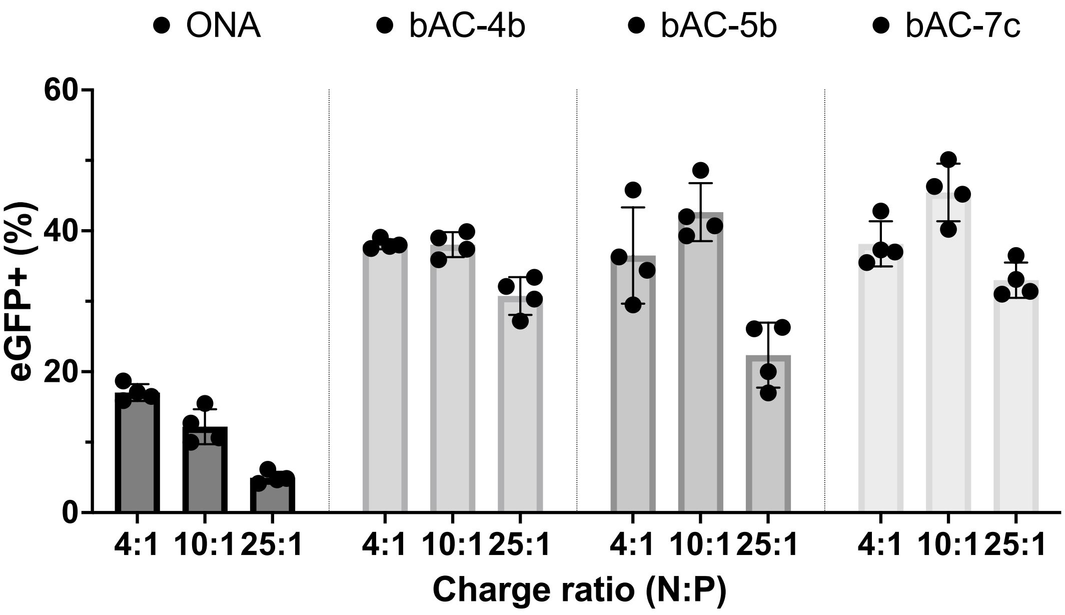

Supplementary Figure 3. Optimization of charge ratios for CART/eGFP mRNA delivery in primary T lymphocytes 24 hours after transfection (n=4, bars represent mean with SD).

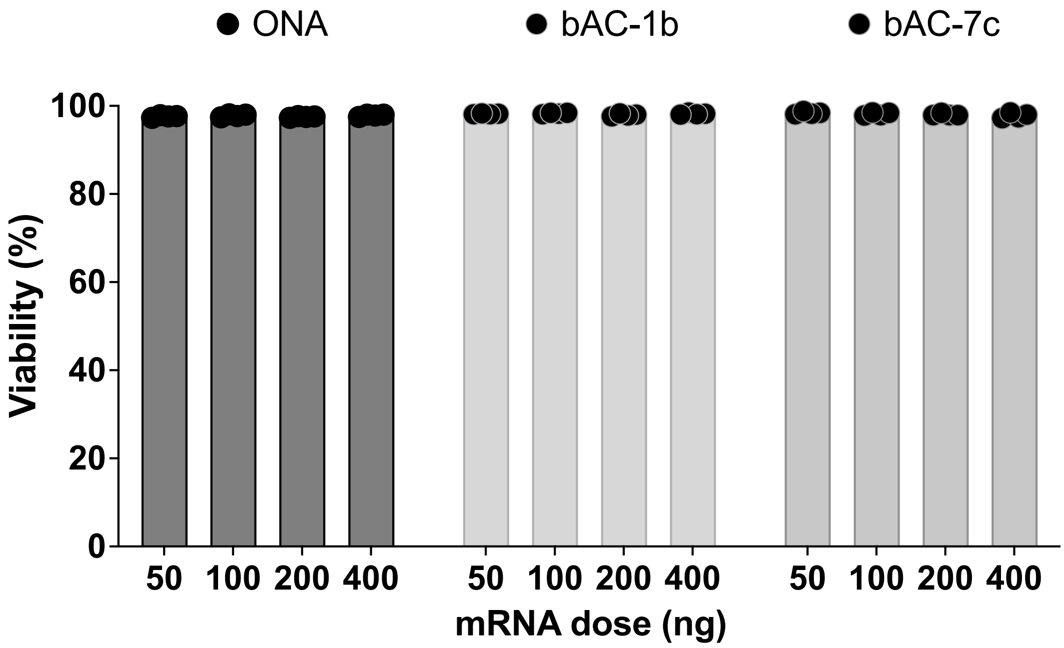

Supplementary Figure 4. Primary T lymphocyte viability 24 hours after treatment with CART complexed with 50 ng, 100 ng, 200 ng, and 400 ng eGFP mRNA (10:1 ratio). Viability was calculated as the percentage of cells negative for Near-IR viability dye (n=4, bars represent mean with SD).

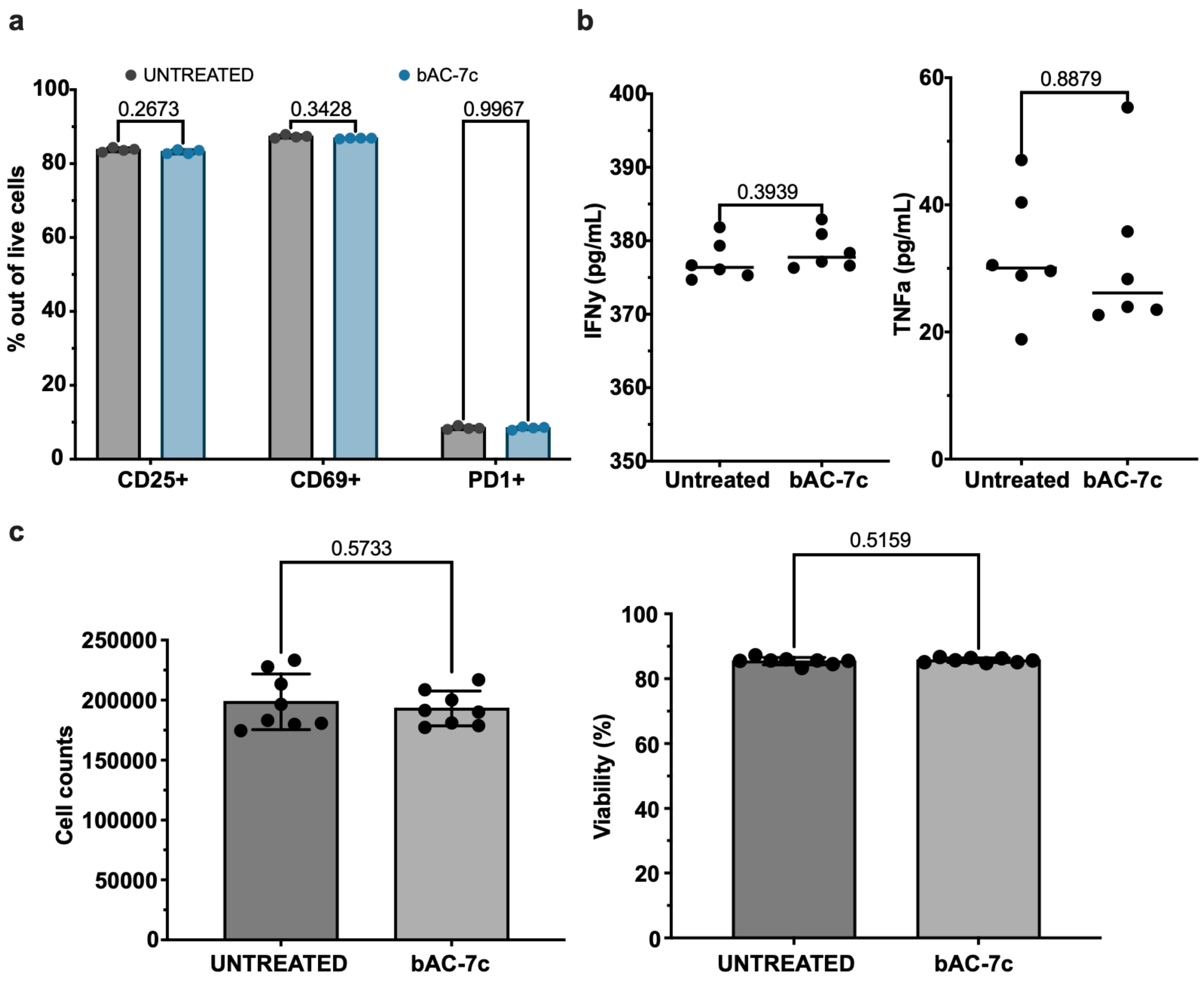

Supplementary Figure 5. Activation and functionality of primary human T cells after bAC-**7c** CART transfection. **a)** Protein expression levels of activation and exhaustion markers by flow cytometry (n=4, bars represent mean with SD). Statistical significance was calculated using 2way ANOVA. **b)** Inflammatory cytokine levels measured by ELISA (n=6, bar represents median). **c)** Cell viability and total cell counts after 24 hours of T cell activation with Dynabeads Human T-Activator CD3/CD28 (n=4, bars represent mean with SD). Statistical significance was calculated two-tailed unpaired t-test.

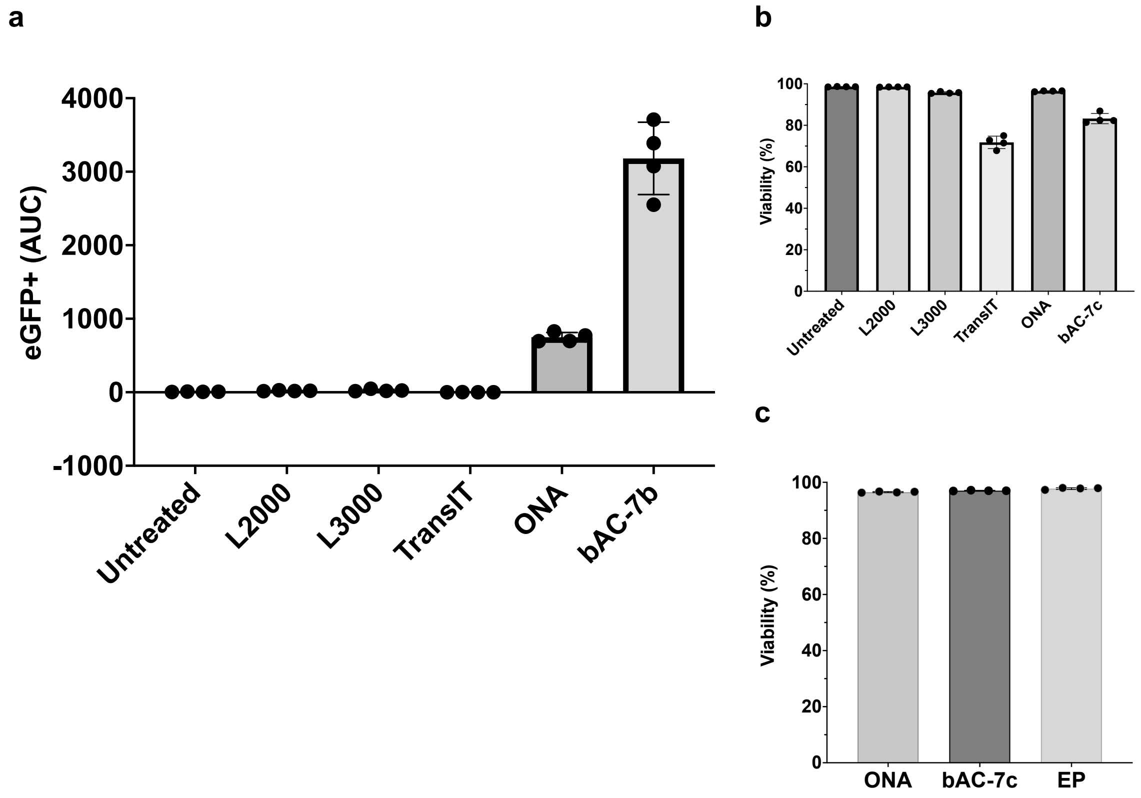

Supplementary Figure 6. bAC CARTs compared to commercially available transfection reagents. **a)** Area under the curve eGFP signal 24 hours after transfection of primary cells with commercially available transfection reagents (Lipofectamine 2000, Lipofectamine 3000, and TransIT-mRNA Transfection Reagent) and CART/eGFP mRNA complexes, ONA and bAC-7b (n=4, bars represent mean with SD). Primary T lymphocyte viability 24 hours after treatment with CART/eGFP mRNA complexes (ONA and bAC-7b, 10:1 N:P ratio) compared to **b)** commercially available transfection reagents and **c)** electroporation or EP (n=4, bars represent mean with SD).

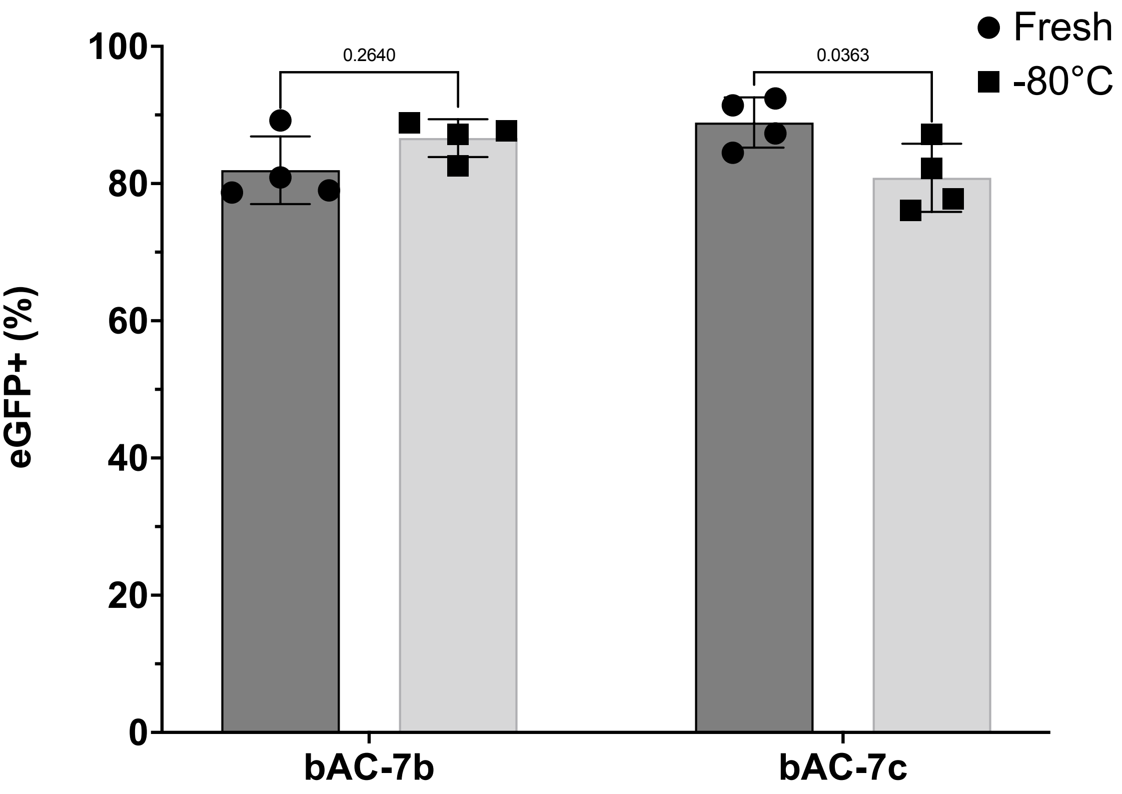

Supplementary Figure 7. Comparing delivery efficacy of CART/mRNA complexes (10:1 N:P ratio) prepared immediately before transfection (here referred as fresh) or stored at -80°C for 1 week after formulation (here referred as -80°C) (n=4, bars represent mean with SD). Statistical significance was calculated using 2way ANOVA.

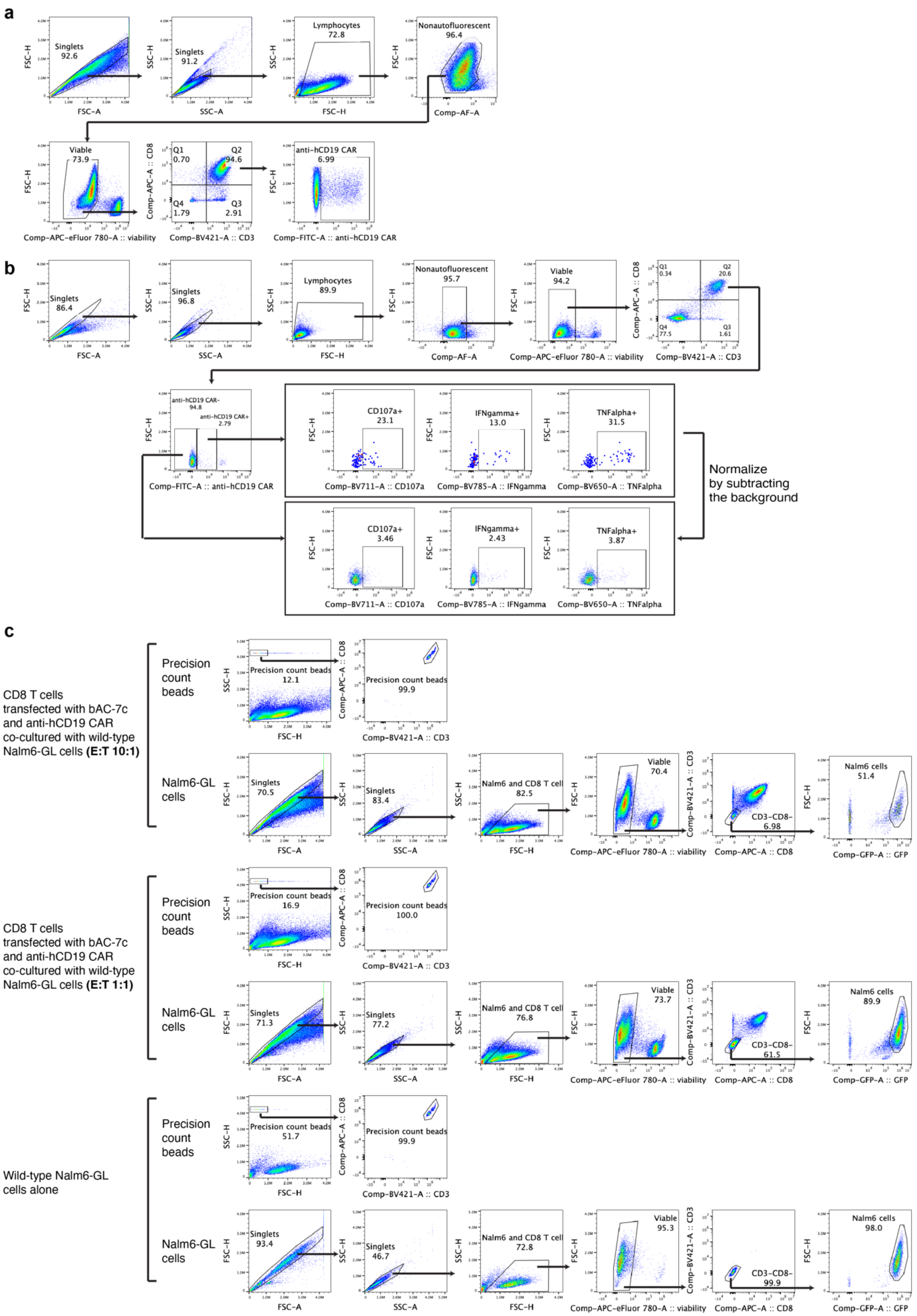
a
b
c
Singlets
92.6
Singlets
91.2
Lymphocytes
72.8
Nonautofluorescent
96.4
Viable
73.9
Q1
0.70
Q2
94.6
Q4
1.79
Q3
2.91
anti-hCD19 CAR
6.99
Singlets
86.4
Singlets
96.8
Lymphocytes
89.9
Nonautofluorescent
95.7
Viable
94.2
Q1
0.34
Q2
20.6
Q4
77.5
Q3
1.61
anti-hCD19 CAR-
94.8
anti-hCD19 CAR+
2.79
CD107a+
23.1
IFNgamma+
13.0
TNFalpha+
31.5
CD107a+
3.46
IFNgamma+
2.43
TNFalpha+
3.87
Normalize
by subtracting
the background
CD8 T cells
transfected with bAC-7c
and anti-hCD19 CAR
co-cultured with wild-type
Nalm6-GL cells (E:T 10:1)
Precision
count
beads
Precision count beads
12.1
Precision count beads
99.9
Nalm6-GL
cells
Singlets
70.5
Singlets
83.4
Nalm6 and CD8 T cell
82.5
Viable
70.4
CD3-CD8-
6.98
Nalm6 cells
51.4
CD8 T cells
transfected with bAC-7c
and anti-hCD19 CAR
co-cultured with wild-type
Nalm6-GL cells (E:T 1:1)
Precision count beads
16.9
Precision count beads
100.0
Singlets
71.3
Singlets
77.2
Nalm6 and CD8 T cell
76.8
Viable
73.7
CD3-CD8-
61.5
Nalm6 cells
89.9
Wild-type Nalm6-GL
cells alone
Precision count beads
51.7
Precision count beads
99.9
Singlets
93.4
Singlets
46.7
Nalm6 and CD8 T cell
72.8
Viable
95.3
CD3-CD8-
99.9
Nalm6 cells
98.0
FSC-A
FSC-H
SSC-A
SSC-H
Comp-AF-A
Comp-APC-eFluor 780-A :: viability
Comp-APC-A :: CD8
Comp-BV421-A :: CD3
Comp-FITC-A :: anti-hCD19 CAR
Comp-BV711-A :: CD107a
Comp-BV785-A :: IFNgamma
Comp-BV650-A :: TNFalpha
Comp-GFP-A :: GFP

d

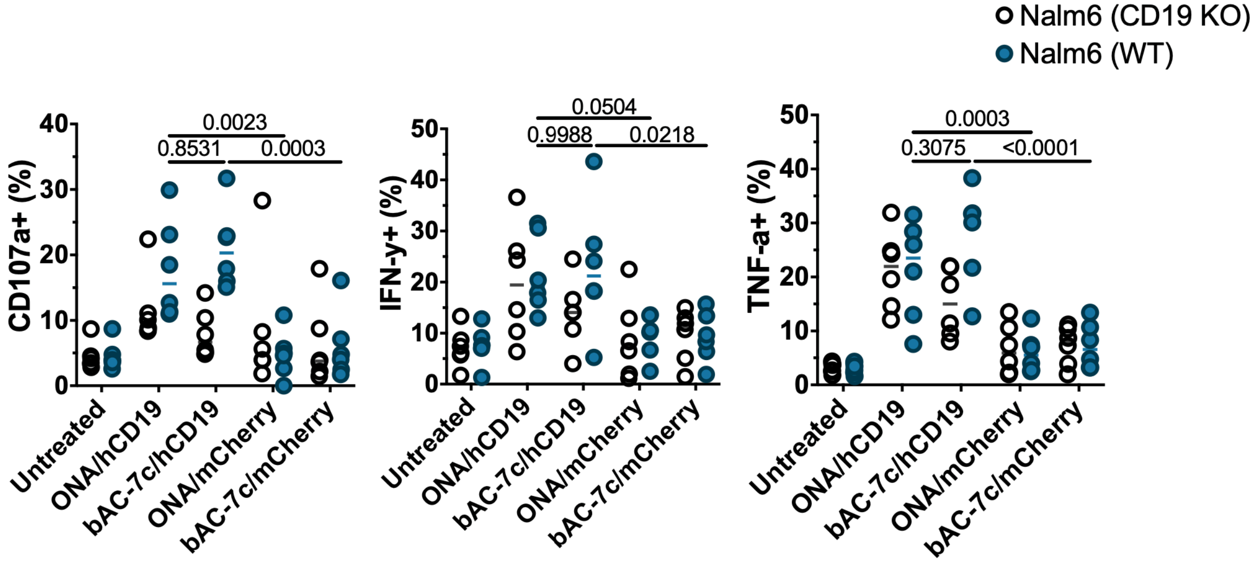

Supplementary Figure 8. a) Representative gating strategy of anti-hCD19 CAR expression of CD8 T cells transfected with ONA anti-hCD19 CAR. **b)** Gating strategy to analyze the functional marker expression in anti-hCD19 CAR+ and anti-hCD19 CAR- CD8 T cells (representative plots of CD8 T cells transfected with anti-hCD19 CAR and ONA co-cultured with wild-type Nalm6-GL cells). **c)** Representative gating strategy to gate on precision count beads and live Nalm6-GL cells after co-culture with anti-hCD19 expressing CAR T cells. **d)** Raw data for CD107a, IFN-γ, and TNF-α expression before normalization by untransfected CD8 T cells in the same co-culture well (n=6, bars represent median, Nalm6-GL cells labeled as Nalm6). Statistical significance was calculated using 2way ANOVA.

a

b

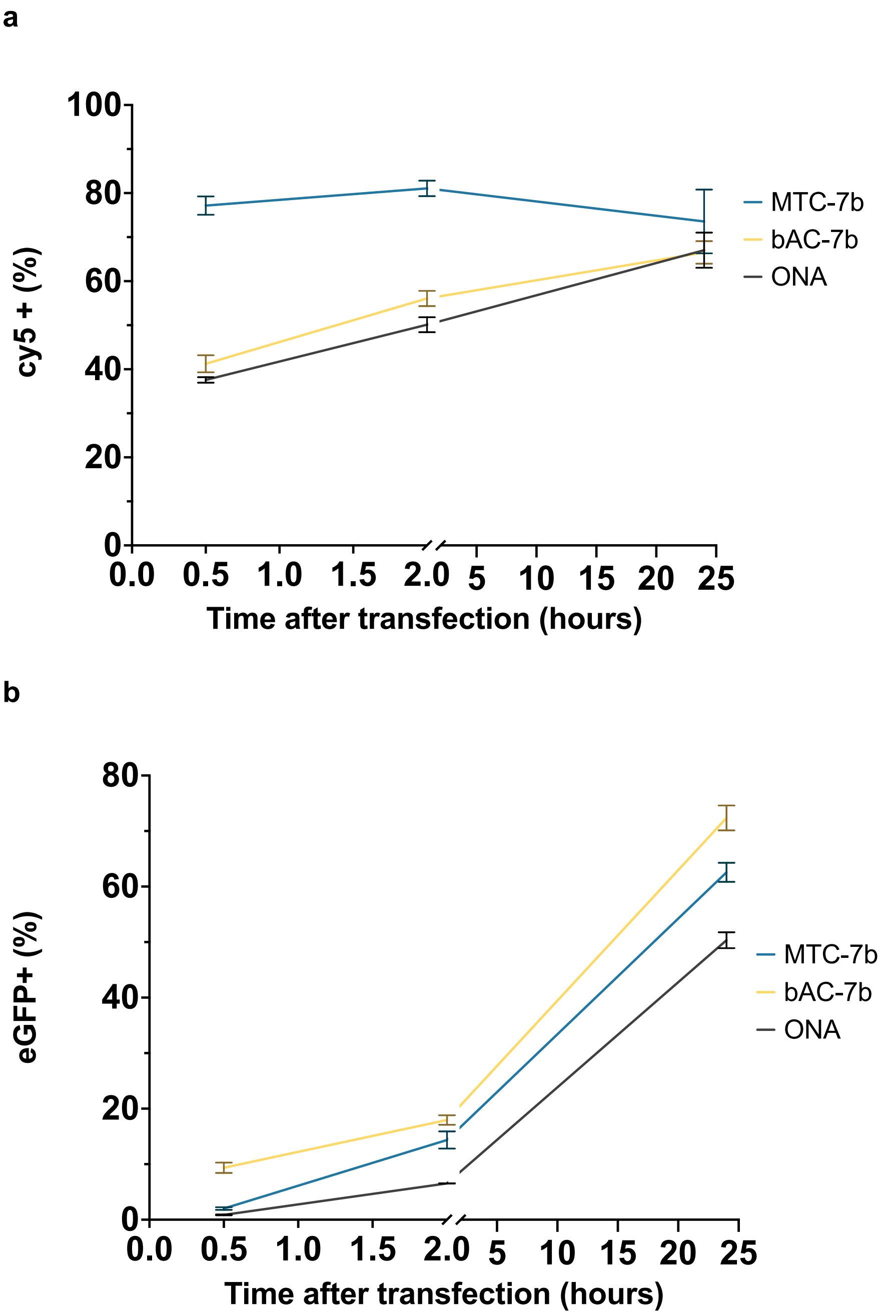

Supplementary Figure 9. Kinetics of Cy5+ labeled mRNA **a**) uptake and **b**) eGFP mRNA protein expression in Jurkat cells when CARTs were formulated with mRNA at 10:1 N:P ratio (n=3, bars represent SEM)

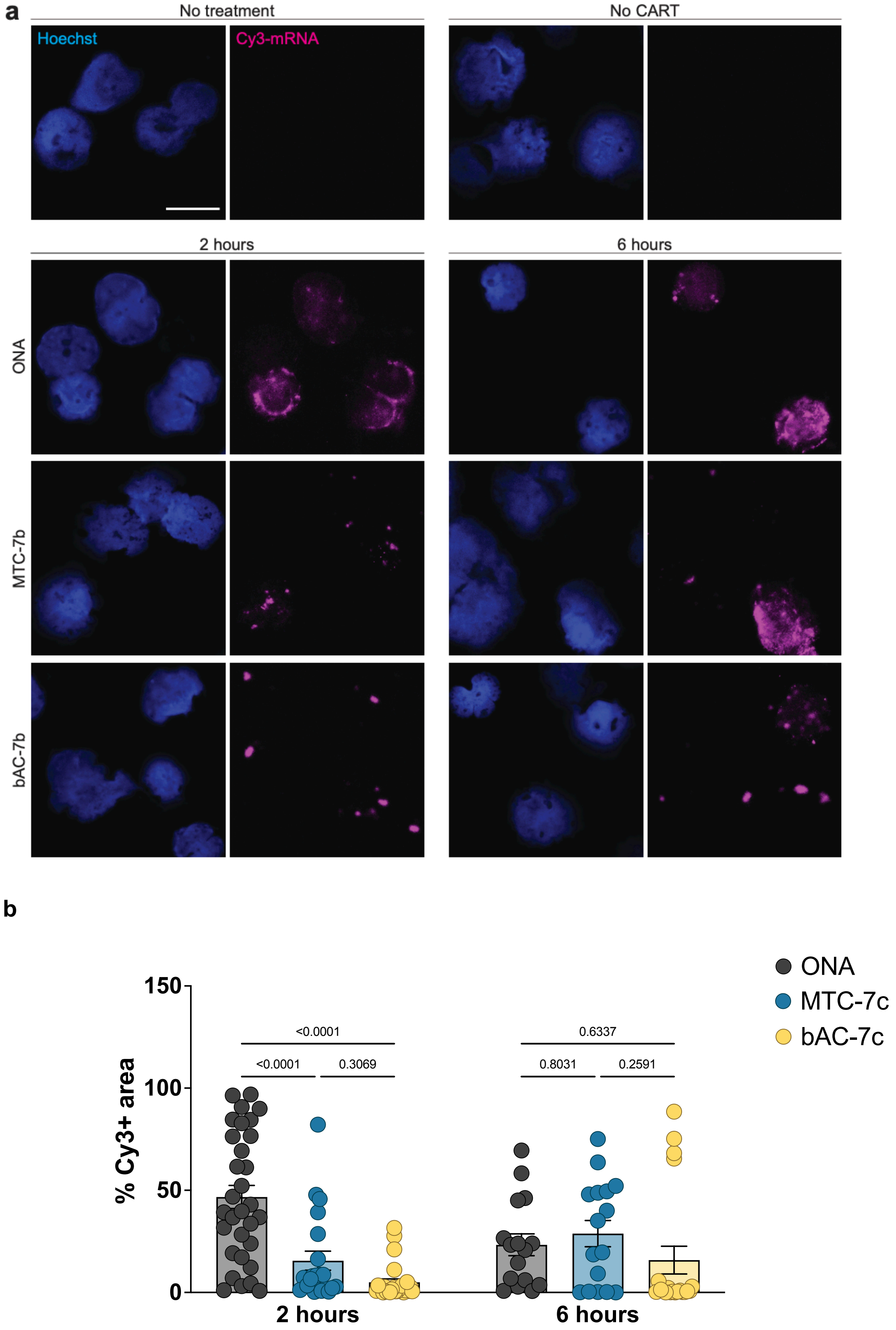
a
No treatment
No CART
Hoechst
Cy3-mRNA
2 hours
6 hours
ONA
MTC-7b
bAC-7b
b
% Cy3+ area
150
100
50
0
<0.0001
<0.0001
0.3069
0.6337
0.8031
0.2591
2 hours
6 hours
ONA
MTC-7c
bAC-7c

Supplementary Figure 10. Cell localization of Cy3-labeled mRNA. **a)** Confocal microscopy of Jurkat cells fixed 2 or 6 hours after transfection with ONA, MTC-7c, or bAC-7c CART. **b)** Quantification of Cy3-mRNA+ area by cell. N>20 cells per condition over 5 random fields, bars represent mean with SEM. Statistical significance was calculated using 2way ANOVA.

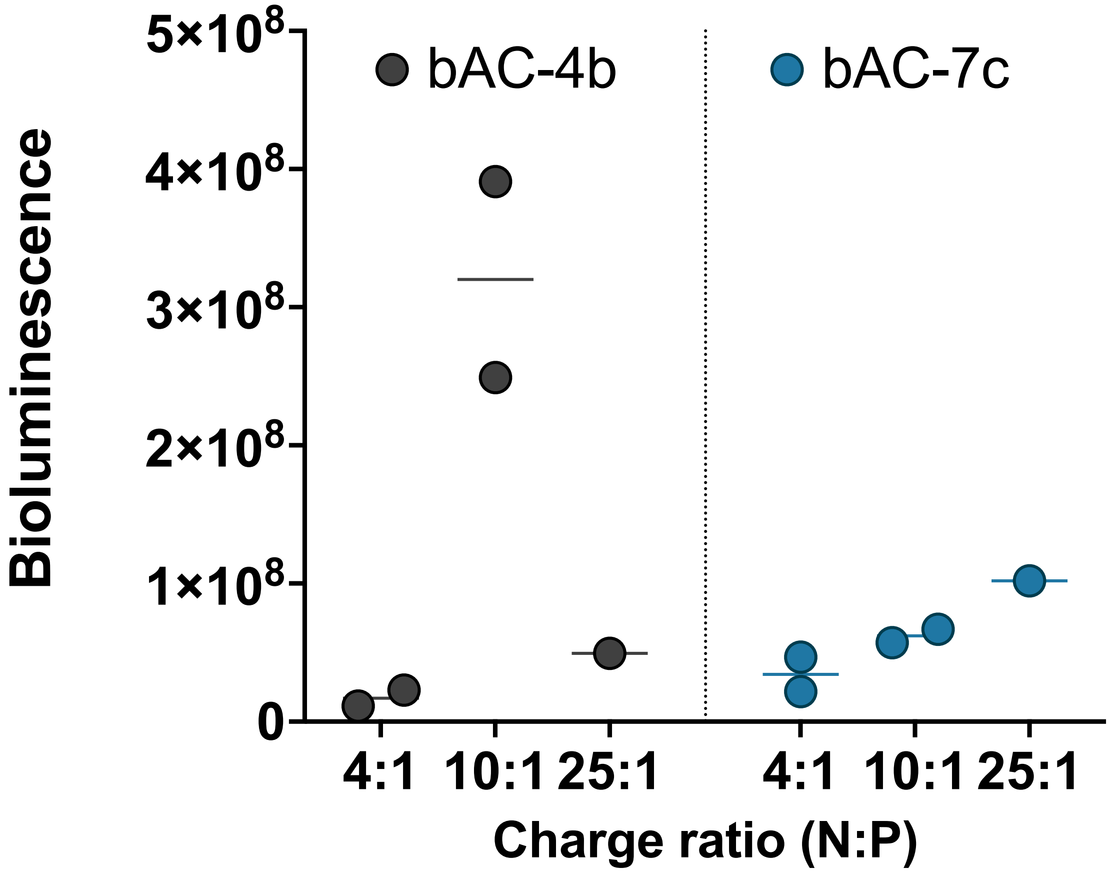

Supplementary Figure 11. Delivery efficacy of bAC-4b and bAC-7c CARTs when complexed with luciferase mRNA at 4:1, 10:1, and 25:1 charge ratios (N:P). Optical imaging was performed 6 hours after i.v. injection (n=2, bars represent median).

a

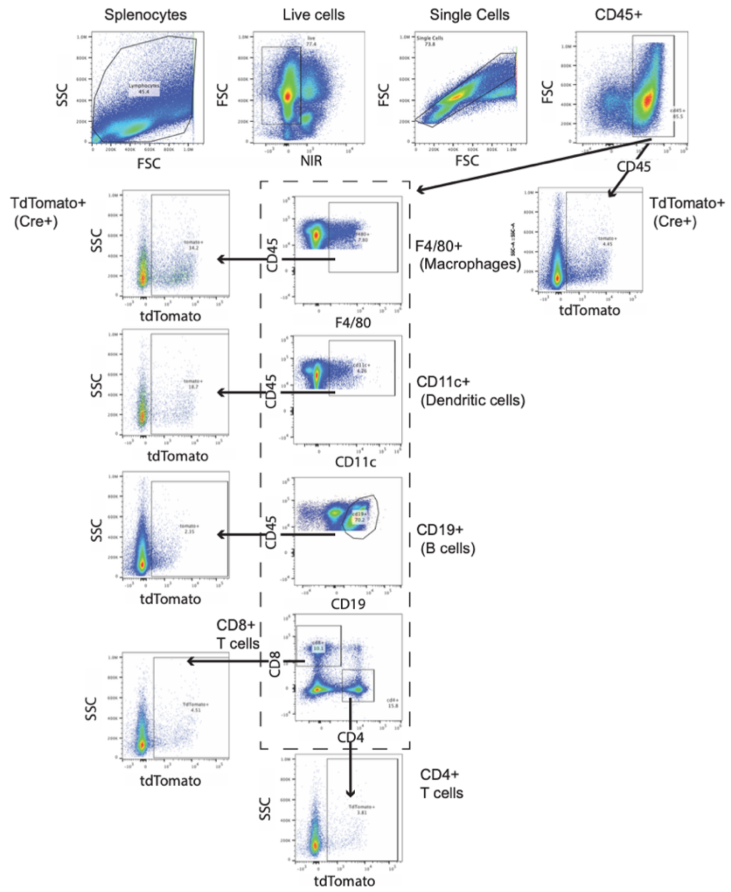

b

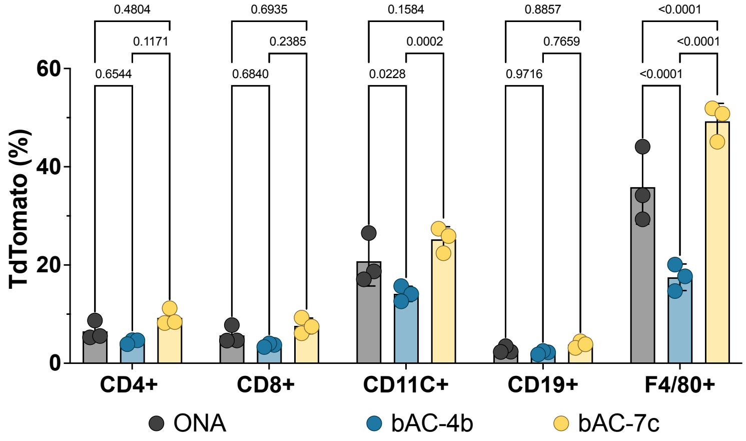

Supplementary Figure 12. **a)** Gating strategy for identification of immune cells in mouse splenocytes after Cre-mediated recombination in Ai14 mice. **b)** Quantification of Cre recombination in distinct splenocytes subsets. (n=3, bars represent mean with SD). Statistical significance was calculated using 2way ANOVA. Flow cytometry analysis of spleens was performed 48h after i.v. injection of CART/mRNA complexes (10:1 N:P ratio).

a **b**

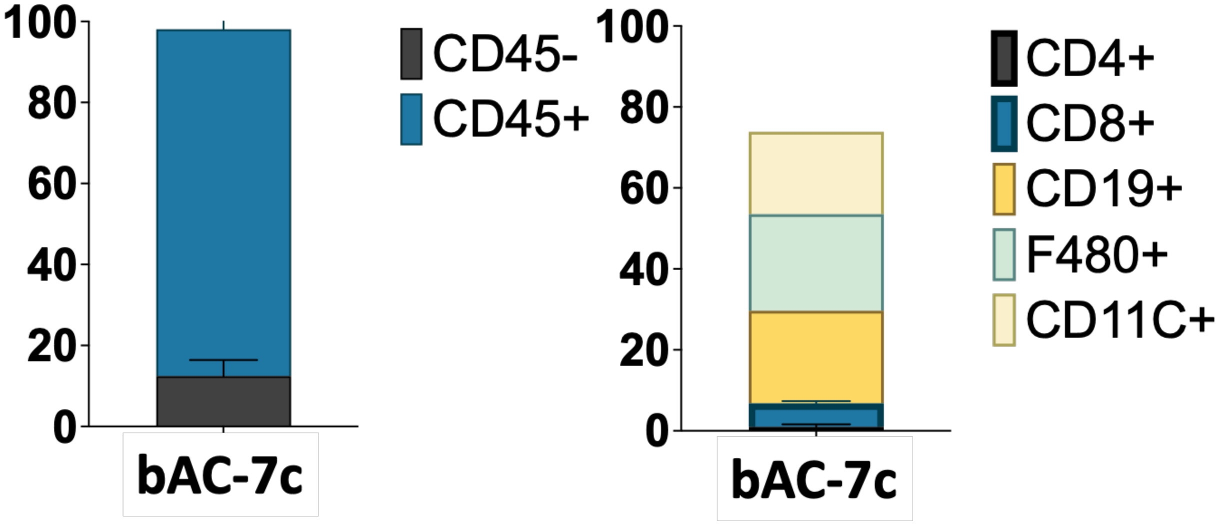

Supplementary Figure 13. Distribution of mRNA in the spleen after delivery with bAC-7c CARTs. **a)** Percentage of Cy5-mRNA+ cells out of total live cells **b)** Percentage of Cy5-mRNA+ cells out of CD45+ cells (n=3, bars represent mean with SD).

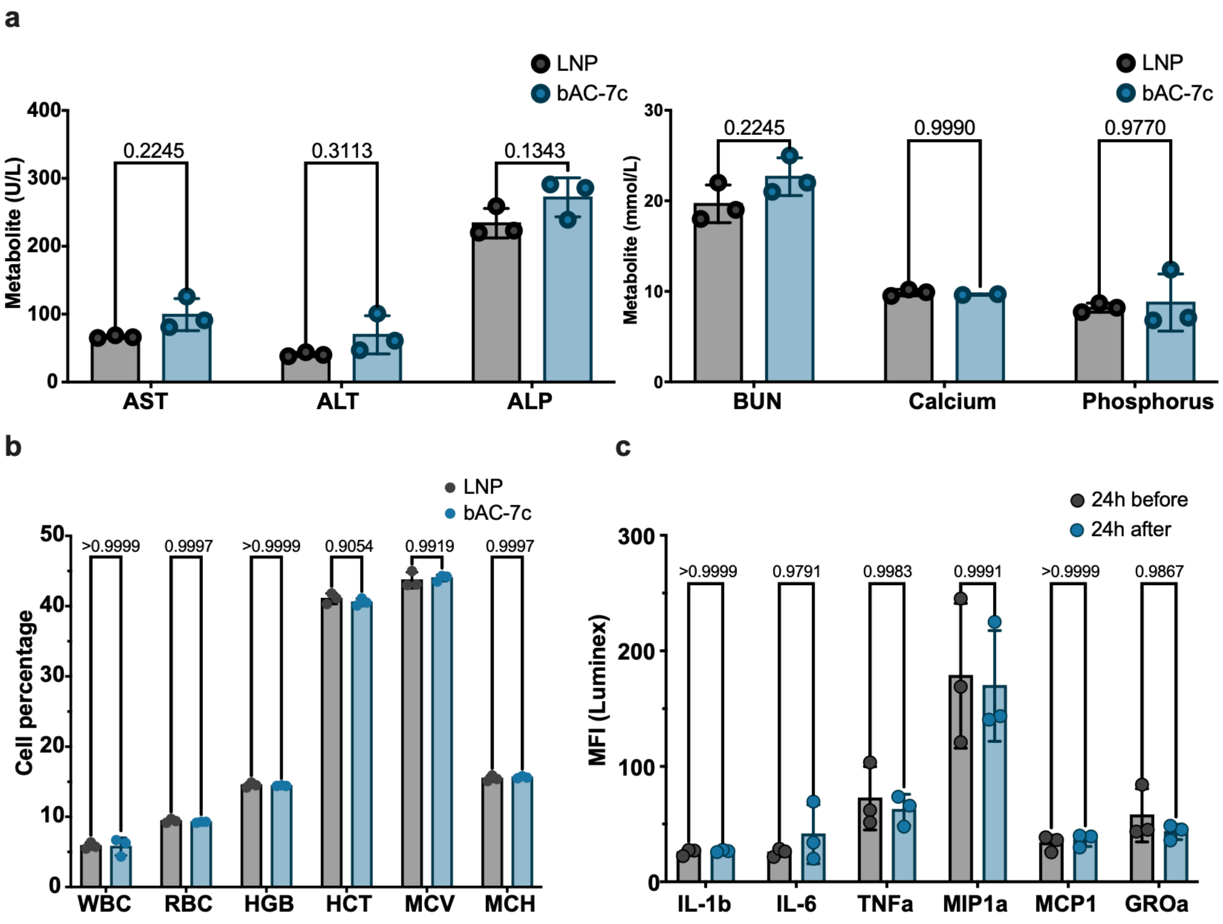

Supplementary Figure 14. Preclinical toxicology blood analysis 24 hours after delivery of 7.5 mg of luciferase mRNA formulated with bAC CART compared to MC3 LNP. **a)** Blood metabolites, **b)** blood counts and **c)** inflammatory cytokines 24 hours before and after delivery of luciferase mRNA with bAC-7c CART (n=3, bars represent mean with SD). Statistical significance was calculated using 2way ANOVA.

NMR spectra

Supplementary Figure 15. NMR of Lipidic acid 3

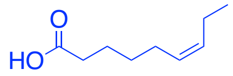

Lipidic acid 3, ^{1}H NMR (400 MHz, $CDCl_3$)

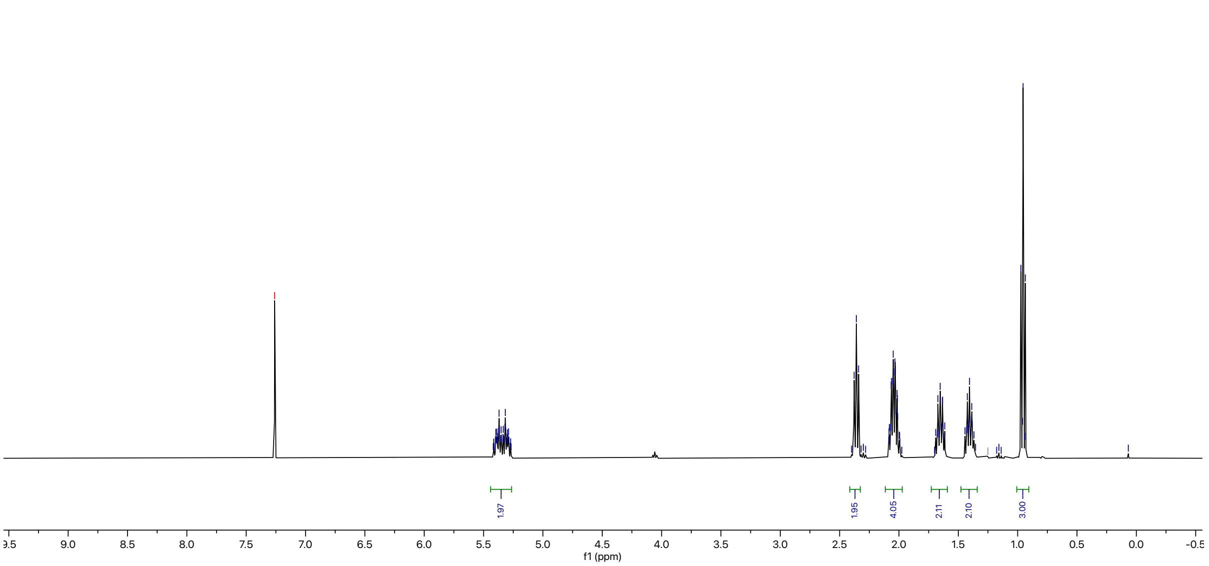

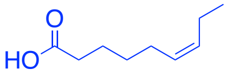

Lipidic acid 3, ^{13}C NMR (100 MHz, $CDCl_3$)

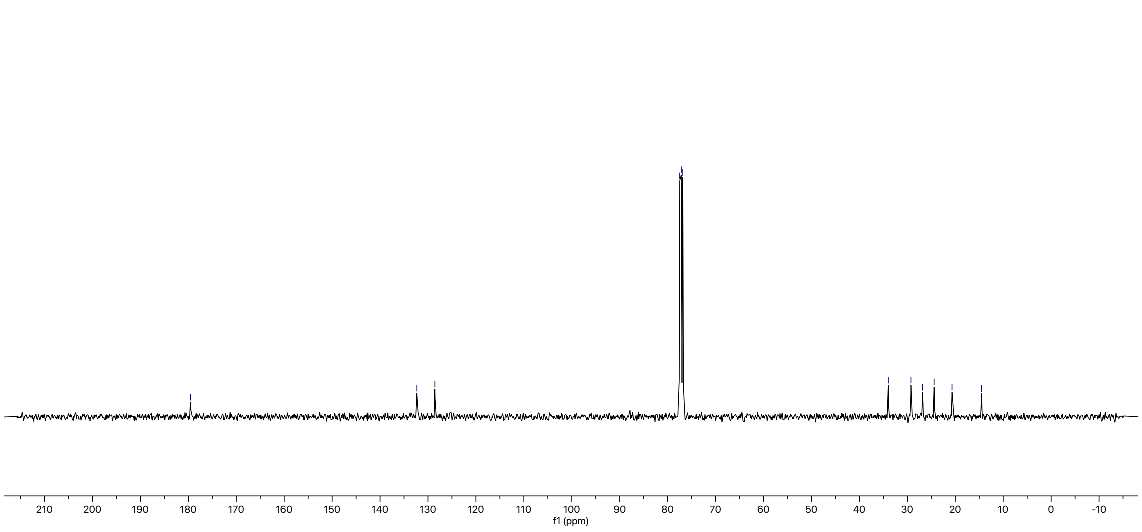

Supplementary Figure 16. NMR of Lipidic acid 4

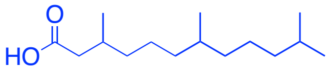

Lipidic acid 4, ¹H NMR (400 MHz, $CDCl_3$)

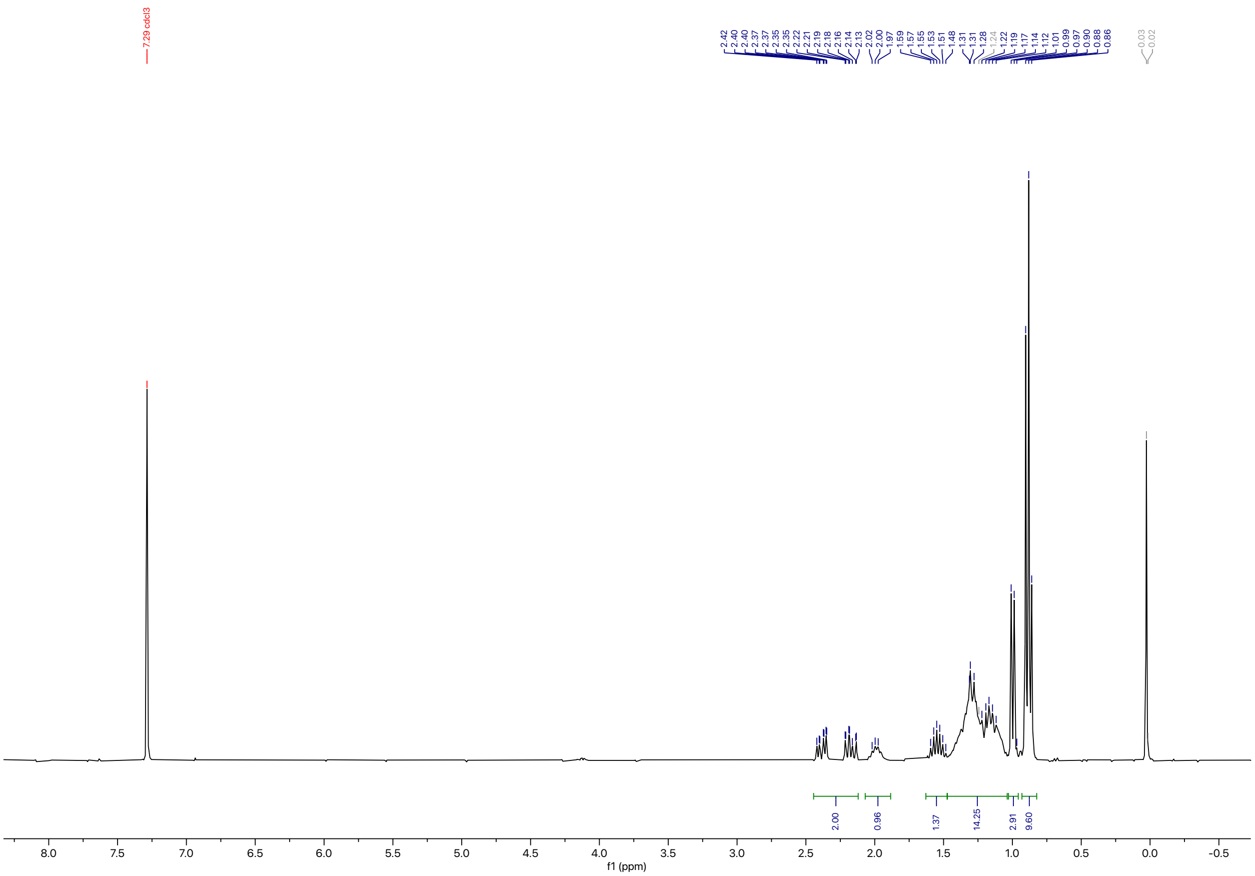

Supplementary Figure 17. NMR of Lipidic acid 5

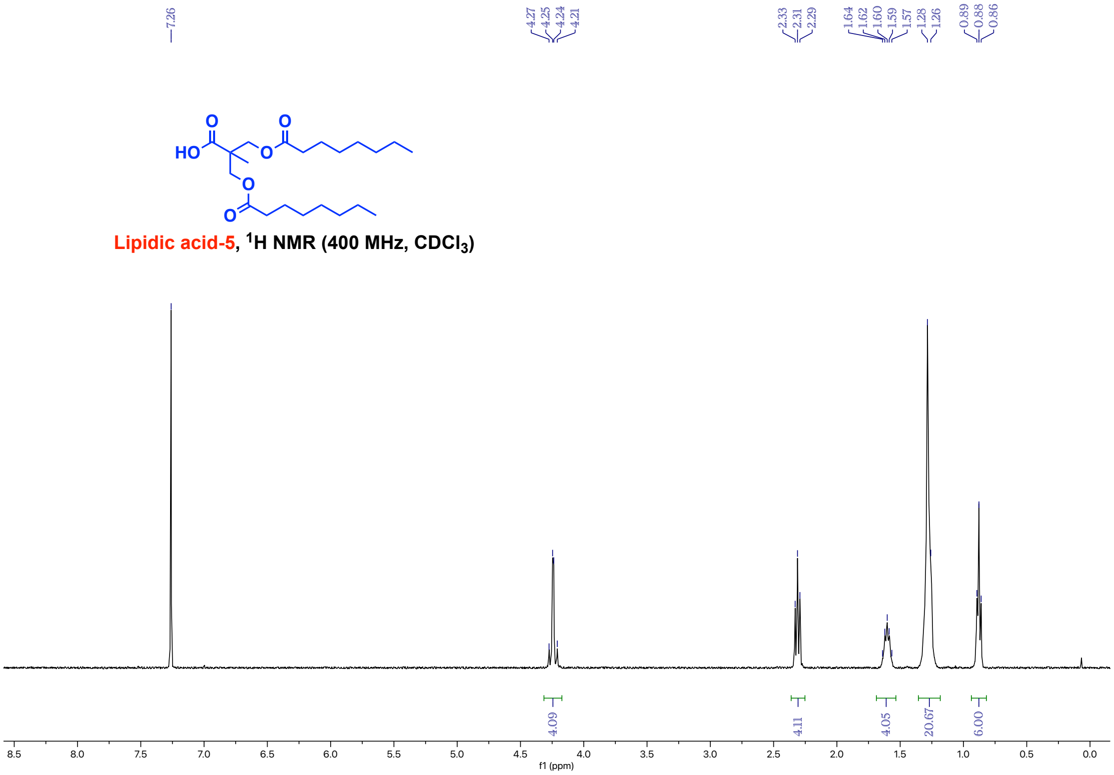

Lipidic acid-5, ^{1}H NMR (400 MHz, $CDCl_3$)

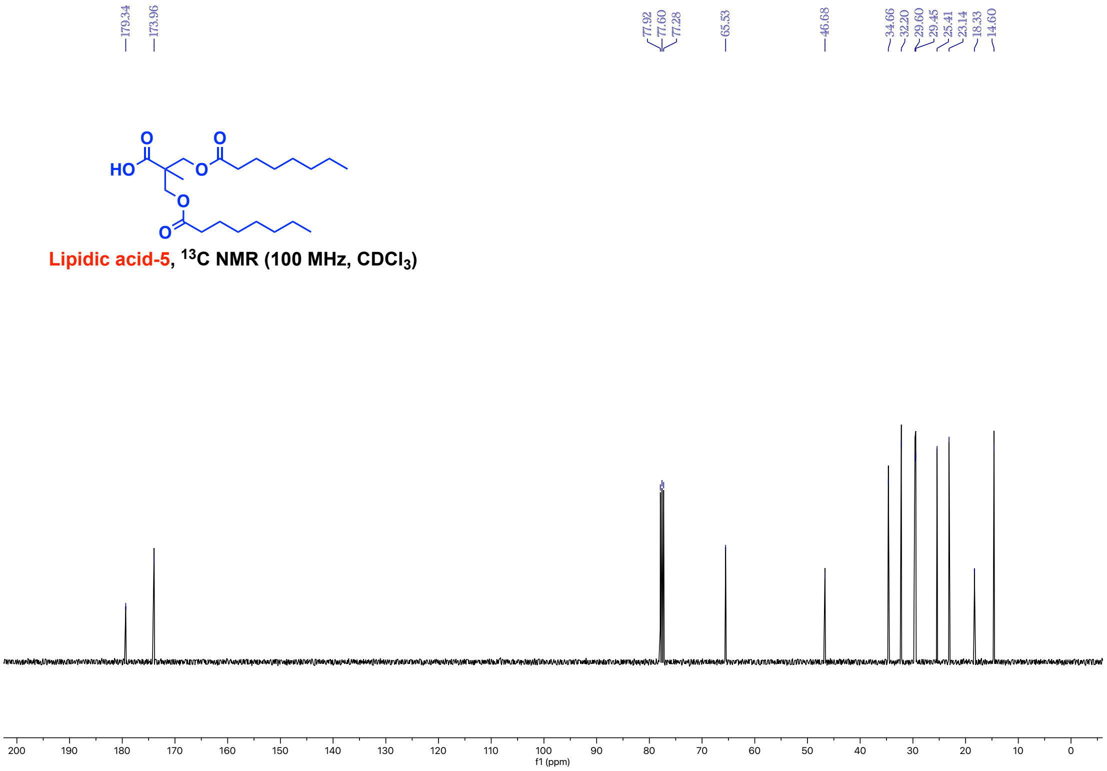

Lipidic acid-5, ^{13}C NMR (100 MHz, $CDCl_3$)

Supplementary Figure 18. NMR of Lipidic acid 6

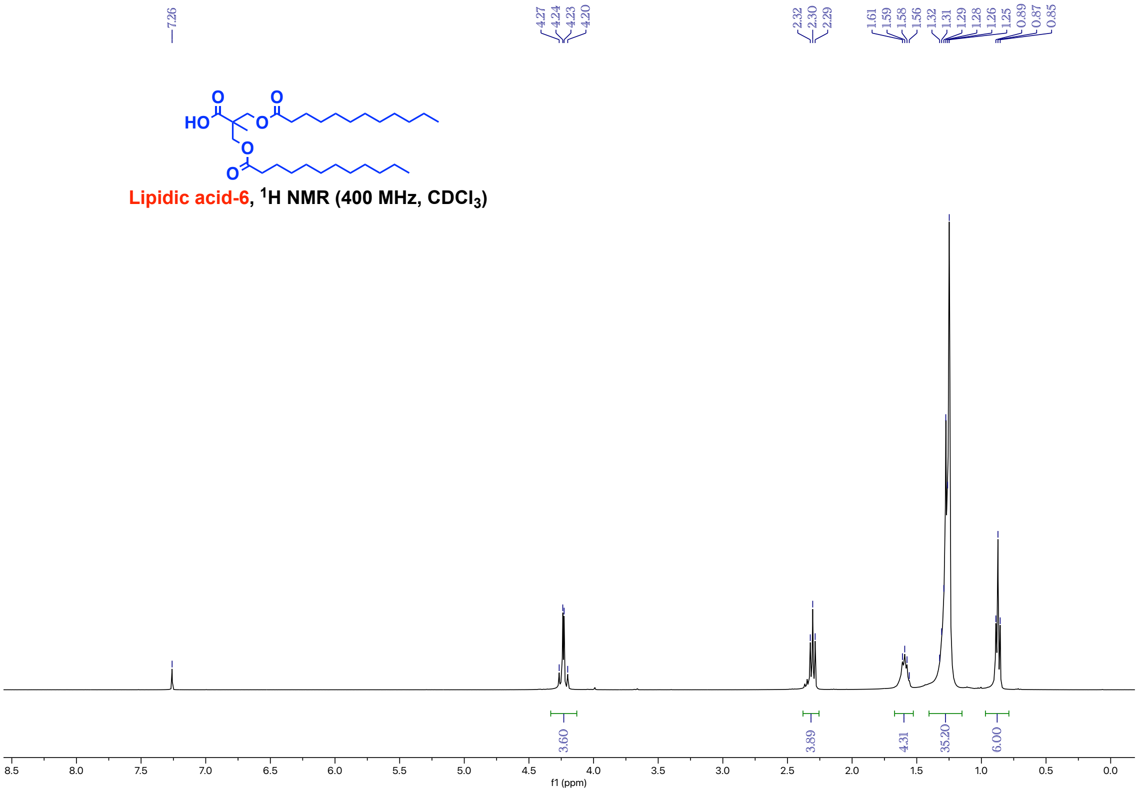

Lipidic acid-6, ^{1}H NMR (400 MHz, $CDCl_3$)

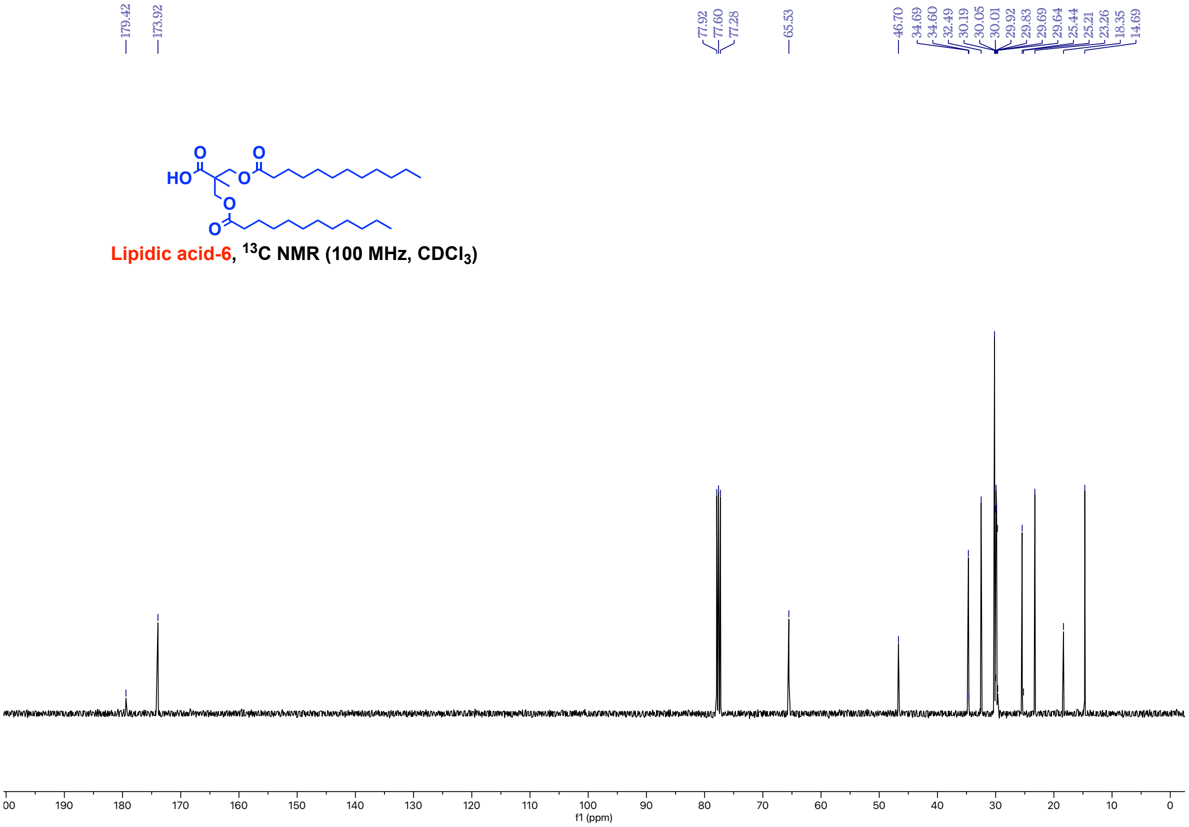

Lipidic acid-6, ^{13}C NMR (100 MHz, $CDCl_3$)

Supplementary Figure 19. NMR of Lipidic alcohol 7

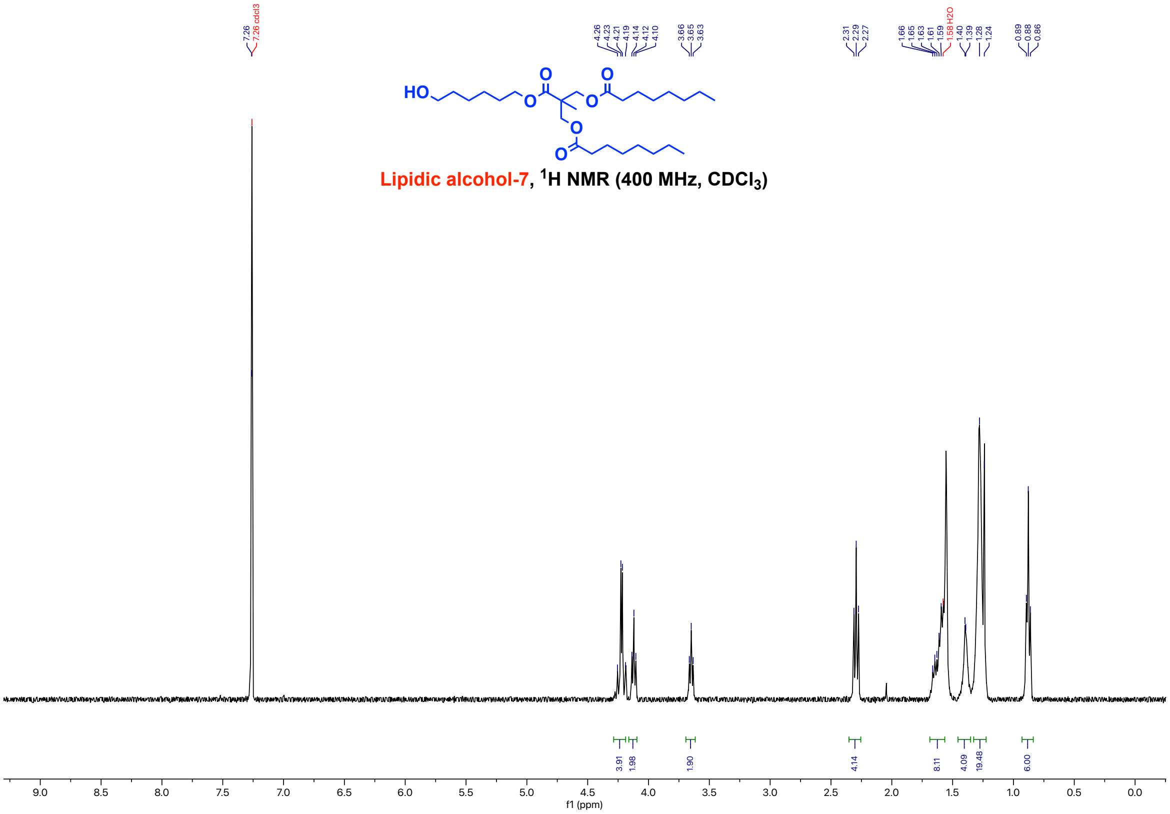

Lipidic alcohol-7, ^{1}H NMR (400 MHz, $CDCl_3$)

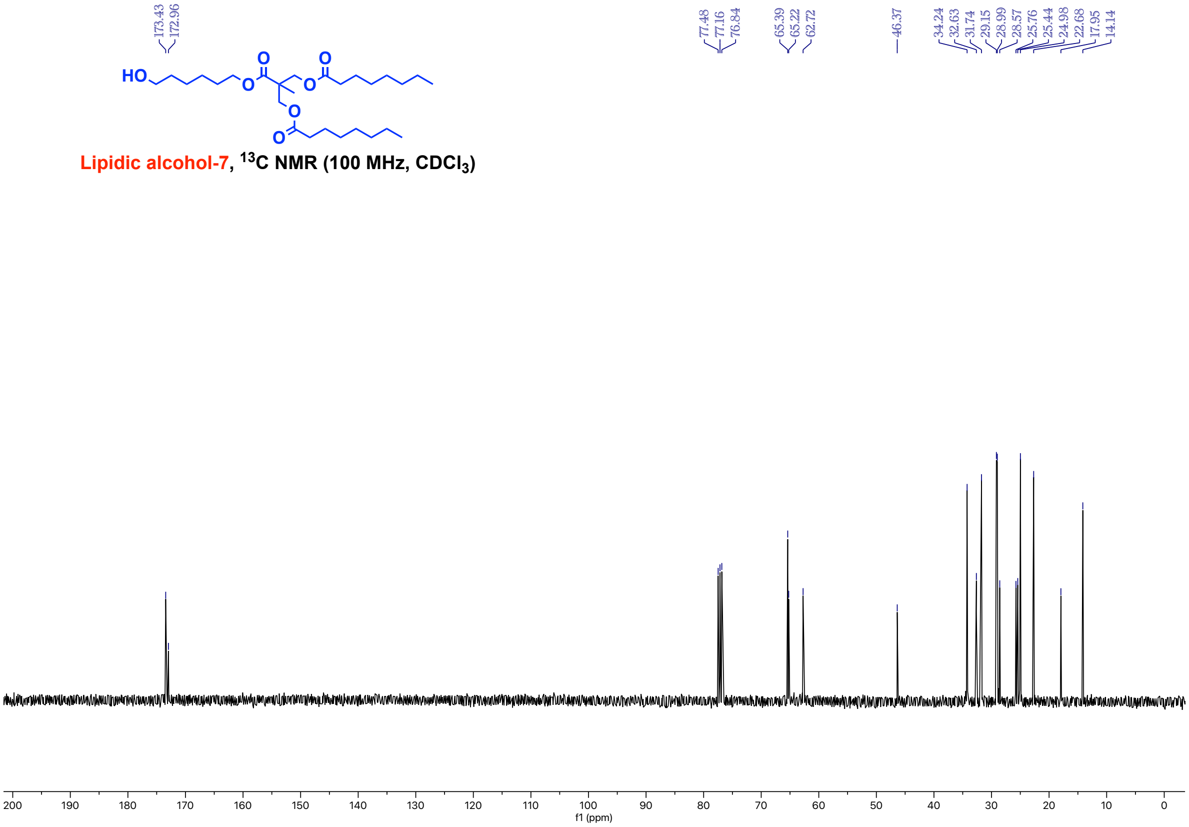

Lipidic alcohol-7, ^{13}C NMR (100 MHz, $CDCl_3$)

Supplementary Figure 20. NMR of Lipidic alcohol 8

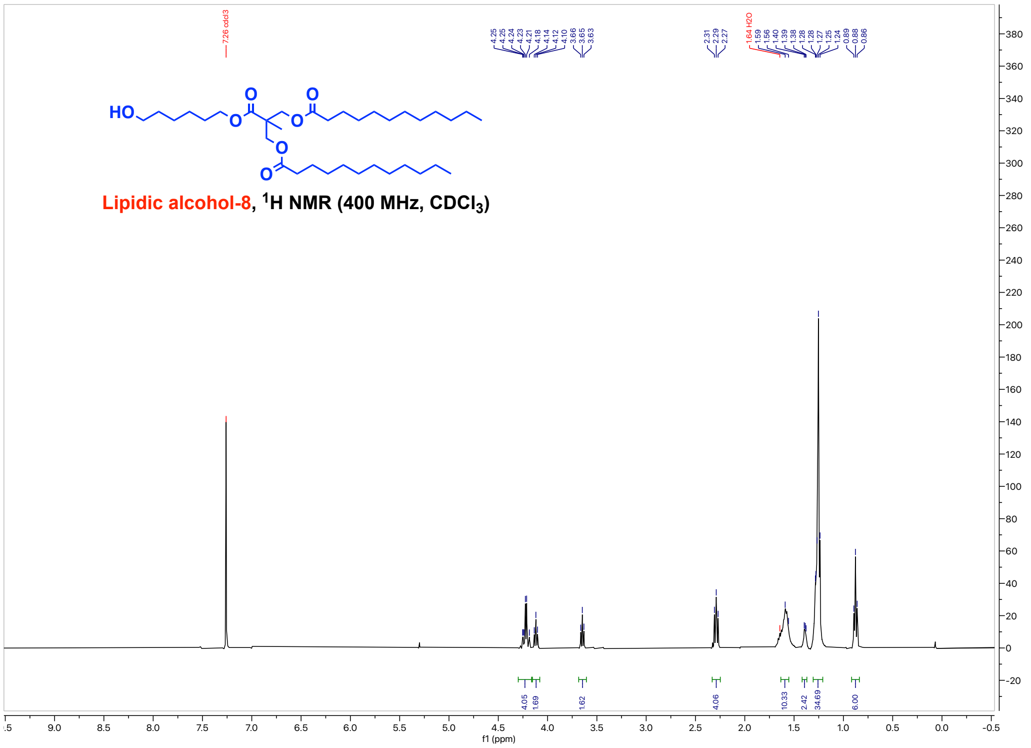

Lipidic alcohol-8, ^{1}H NMR (400 MHz, $CDCl_3$)

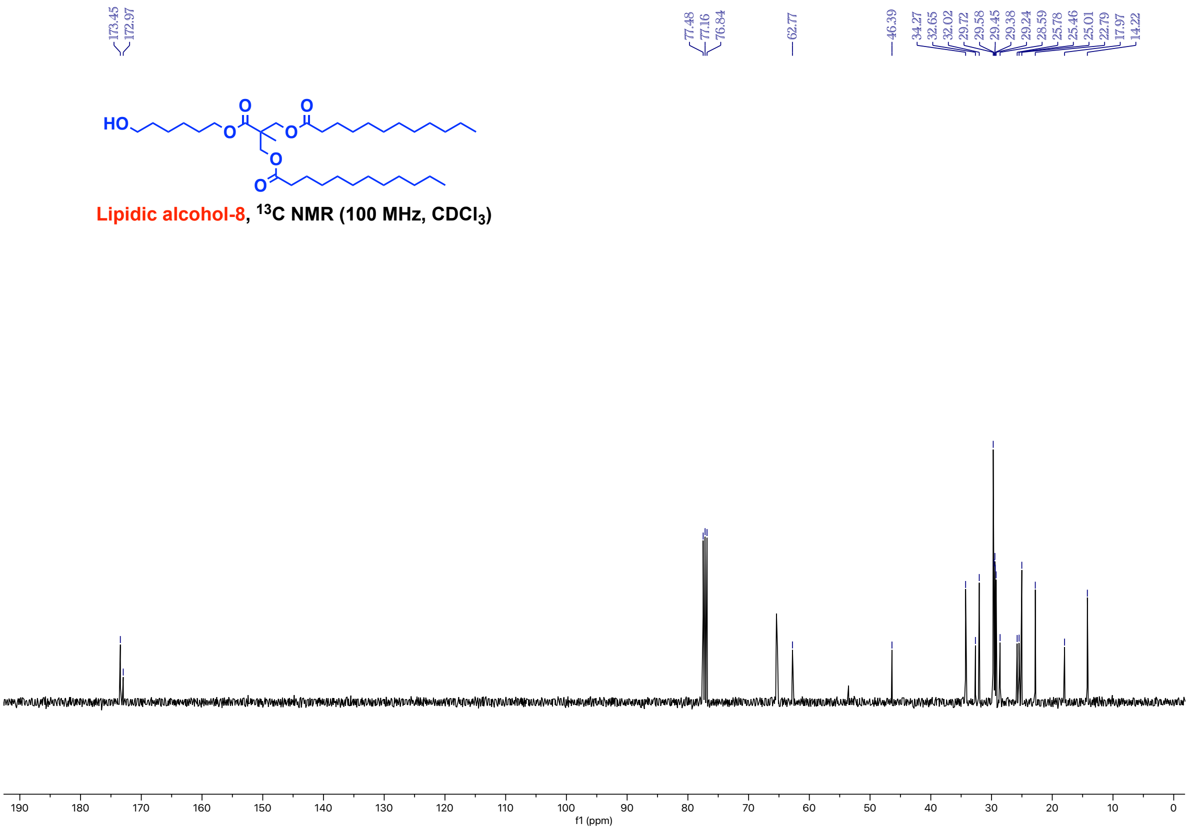

Lipidic alcohol-8, ^{13}C NMR (100 MHz, $CDCl_3$)

Supplementary Figure 21. NMR of Lipidic acid 7

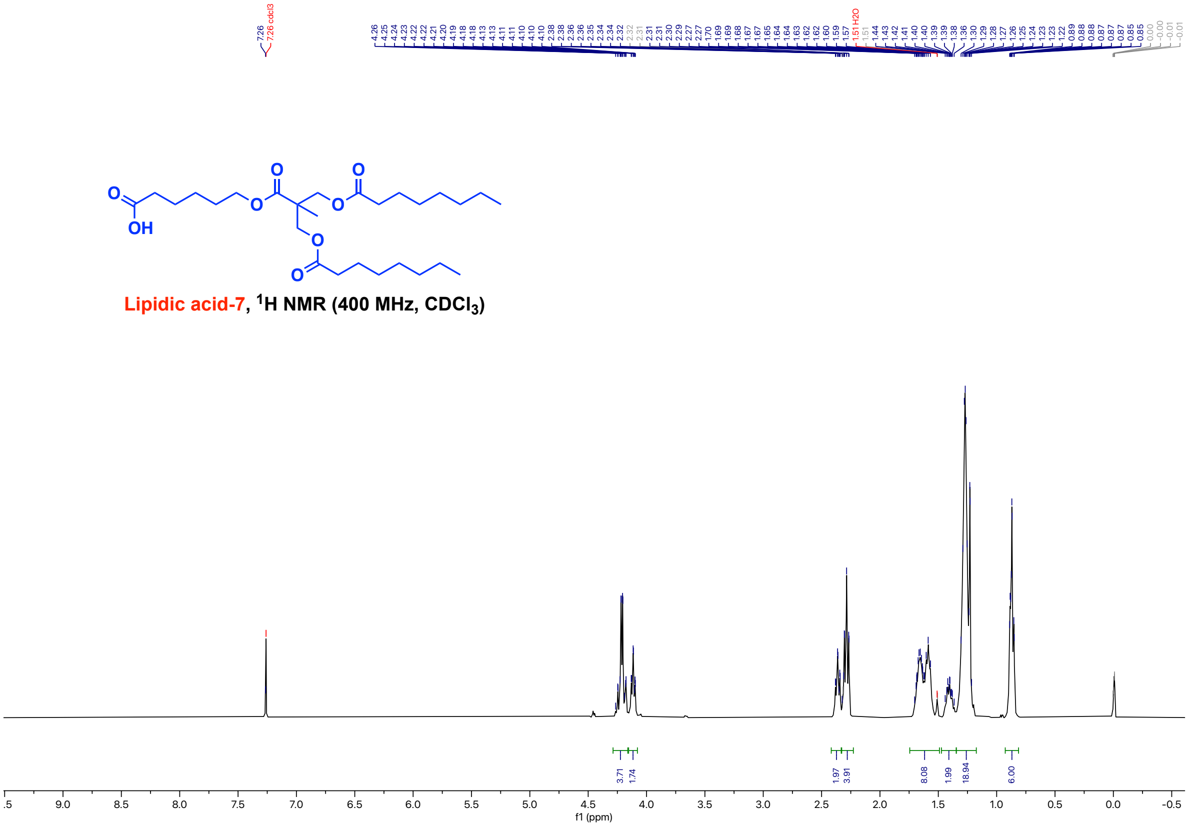

Lipidic acid-7, ^{1}H NMR (400 MHz, $CDCl_3$)

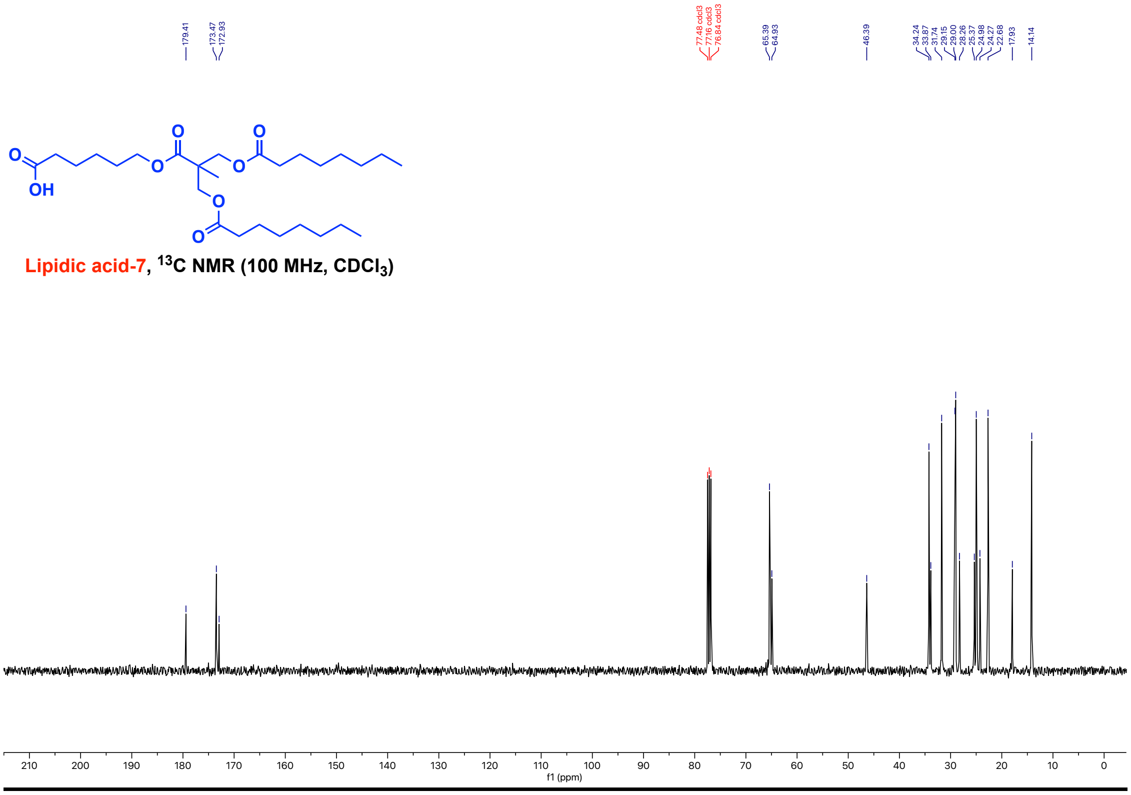

Lipidic acid-7, ^{13}C NMR (100 MHz, $CDCl_3$)

Supplementary Figure 22. NMR of Lipidic acid 8

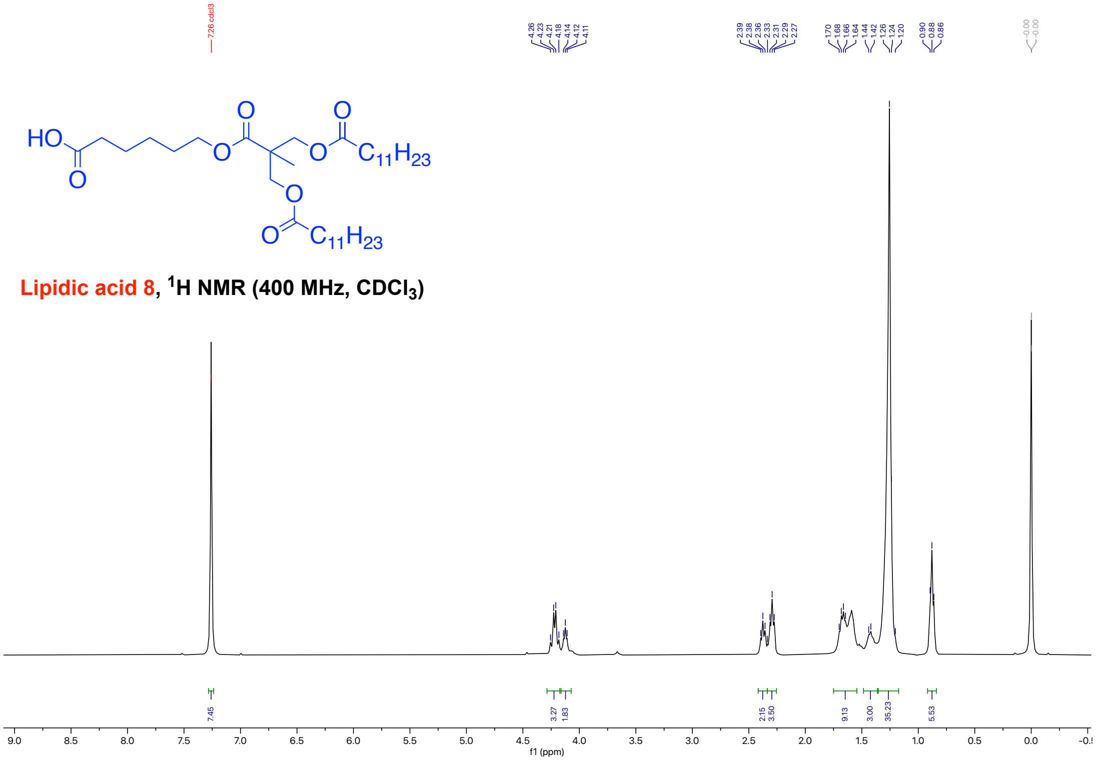

Lipidic acid 8, **^{1}H NMR (400 MHz, $CDCl_3$)**

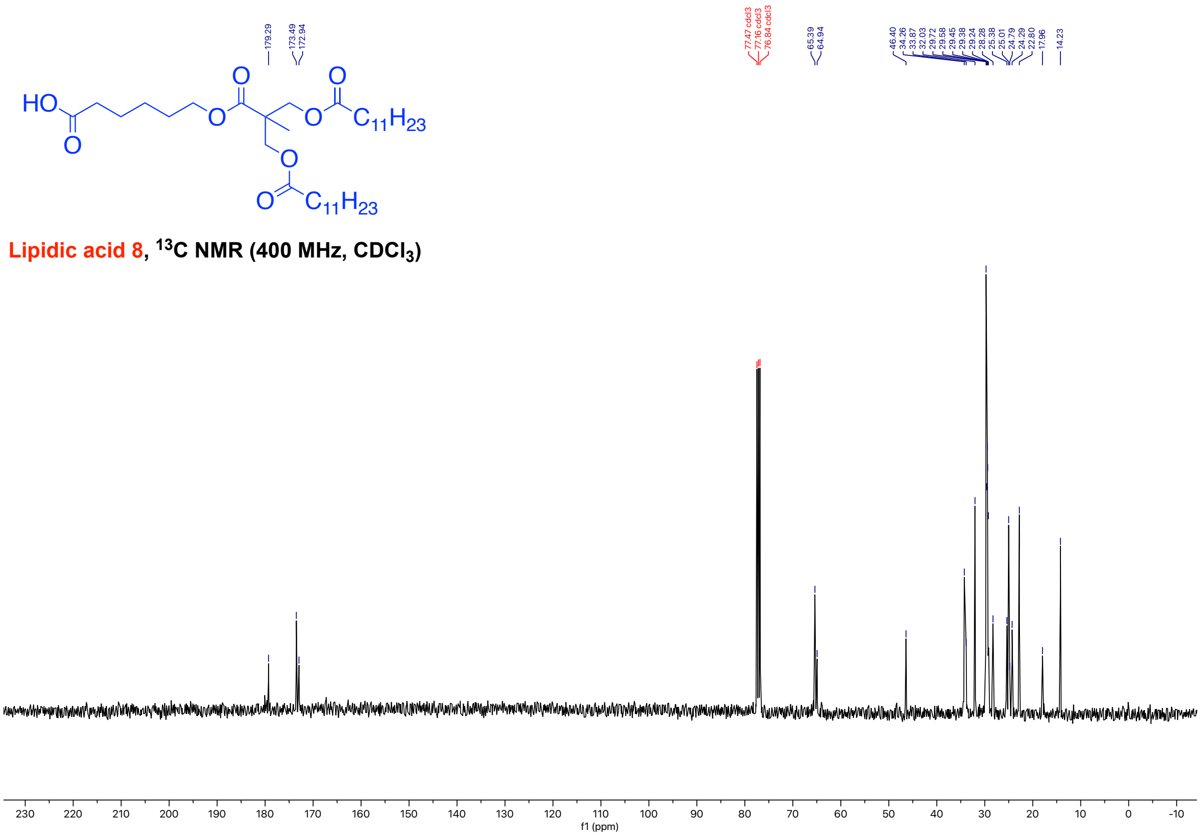

Lipidic acid 8, **^{13}C NMR (400 MHz, $CDCl_3$)**

Supplementary Figure 23. NMR of bAC-1

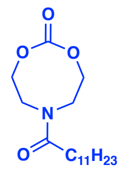

bAC-1, ^{1}H NMR (400 MHz, $CDCl_3$)

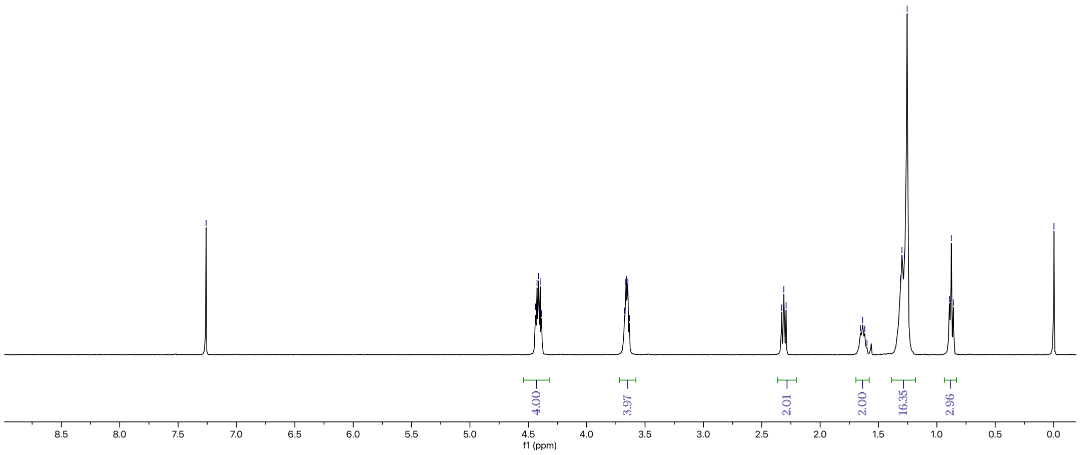

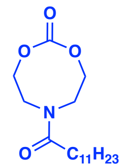

bAC-1, ^{13}C NMR (100 MHz, $CDCl_3$)

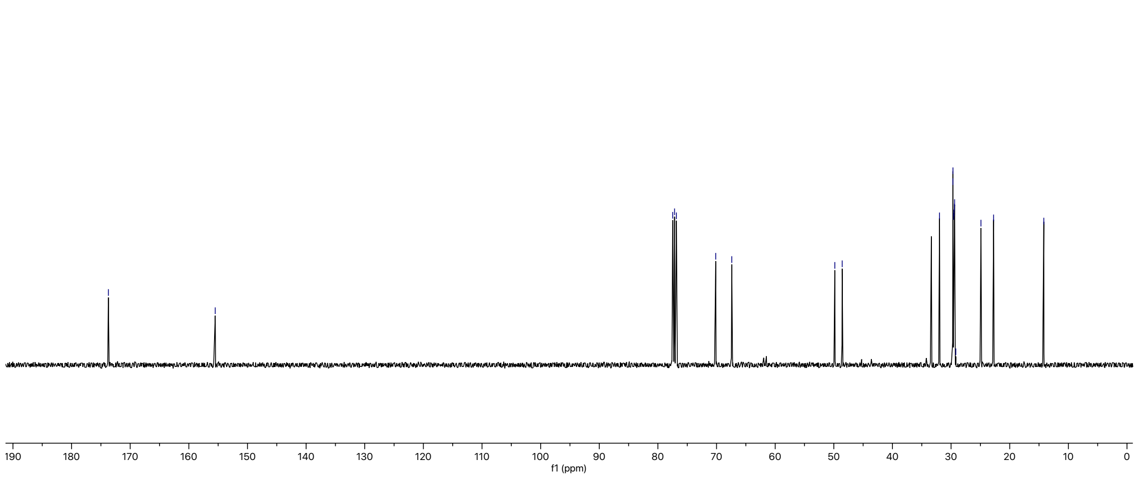

Supplementary Figure 24. NMR of bAC-2

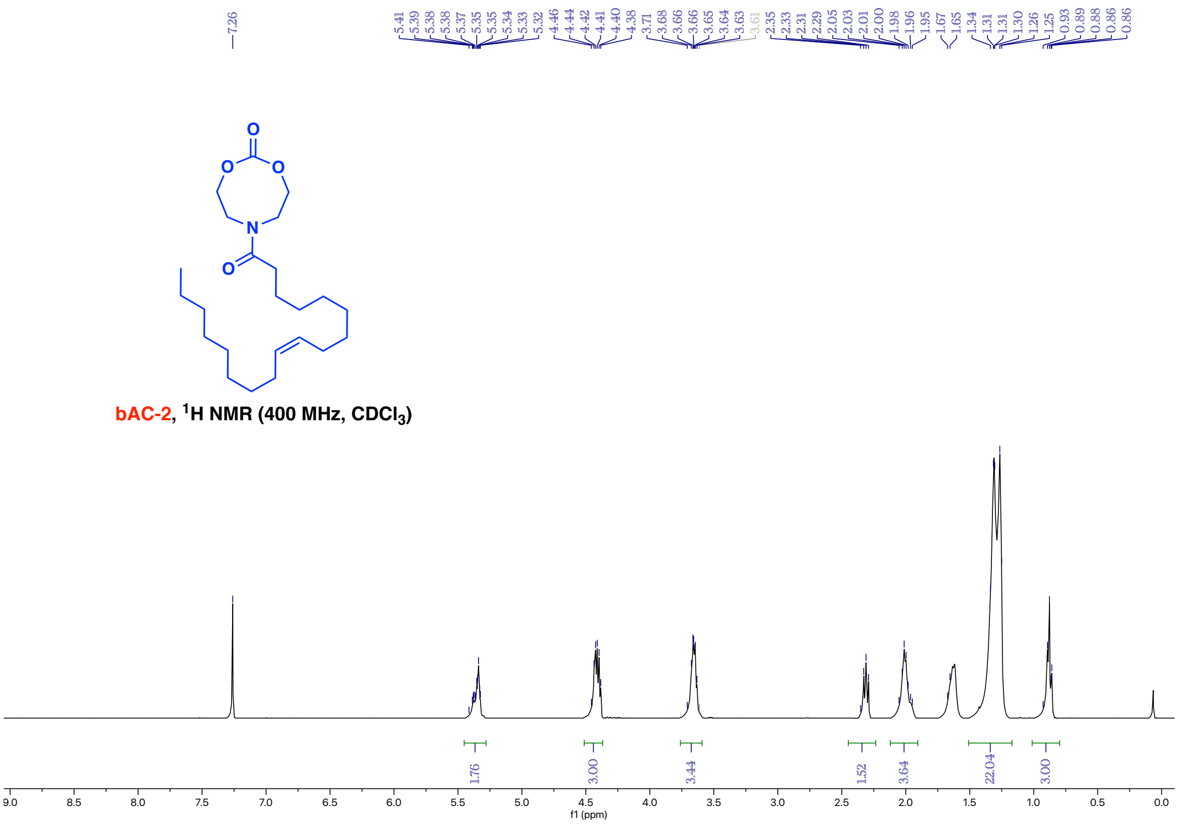

bAC-2, ^{1}H NMR (400 MHz, $CDCl_3$)

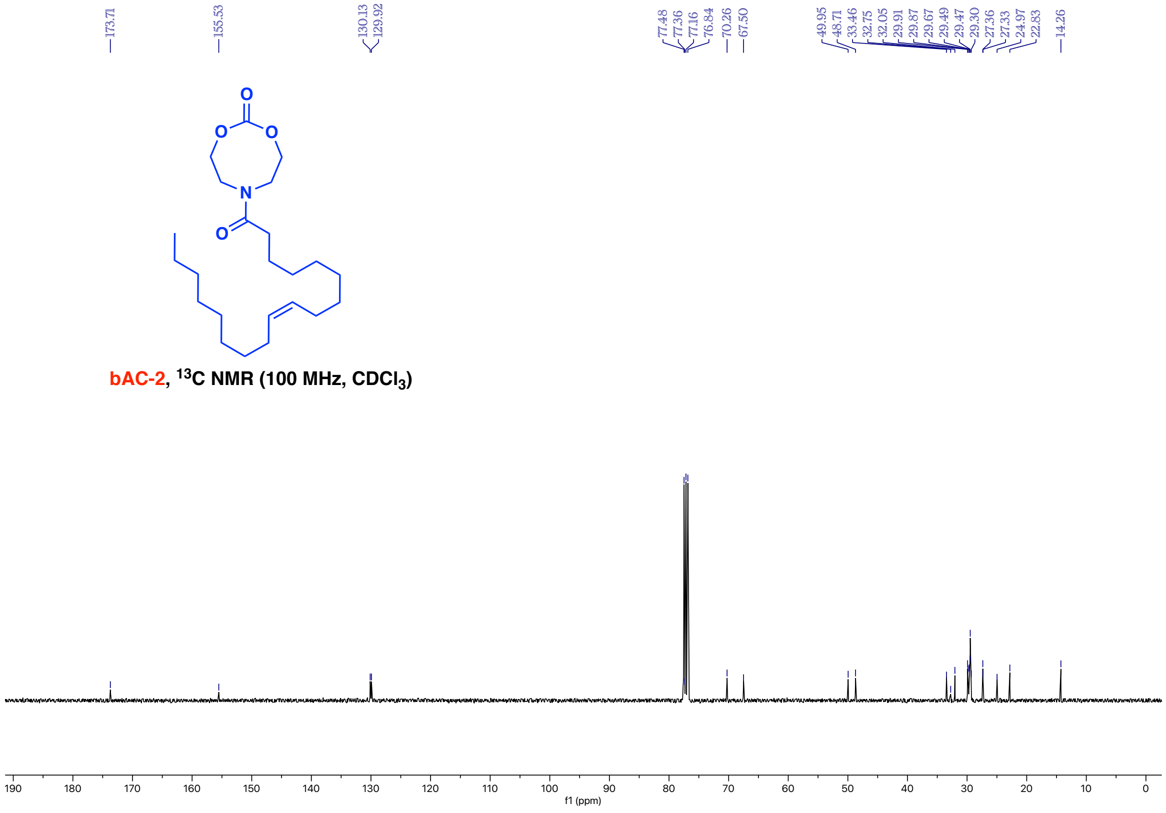

bAC-2, ^{13}C NMR (100 MHz, $CDCl_3$)

Supplementary Figure 25. NMR of bAC-3

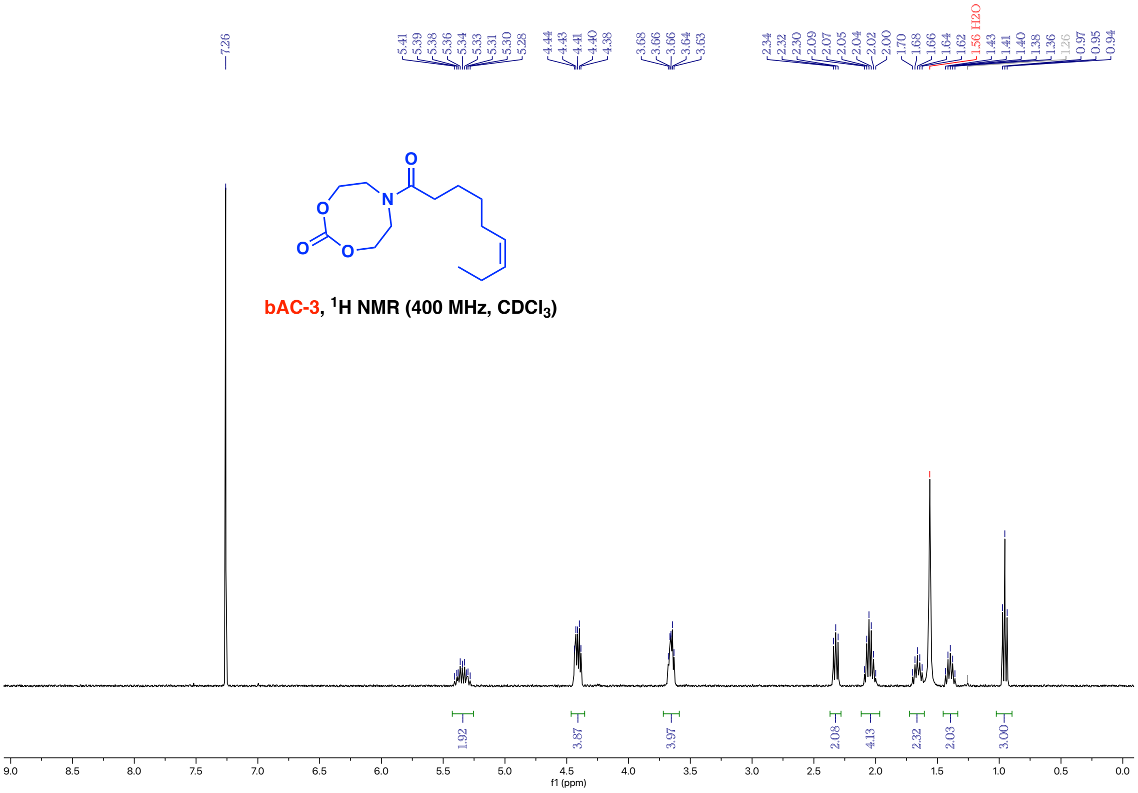

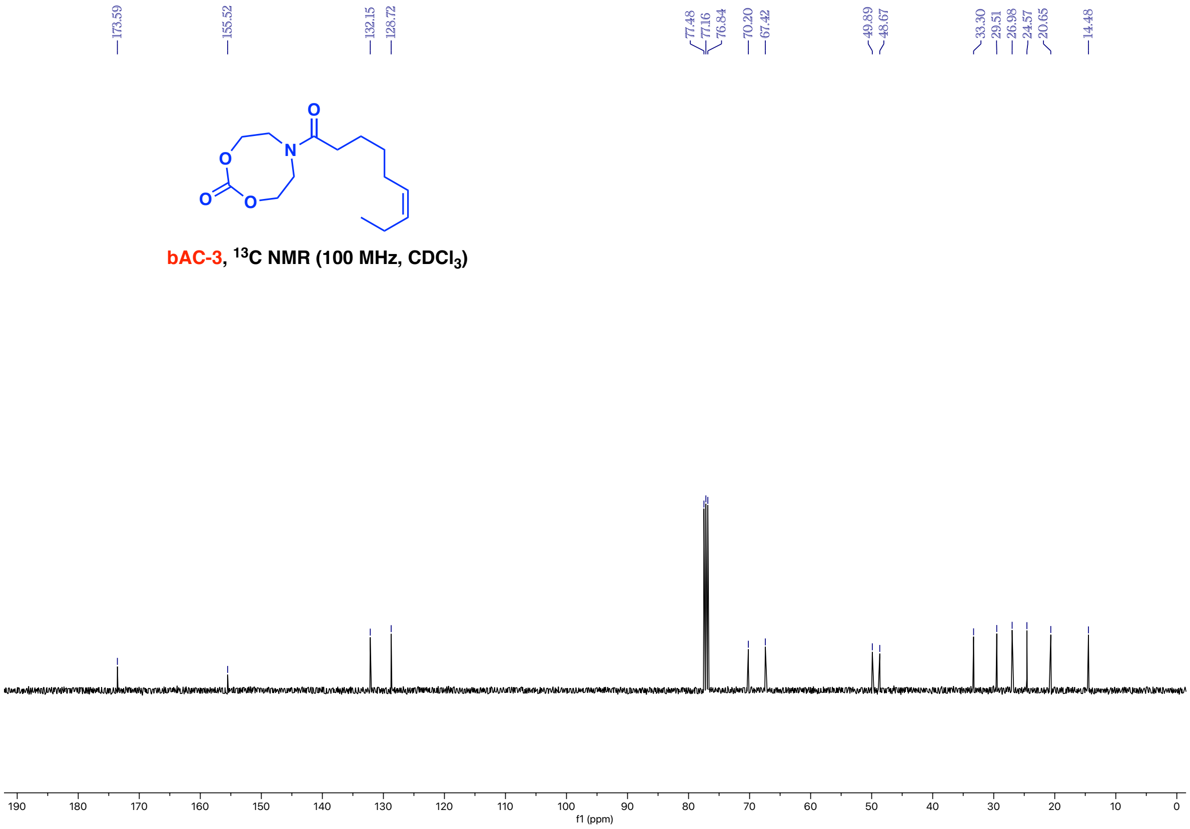

Supplementary Figure 26. NMR of bAC-4

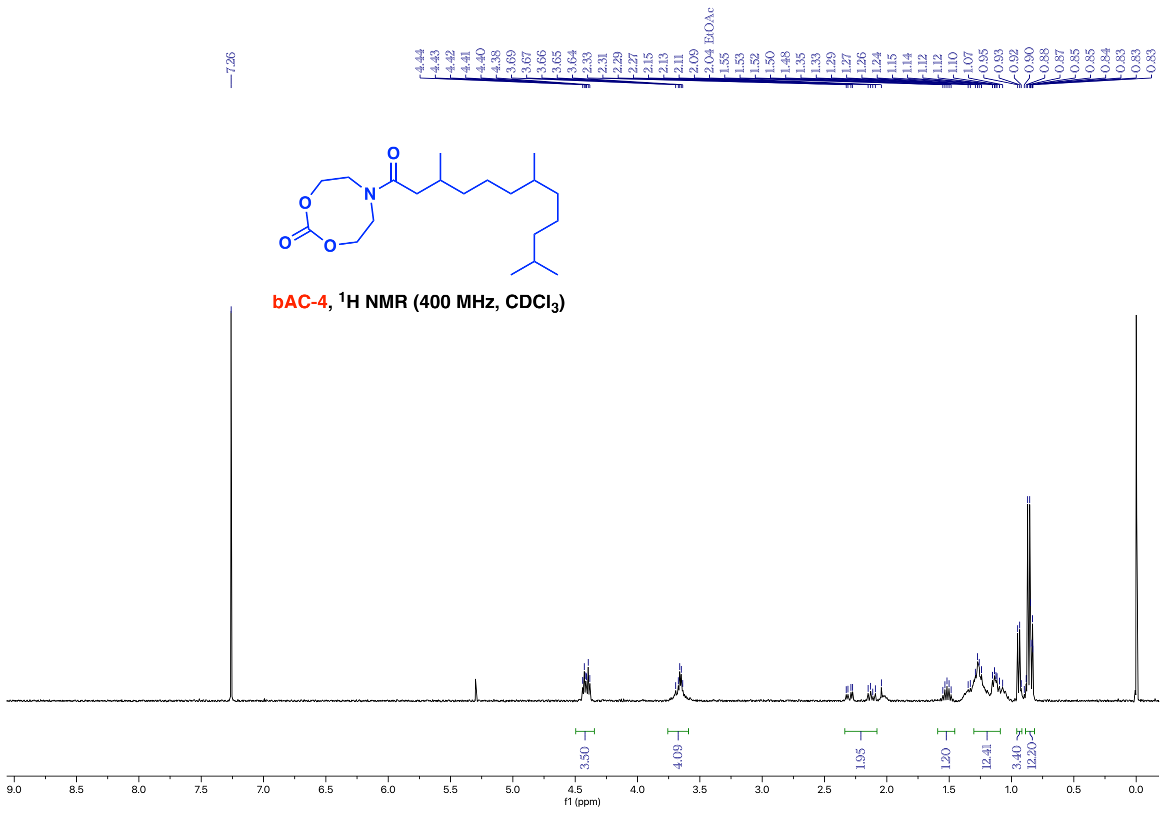

bAC-4, ^{1}H NMR (400 MHz, $CDCl_3$)

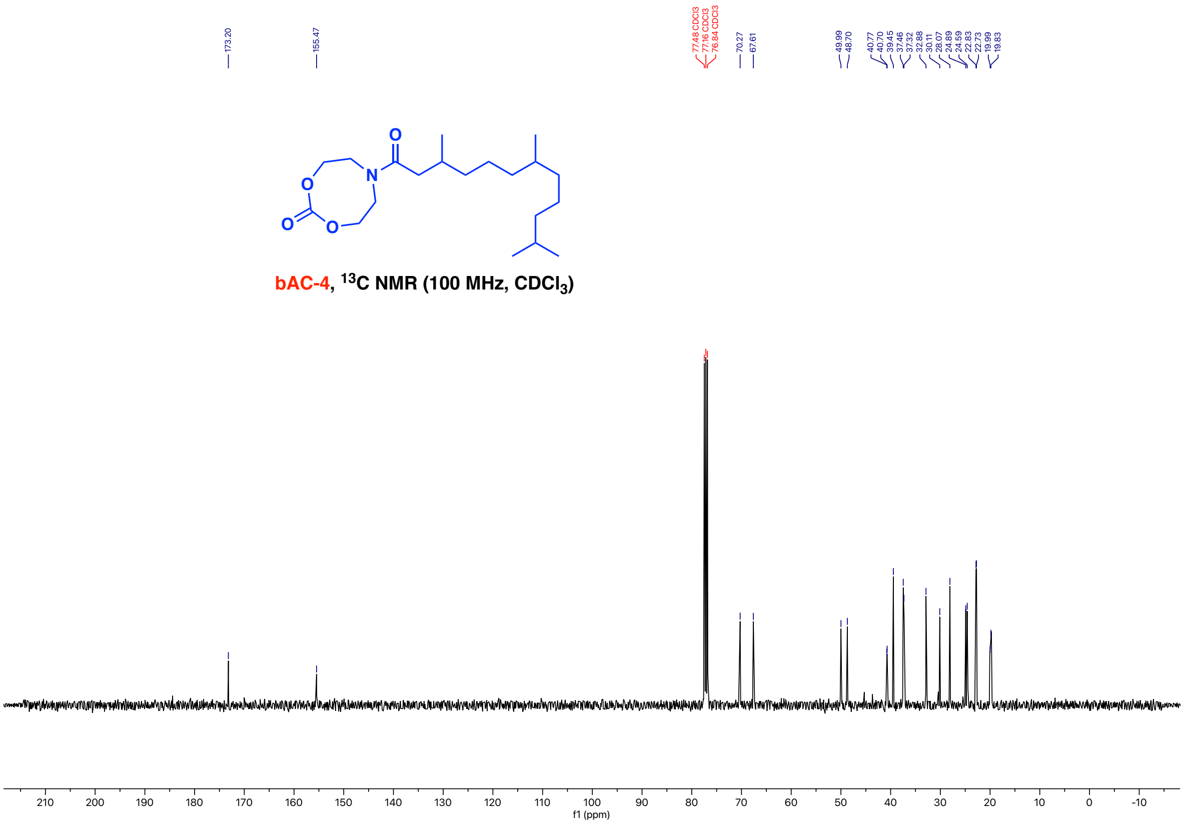

bAC-4, ^{13}C NMR (100 MHz, $CDCl_3$)

Supplementary Figure 27. NMR of bAC-5

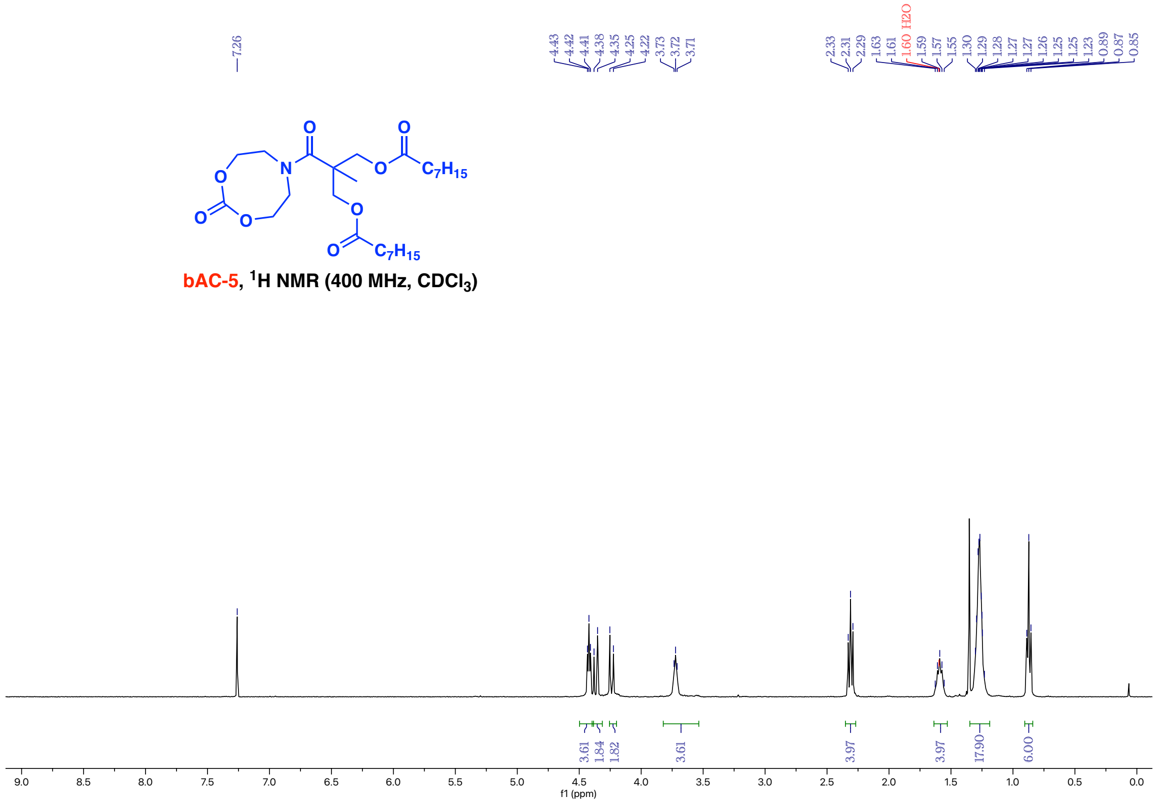

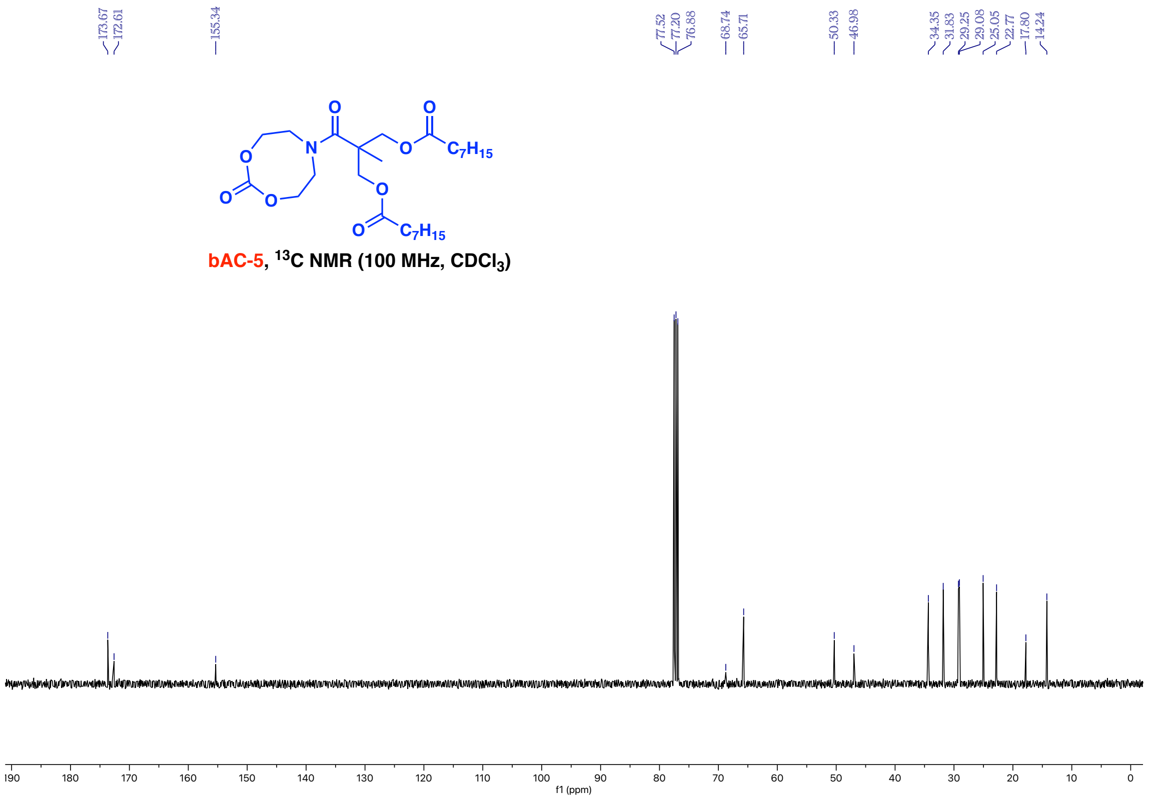

Supplementary Figure 28. NMR of bAC-6

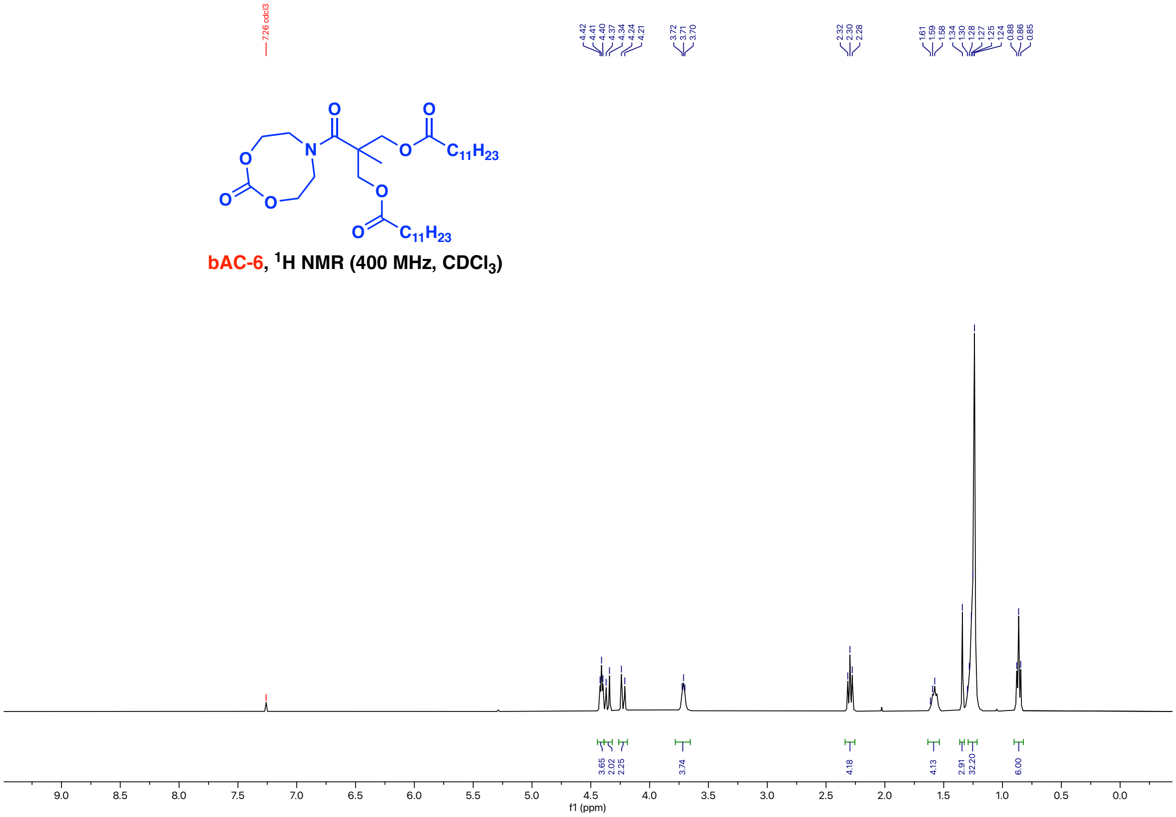

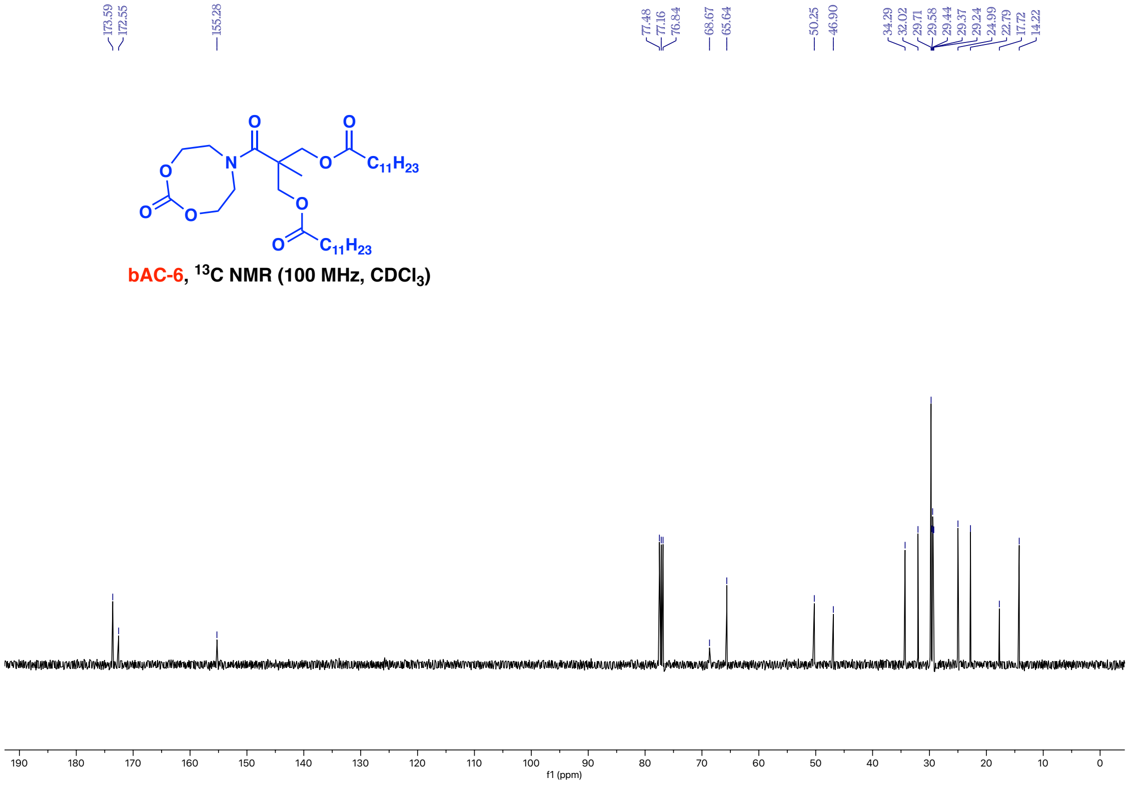

Supplementary Figure 29. NMR of bAC-7

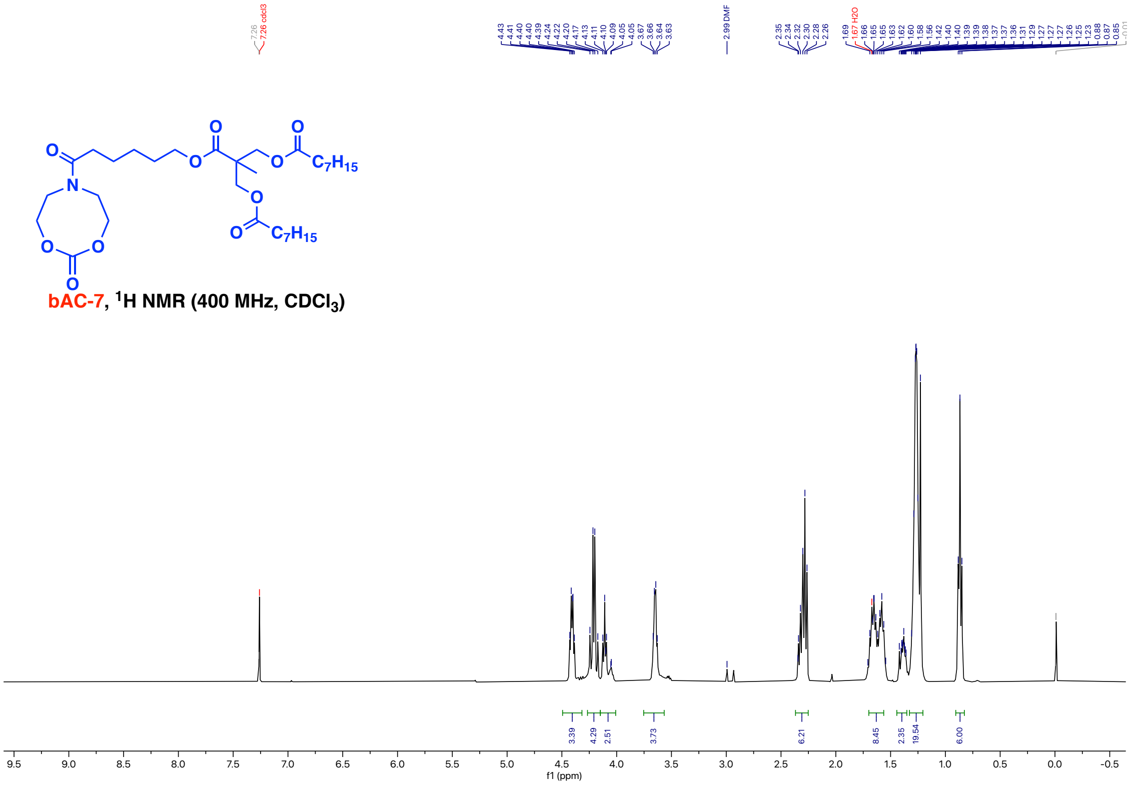

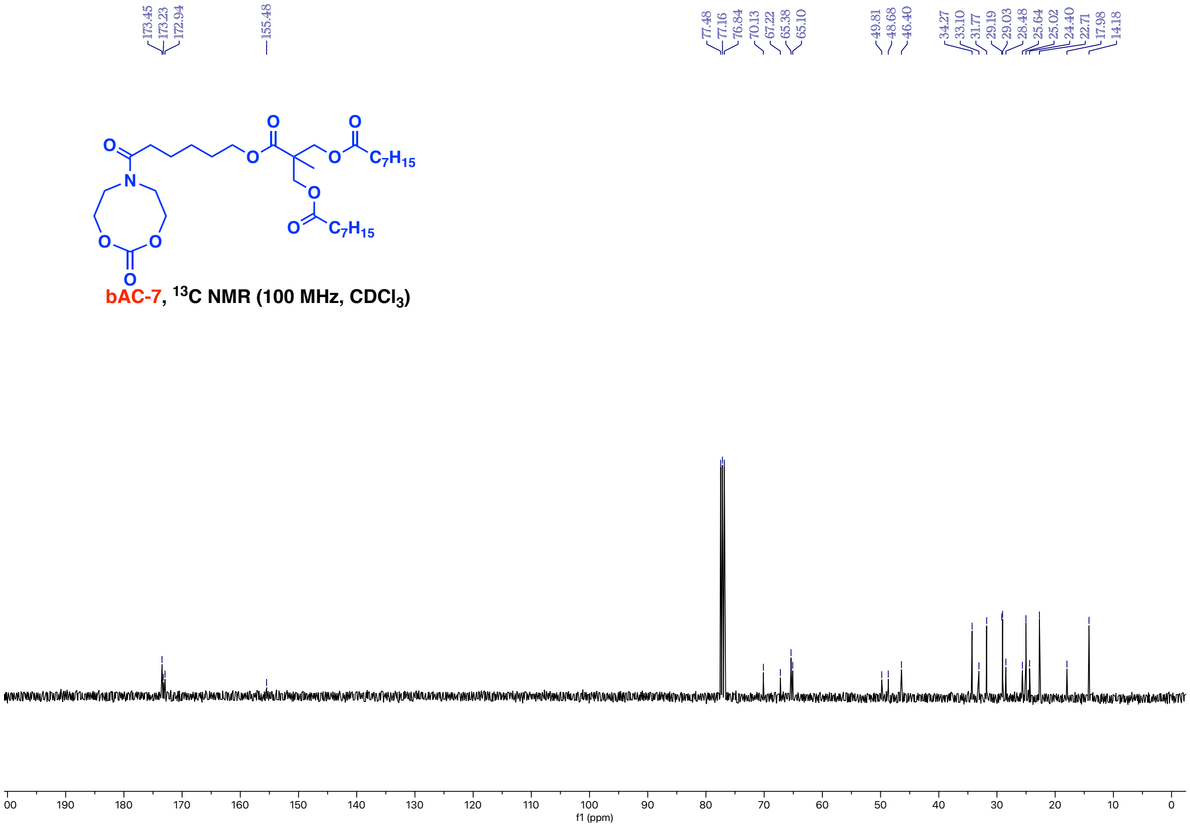

Supplementary Figure 30. NMR of bAC-8

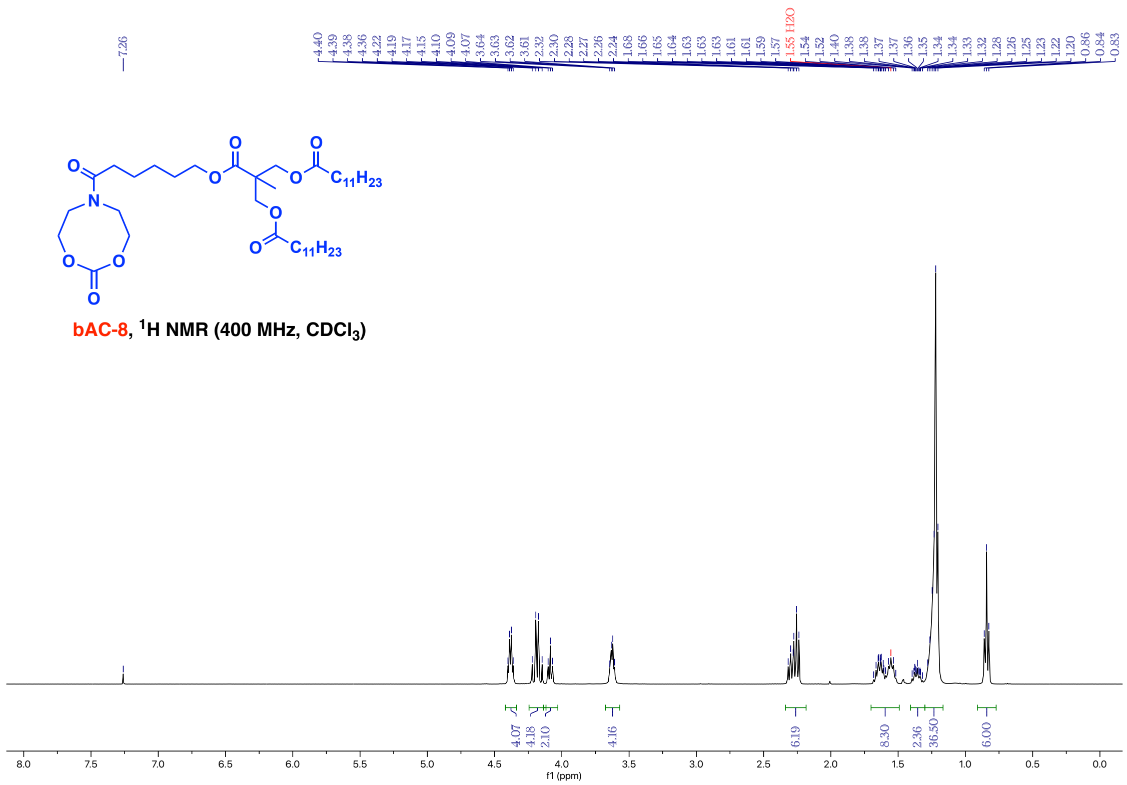

bAC-8, ^{1}H NMR (400 MHz, $CDCl_3$)

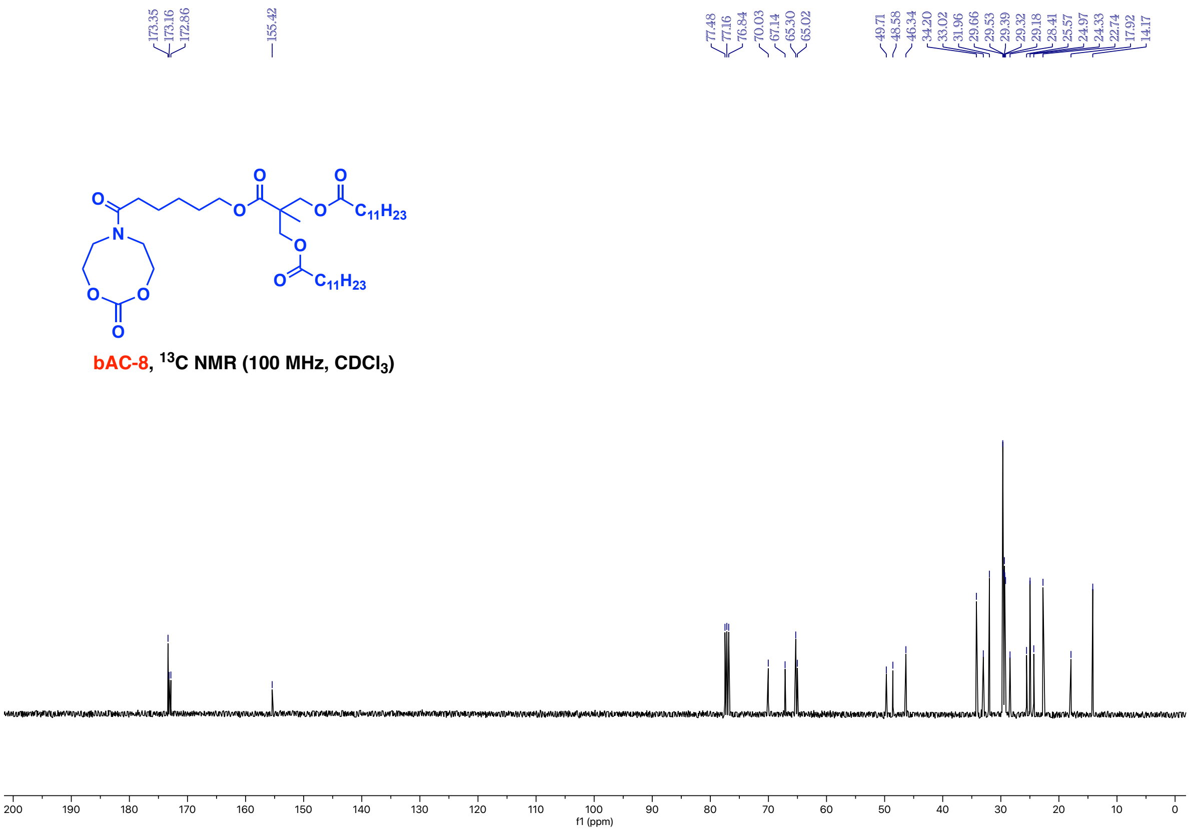

bAC-8, ^{13}C NMR (100 MHz, $CDCl_3$)

Supplementary Figure 31. NMR of MTC-7

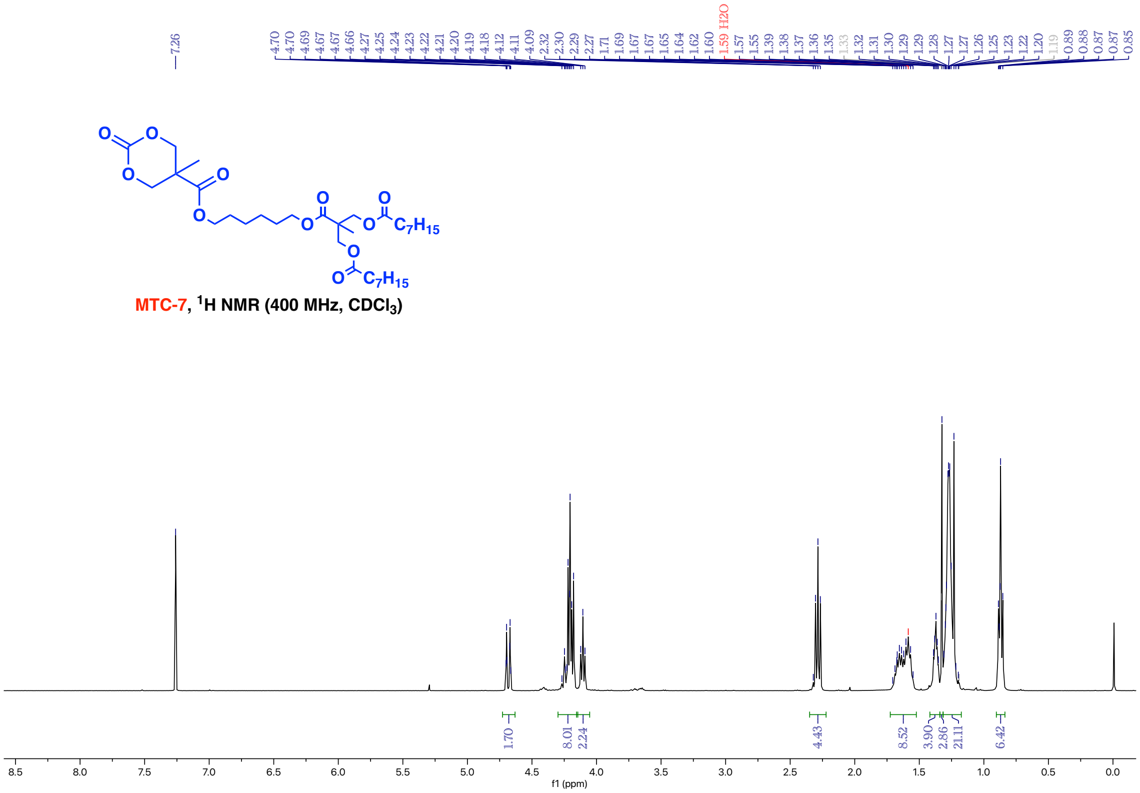

MTC-7, ^{1}H NMR (400 MHz, $CDCl_3$)

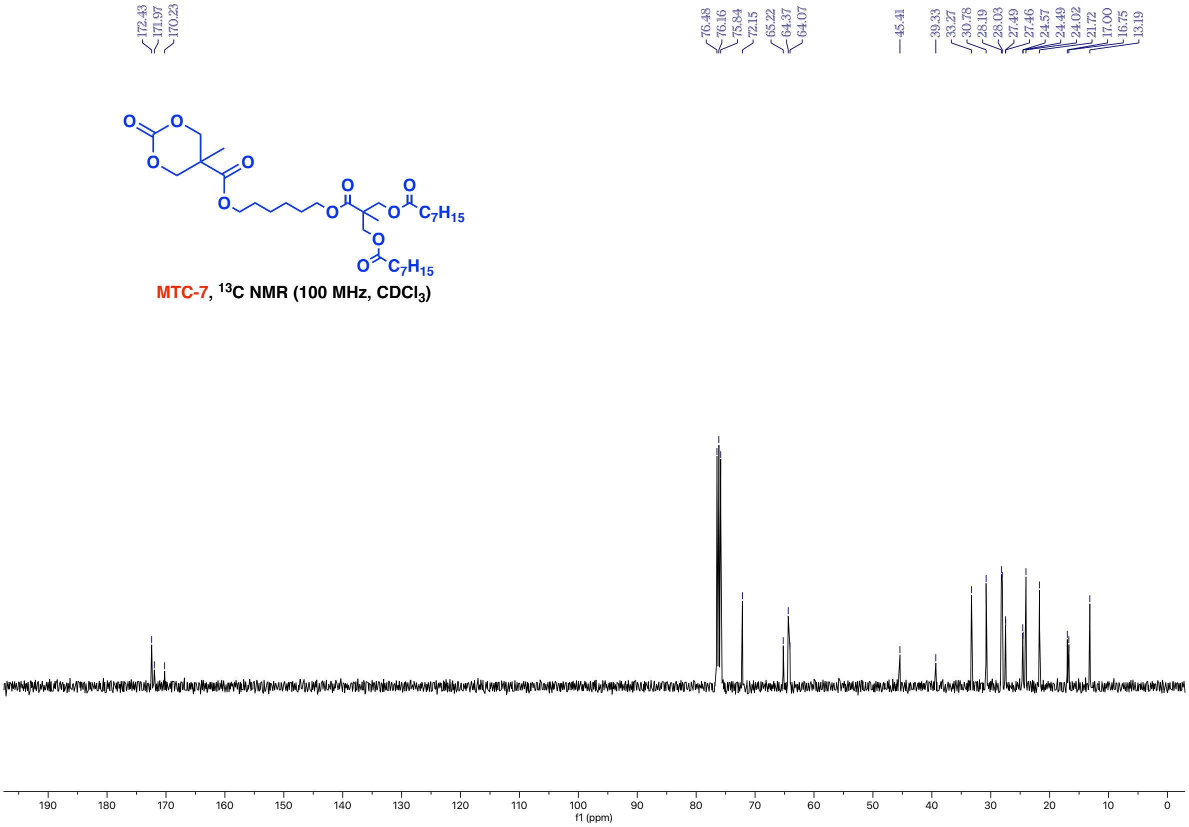

MTC-7, ^{13}C NMR (100 MHz, $CDCl_3$)

Supplementary Figure 32. NMR of MTC-8

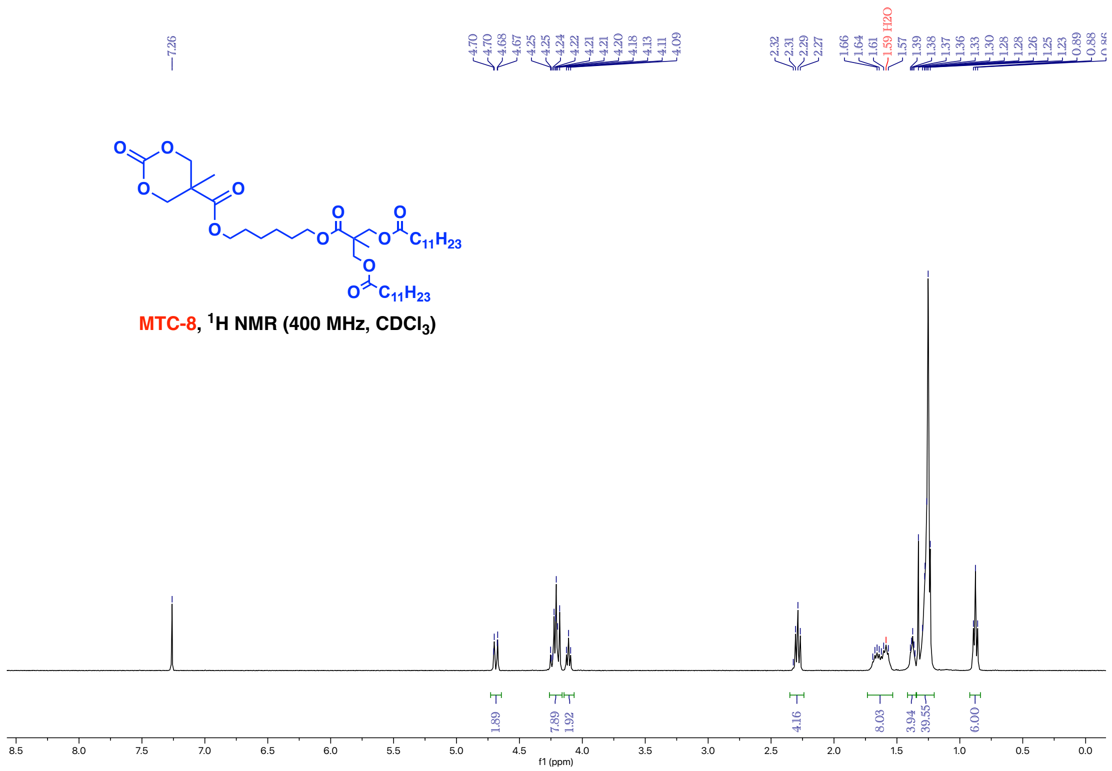

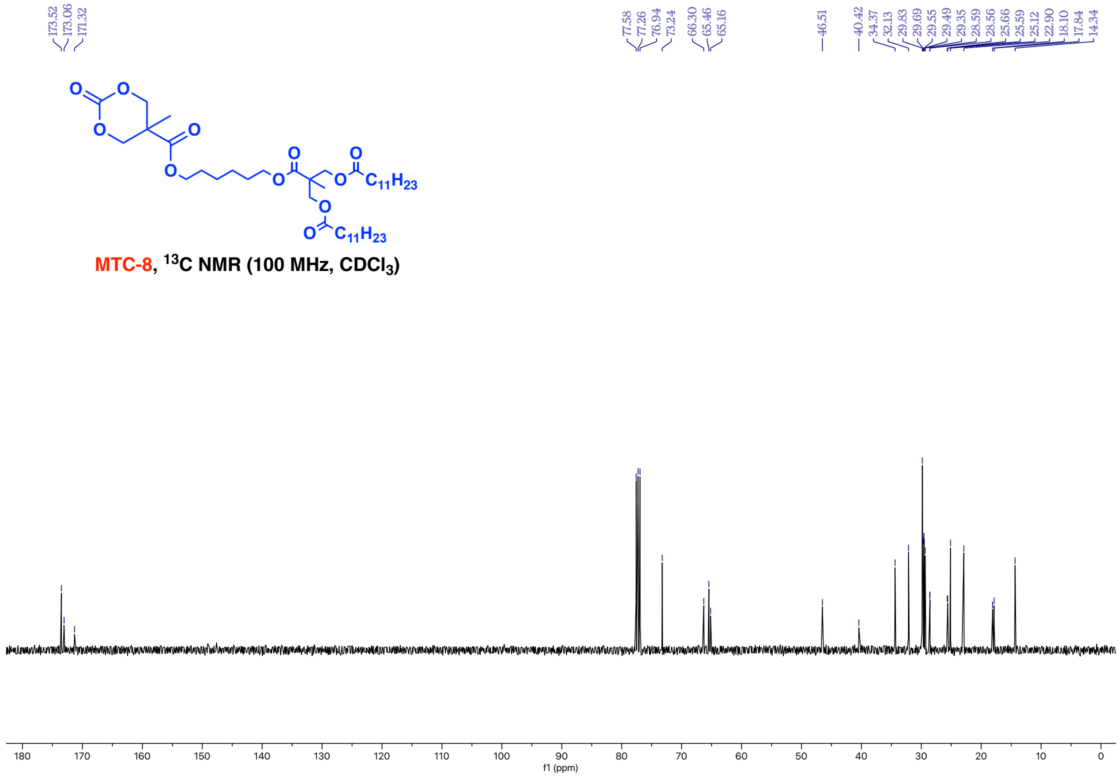

Supplementary Figure 33. NMR of bAC-1b (Boc-protected)

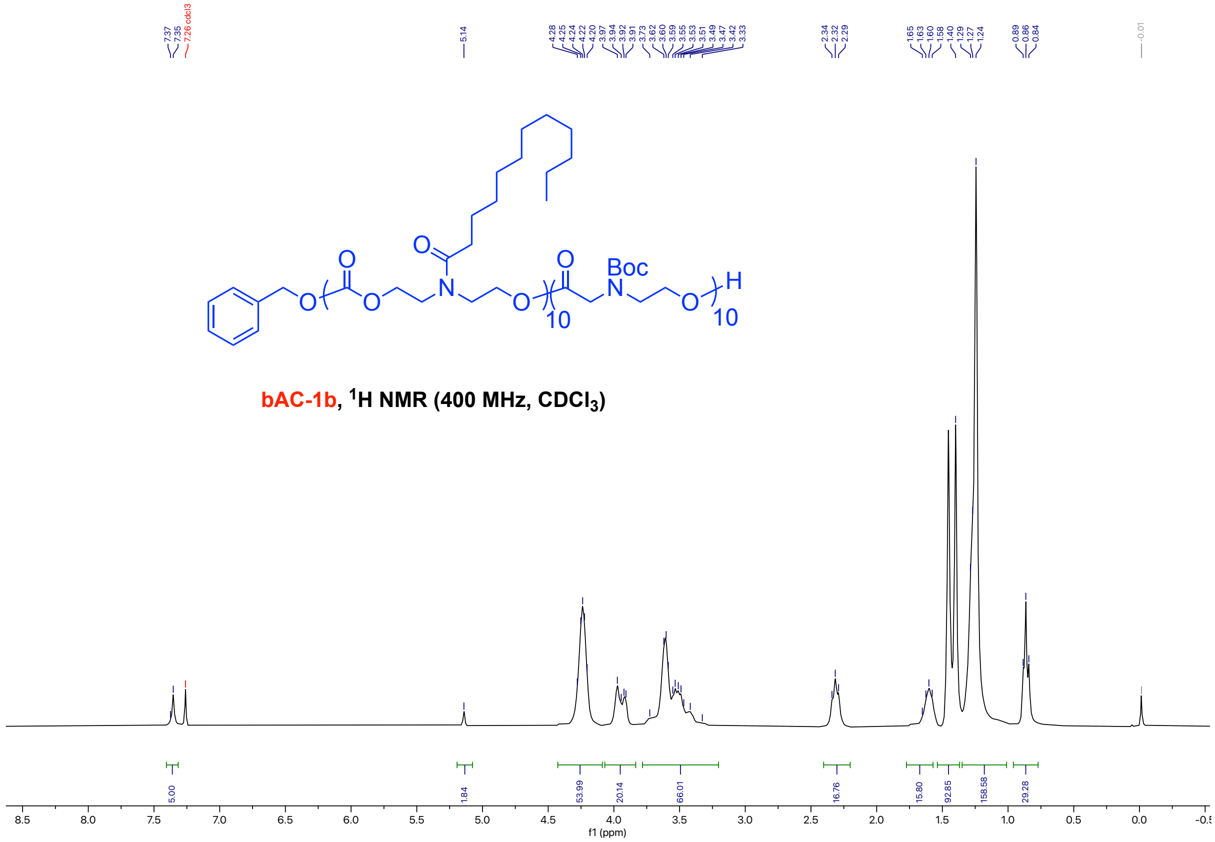

Supplementary Figure 34. NMR of bAC-2c (Boc-protected)

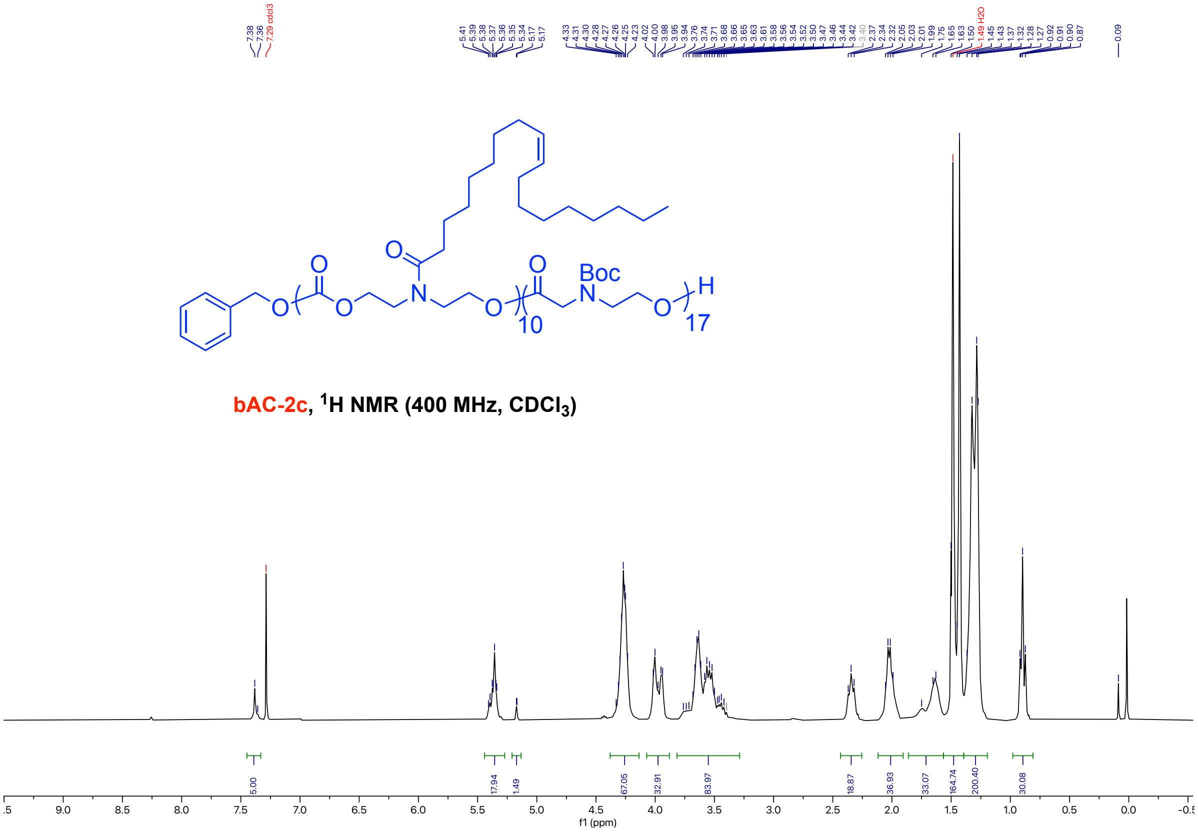

Supplementary Figure 35. NMR of bAC-3b (Boc-protected)

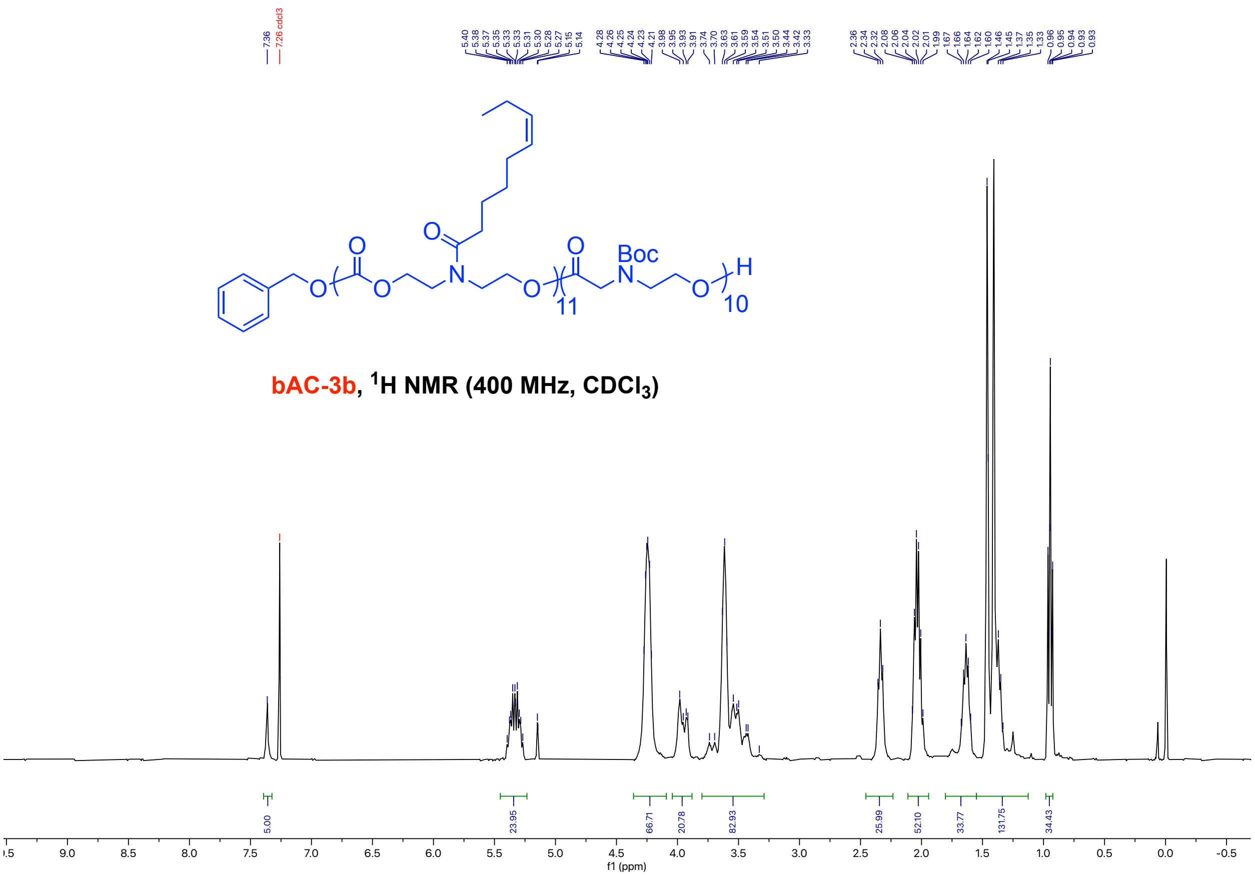

Supplementary Figure 36. NMR of bAC-4b (Boc-protected)

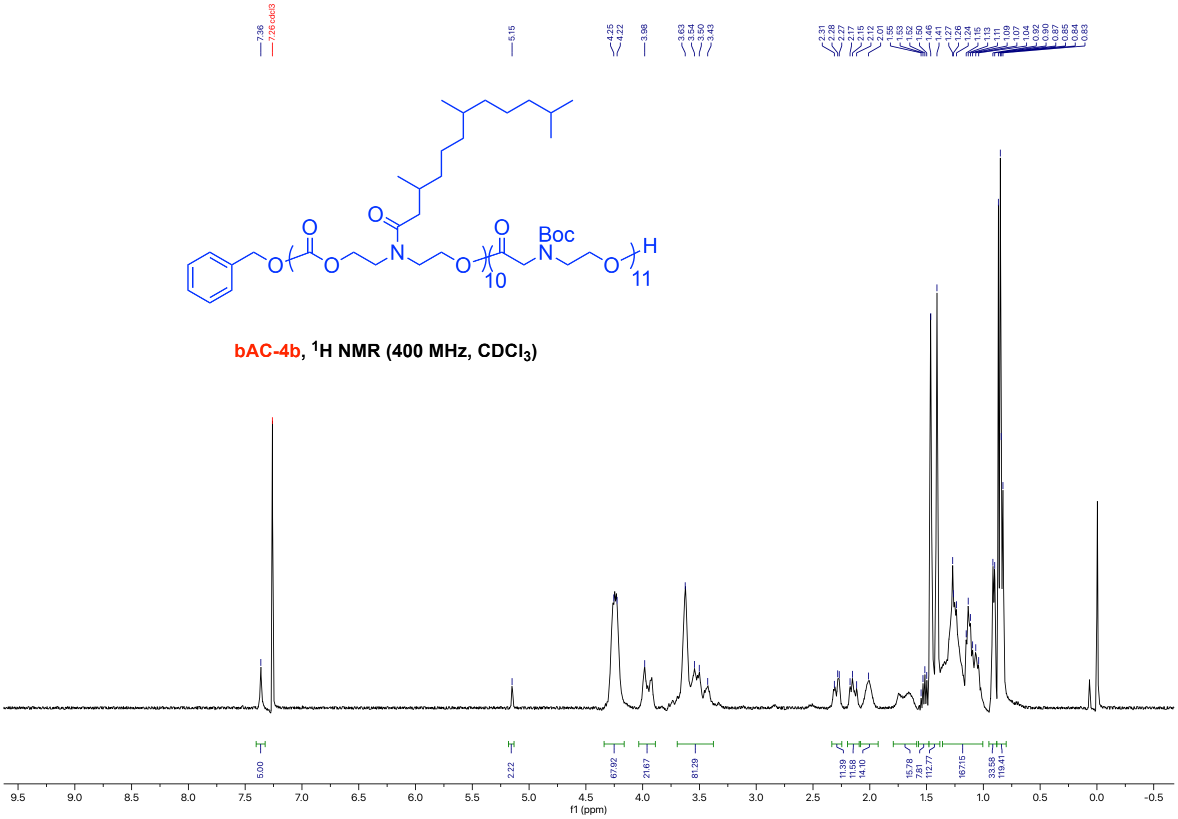

Supplementary Figure 37. NMR of bAC-5c (Boc-protected)

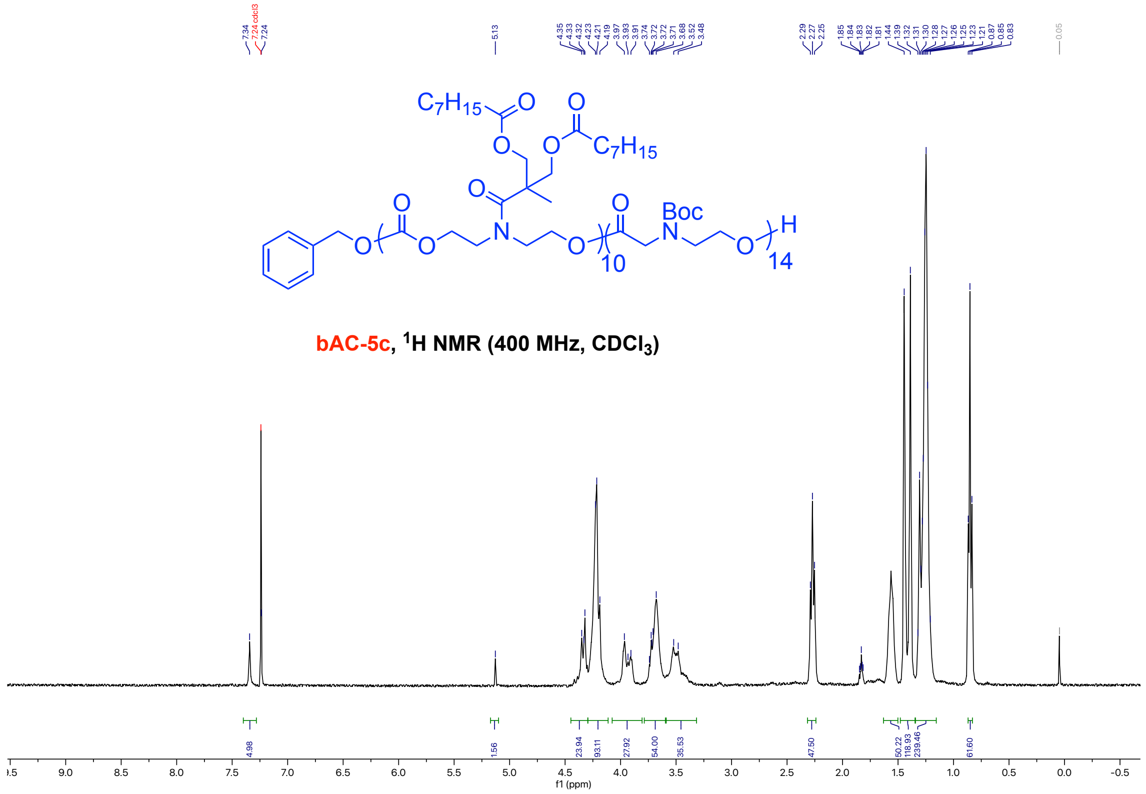

Supplementary Figure 38. NMR of bAC-6b (Boc-protected)

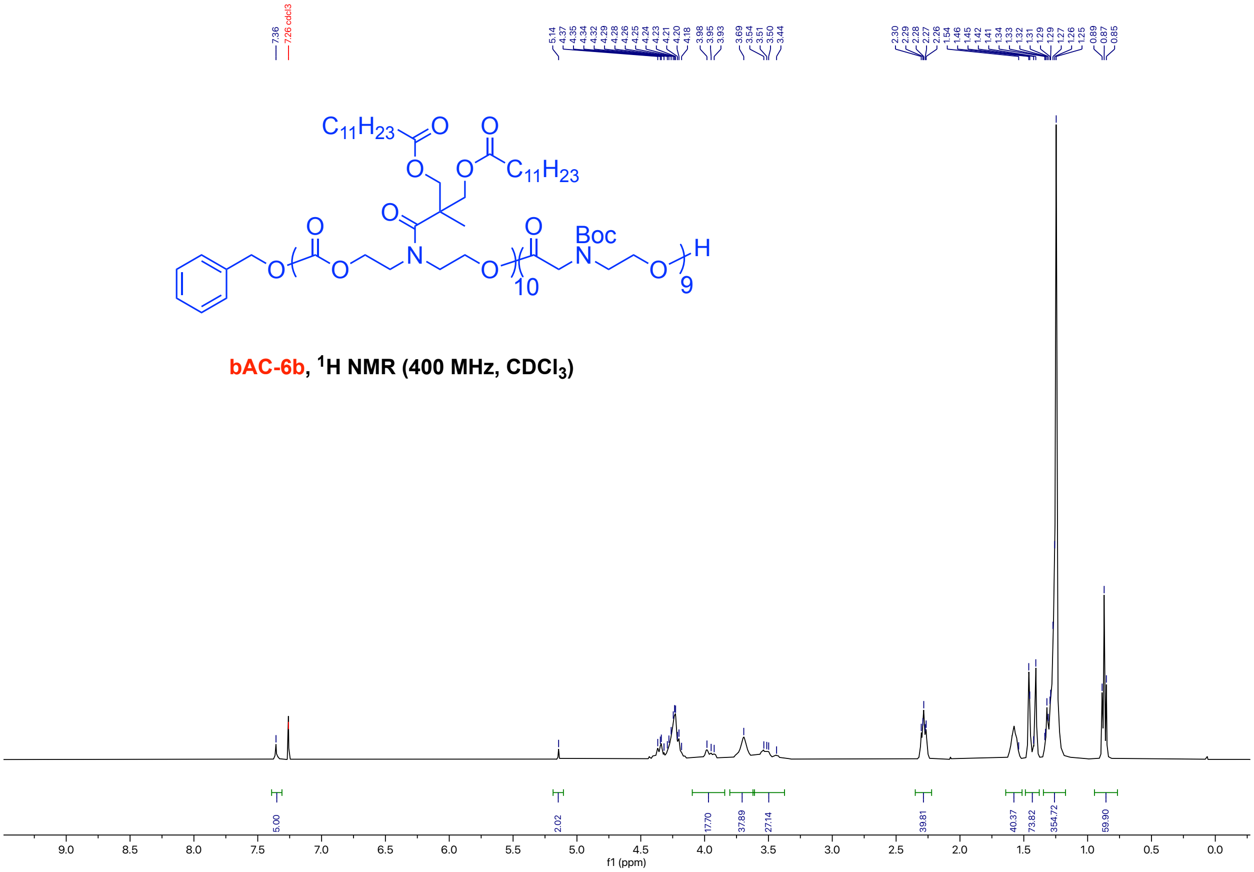

Supplementary Figure 39. NMR of bAC-7c (Boc-protected)

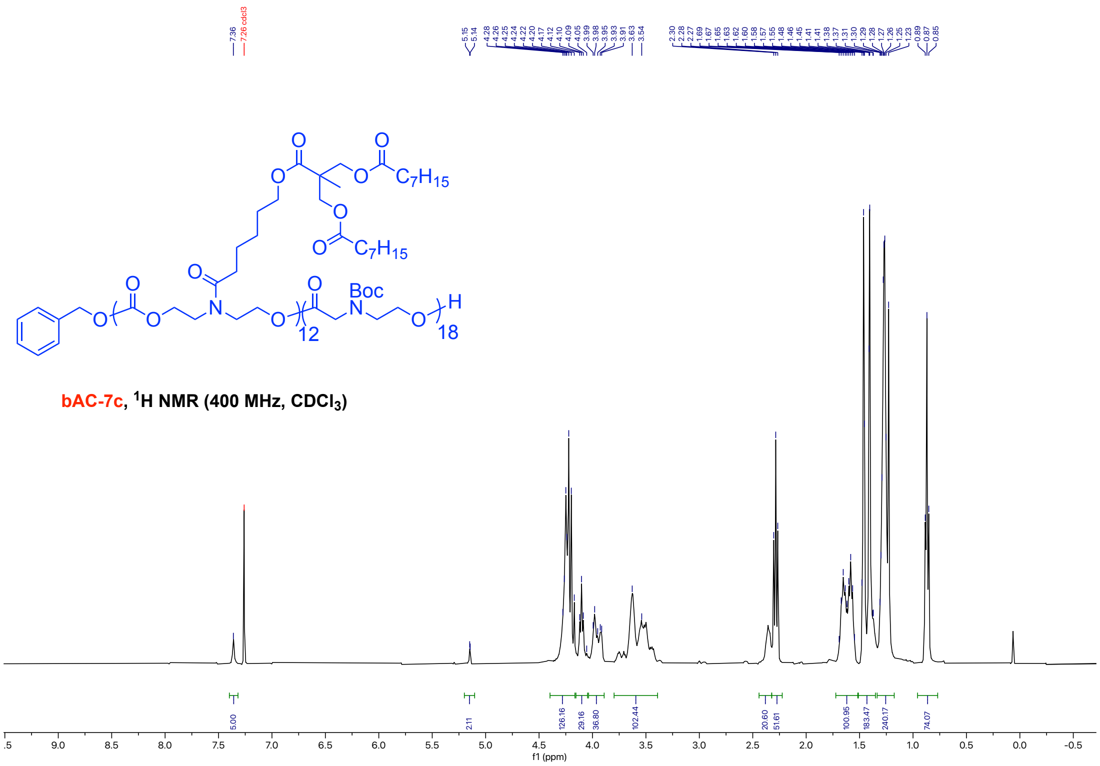

Supplementary Figure 40. NMR of bAC-8b (Boc-protected)

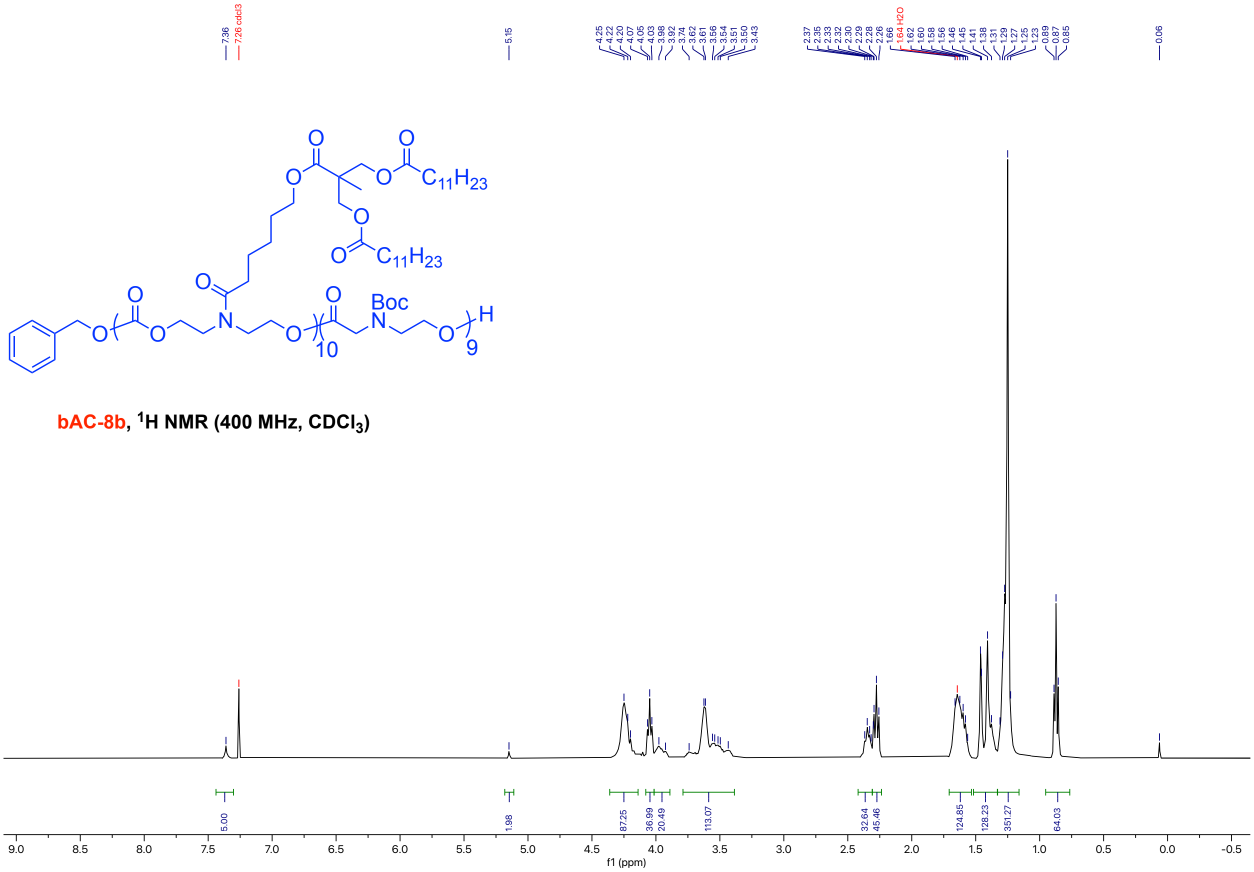

bAC-8b, ^{1}H NMR (400 MHz, $CDCl_3$)

Supplementary Methods

General Procedures

Unless otherwise noted, all reactions were conducted in oven-dried (>120 ˚C) and/or flame-dried glassware equipped with a Teflon® coated magnetic stir bar and a rubber septum under a positive pressure of nitrogen or argon or in fresh out of the box glass vials under ambient temperature and atmosphere. Tetrahydrofuran and diethyl ether were purified via passage through an activated alumina drying column (Solv-Tek, Inc.). Dichloromethane was purified via distillation from calcium hydride or by passage through an activated alumina drying column (Solv-Tek, Inc.). Toluene was purified by passage through an activated alumina drying column (Solv-Tek, Inc.). Pyridine and triethylamine were distilled from calcium hydride under a positive pressure of nitrogen. Deuterated chloroform was dried and de-acidified by storing over activated 4Å molecular sieves and solid potassium carbonate.

Analytical thin-layer chromatography (TLC) was performed by using glass-backed silica plates coated with a 0.25 mm thickness of silica gel 60 F254 (EDM Millipore), visualized with an ultraviolet light, followed by exposure to *p*-anisaldehyde solution, potassium permanganate solution, or ceric ammonium molybdate solution and gentle heating. Products were purified by flash-chromatography on (230-400 mesh, grade 60, particle size 40 to 63 μm) purchased from Fisher Scientific or by preparative TLC with glass-backed silica plates coated with a 0.25 mm thickness of silica gel 60 F254 (EMD Millipore).

Proton NMR spectra were recorded in $CDCl_3$ on a Varian 400 (400 MHz), and/or Varian Inova 600 (600 MHz). Carbon-13 NMR spectra were recorded on a Varian 400 (100 MHz). The following format is used to report the proton NMR data: chemical shift in ppm [multiplicity, coupling constant(s) in Hz, integral, and assignment]. Chemical shifts for proton spectra are referenced to TMS (δ 0.00 ppm) or residual solvent peak (δ 7.26 ppm for chloroform). First order multiplicity is described as s (singlet), d (doublet), t (triplet), q (quartet), or combination thereof. Chemical shifts for ^{13}C NMR spectra in $CDCl_3$ are referenced to the carbon resonance in $CDCl_3$ (δ 77.16 ppm).

High-resolution mass spectra (HRMS) were acquired at the Vincent Coates Foundation Mass Spectrometry Laboratory at Stanford University with Exploris 240 BioPharma. Unless otherwise noted, all reagents were obtained from commercial sources and used without additional purification.

Reagent list

Chemical Reagents	Vendor	Abbreviation
5-Nonen-1-ol	Sigma-Aldrich	NA
5-methyl-2-oxo-1,3-dioxane-5-carboxylic acid	Sigma-Aldrich	BIS-MPA
Lauroyl chloride	Sigma-Aldrich	NA
Octanoyl chloride	Sigma-Aldrich	NA
Farnesol	Sigma-Aldrich	NA
1-Ethyl-3-(3-dimethylaminopropyl)carbodiimide	Oakwood	EDCI
4-Dimethylaminopyridine	Sigma-Aldrich	DMAP
Anhydrous magnesium sulphate	Fisher Scientific	$MgSO_4$
Sulfuric acid	Fisher Scientific	H_2SO_4
Oxalyl chloride	Oakwood	$(COCl)_2$
1,6-Hexanediol	Sigma-Aldrich	NA
Chromium (IV) oxide	Sigma-Aldrich	CrO_3
Triphosgene	Oakwood	$C_3Cl_6O_3$

Synthetic procedures and chemical characterization data

A. Synthesis of bAC monomers (bAC 1-8)

A1. Synthesis of intermediates

Synthesis of **Lipidic acid 3**

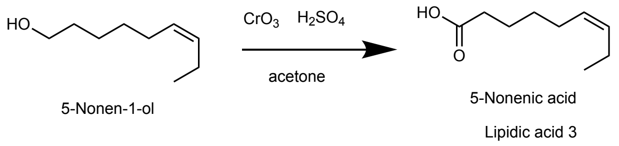

5-Nonen-1-ol (1.00 g, 7.03 mmol, 1 equiv) was dissolved in 45.0 ml acetone and chilled in an ice bath. The Jones reagent was prepared by first dissolving CrO_3 (1.76 g, 17.58 mmol, 2.2 equiv) in 3.50 ml water after which H_2SO_4 (2.56 g, 26.01 mmol, 3.7 equiv) was added dropwise with stirring over 1 min in an ice bath. The Jones reagent was then added dropwise to the alcohol solution over 15 min. After addition, the reaction was allowed to warm up to room temperature (25°C) and after 16 h, it was subsequently quenched with 2.00 ml of isopropanol. The solvent was evaporated and the crude mixture was resuspended in 20.0 ml hexane. The organic layer was then washed with 20.0 ml water, brine, and dried over $MgSO_4$, then the solvent was removed *in vacuo* to provide the crude product as colorless oil. The residue was purified with silica gel column chromatography with 25% EtOAc/Hexane, 1% AcOH as eluent to afford **Lipidic acid 3** as colorless oil (746 mg, 4.78 mmol, 68% yield).

^{1}H NMR (400 MHz, $CDCl_3$) δ 5.44 – 5.26 (m, 2H), 2.36 (t, J = 7.5 Hz, 2H), 2.12 – 1.97 (m, 4H), 1.73 – 1.59 (m, 2H), 1.48 – 1.34 (m, 2H), 0.95 (t, J = 7.6 Hz, 3H).

^{13}C NMR (100 MHz, $CDCl_3$) δ 179.57, 132.32, 128.55, 33.98, 29.24, 26.80, 24.41, 20.67, 14.49.

HRMS (ESI-TOF) m/z: $[M+H]^+$ Calculated for $C_9H_{16}O_2$: 157.1223; found 157.1220.

Synthesis of **Lipidic acid 4**

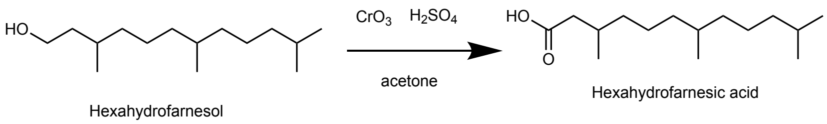

Lipidic acid 4

Lipidic acid 4 was formed from hexahydrofarnesol following a protocol similar to that used for **lipidic acid 3** formation. Hexahydrofarnesol (1.00g, 4.38 mmol, 1 equiv), CrO_3 (1.09 g, 10.94 mmol, 2.2 equiv), H_2SO_4 (1.59 g, 16.20 mmol, 3.7 equiv), and 30 ml acetone were subjected to the reaction condition described above. The residue was purified with silica gel flash column chromatography with 25% EtOAc/Hexane, 1% AcOH as eluent to afford **Lipidic acid 4** as a colorless oil (739 mg, 3.02 mmol, 69% yield). ^{1}H NMR, ^{13}C NMR spectra agreed with the previous literature[2].

^{1}H NMR (400 MHz, $CDCl_3$) δ 2.44 – 2.12 (m, 2H), 2.07 – 1.88 (m, 1H), 1.54 (dq, *J* = 13.2, 6.7 Hz, 1H), 1.47 – 1.04 (m, 13H), 1.00 (d, *J* = 6.6 Hz, 3H), 0.88 (t, *J* = 6.6 Hz, 9H).

Synthesis of **Lipidic acid 5**

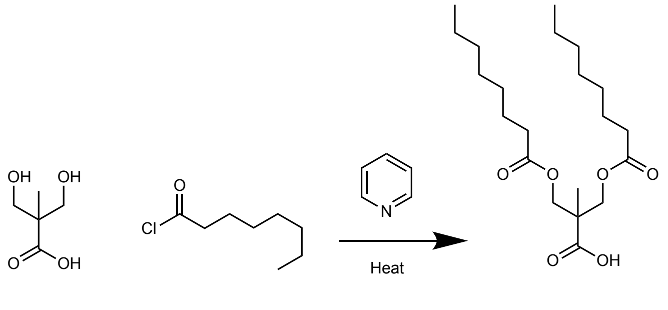

Bis-MPA (3.00 g, 22.37 mmol, 1 equiv) was added to 10.0 ml pyridine and heated at 60°C until all solids dissolved. Octanoyl chloride (8.00 g, 49.21 mmol, 2.2 equiv) was added in one portion and the suspension was heated at 60°C for 24 h. After completion of the reaction, 25.0 ml EtOAc was added to the reaction mixture and the resulting mixture was washed with 30.0 ml aqueous 1 N HCl, brine and dried over $MgSO_4$. The solvent was removed *in vacuo* to provide the crude product as a slightly yellow oil. Purification was accomplished by silica gel flash column chromatography (10-20% EtOAc/hexane, 1% AcOH as eluent) affording **Lipidic acid 5** (6.05 g, 15.66 mmol, 70% yield) as a slightly yellow oil. Compound purity was established by TLC (one spot) analysis.

^{1}H NMR (400 MHz, $CDCl_3$) *δ* 4.30 – 4.19 (m, 4H), 2.31 (t, *J* = 7.5 Hz, 4H), 1.62 (m, 4H), 1.27 (d, *J* = 10.5 Hz, 19H), 0.92 – 0.84 (m, 6H).

^{13}C NMR (100 MHz, $CDCl_3$) *δ* 179.34, 173.96, 65.53, 46.68, 34.66, 32.20, 29.60, 29.45, 25.41, 23.14, 18.33, 14.60.

HRMS (ESI-TOF) m/z: $[M-H]^-$ Calculated for $C_{21}H_{38}O_6$: 385.2596; found 385.2593.

Synthesis of **Lipidic acid 6**

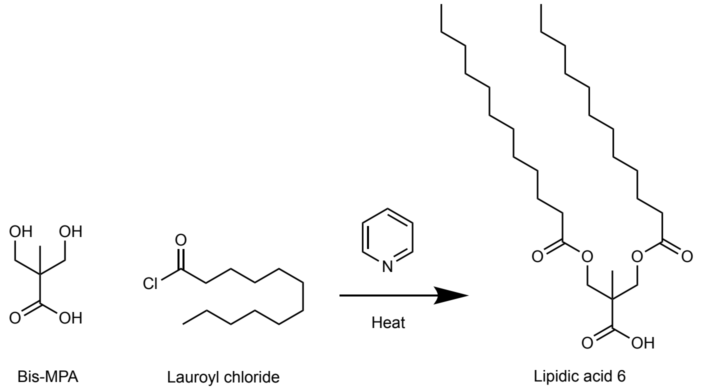

Lipidic acid 6 was formed from Bis-MPA following a protocol similar as **lipidic acid 5** formation. Bis-MPA (3.00 g, 22.37 mmol, 1 equiv), lauoryl chloride (10.76 g, 49.21 mmol, 2.2 equiv), and 10.0 ml pyridine were subjected to the reaction condition detailed above. The residue was re-crystalized in methanol at -20°C for 24 h to yield pure product as a white solid (9.49 g, 19.01 mmol, 85% yield). Compound purity was established by TLC (one spot) analysis.

^{1}H NMR (400 MHz, $CDCl_3$) *δ* 4.33 – 4.18 (m, 4H), 2.30 (t, *J* = 7.6 Hz, 4H), 1.59 (t, *J* = 7.2 Hz, 4H), 1.44 – 1.21 (m, 35H), 0.87 (t, *J* = 6.7 Hz, 6H).

^{13}C NMR (100 MHz, $CDCl_3$) *δ* 179.42, 173.92, 65.53, 46.70, 34.69, 34.60, 32.49, 30.19, 30.05, 30.01, 29.92, 29.83, 29.69, 29.64, 25.44, 25.21, 23.26, 18.35, 14.69.

HRMS (ESI-TOF) m/z: $[M+H]^+$ Calculated for $C_{29}H_{54}O_6$: 499.3993; found 499.3983.

Synthesis of **Lipid alcohol 7**

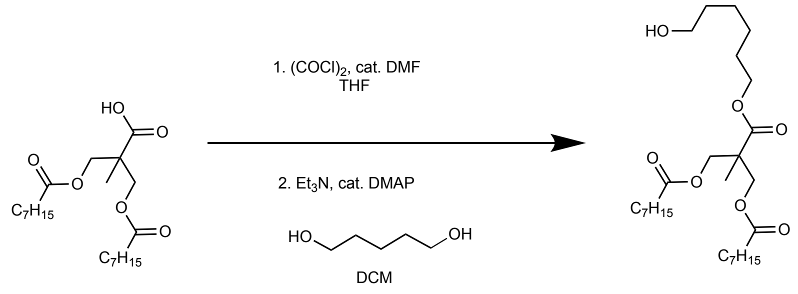

Lipidic acid 5 Lipidic alcohol 7

Lipidic acid 5 (1.20 g, 3.1 mmol,1 equiv) was dissolved in 10.0 ml dry THF and 10 drops of DMF were added. The solution was chilled on ice for 5 min and oxalyl chloride (293 ul, 3.42 mmol, 1.1 equiv) was added dropwise with a syringe over 5 min. The reaction was allowed to warm to room temperature (25°C) and stirred for 1hr. The solvent was then evaporated to yield the crude acid chloride. The crude intermediate was dried under high vacuum for additional 2 h, and then redissolved in 6.0 ml dry DCM. In a separate vessel, 1,6-hexanediol (1.10 g, 9.3 mmol, 3 equiv) was resuspended in 20.0 ml dry DCM along with DMAP (152 mg, 1.24 mmol, 0.4 equiv) and triethylamine (1.29 ml, 9.31 mmol, 3 equiv) until all the solid dissolved. The acid chloride solution was added dropwise to the alcohol solution at room temperature (25°C) and reaction proceeded for 16 h. The reaction was washed with 20 ml aqueous 1 N HCl and then brine, and dried with $MgSO_4$. The solvent was evaporated *in vacuo* to produce a slightly yellow oil as the crude product. The residue was purified by silica gel flash column chromatography with 30% EtOAc/Hexane as eluent to afford **Lipidic alcohol 7** as a colorless oil (1.22 g, 2.51 mmol, 81% yield). Compound purity was established by TLC (one spot) analysis.

^{1}H NMR (400 MHz, $CDCl_3$) δ 4.27 – 4.14 (m, 4H), 4.09 (t, J = 6.6 Hz, 2H), 3.61 (t, J = 6.5 Hz, 2H), 2.27 (t, J = 7.6 Hz, 4H), 1.68 – 1.54 (m, 8H), 1.37 (qt, J = 6.6, 3.4 Hz, 4H), 1.30 – 1.19 (m, 19H), 0.89 – 0.81 (m, 6H).

^{13}C NMR (100 MHz, $CDCl_3$) δ 173.43, 172.96, 65.40, 65.22, 62.73, 46.37, 34.24, 32.63, 31.74, 29.15, 28.99, 28.57, 25.76, 25.44, 24.98, 22.68, 17.95, 14.14.

HRMS (ESI-TOF) m/z: $[M+H]^+$ Calculated for $C_{27}H_{50}O_7$: 487.3629; found 487.3633.

Synthesis of **Lipid alcohol 8**

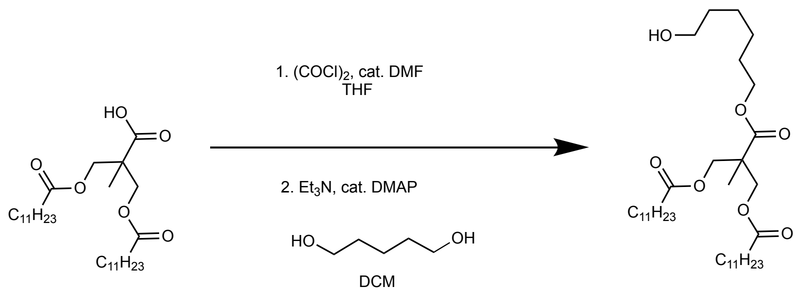

Lipidic acid 6 Lipidic alcohol 8

Lipidic alcohol 8 was formed from **lipidic acid 6** following a protocol similar to that given for **lipidic alcohol 7** formation. **Lipidic acid 6** (1.50g, 3.01 mmol, 1 equiv), oxalyl chloride (284ul, 3.31 mmol, 1.1 equiv), Et_3N (1.25 ml, 9.02 mmol, 1 equiv), 1,6-hexanediol (1.07 g, 9.02 mmol, 3 equiv), and DMAP (147 mg, 1.2 mmol, 0.4 equiv) were subjected to the reaction conditions detailed above, and the amount of solvents (THF, DCM) used were scaled correspondingly. The residue was purified by silica gel flash column chromatography with 25% EtOAc/Hexane as eluent to afford **Lipidic alcohol 8** as a colorless oil (1.24 g, 2.08 mmol 69% yield). Compound purity was established by TLC (one spot) analysis.

^{1}H NMR (400 MHz, $CDCl_3$) δ 4.26 – 4.17 (m, 4H), 4.12 (t, J = 6.6 Hz, 2H), 3.65 (t, J = 6.5 Hz, 2H), 2.29 (t, J = 7.5 Hz, 4H), 1.58 (d, J = 7.5 Hz, 10H), 1.41 – 1.37 (m, 2H), 1.26 (q, J = 6.8 Hz, 35H), 0.88 (t, J = 6.8 Hz, 6H).

^{13}C NMR (100 MHz, $CDCl_3$) δ 173.45, 172.97, 65.41, 65.23, 62.77, 46.39, 34.27, 32.65, 32.02, 29.72, 29.58, 29.45, 29.38, 29.24, 28.59, 25.78, 25.46, 25.01, 22.79, 17.97, 14.22.

HRMS (ESI-TOF) m/z: $[M+H]^+$ Calculated for $C_{35}H_{66}O_7$: 599.4881; found 599.4883.

Synthesis of **Lipidic acid 7**

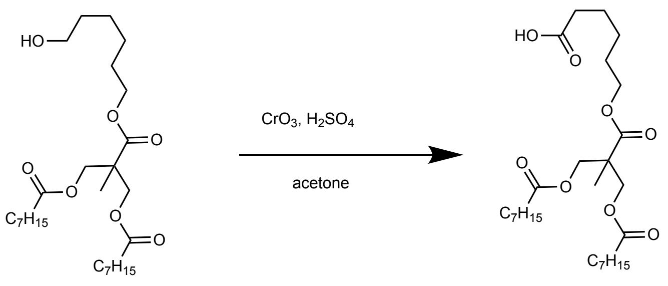

Lipidic alcohol 7 Lipidic acid 7

Lipidic acid 7 was formed from **lipidic alcohol 7** following a protocol similar to that given for **lipidic acid 3** formation (described above). **Lipidic alcohol 7** (800 mg, 1.64 mmol, 1 equiv), CrO_3 (411 mg, 4.11 mmol, 2.5 equiv), and H_2SO_4 (596 mg, 6.08 mmol, 3.7 equiv), and 12 ml acetone were subjected to the reaction condition detailed above. Purification was accomplished by silica gel flash column chromatography (15-30% EtOAc/Hexane, 1% AcOH as eluent) affording **Lipidic acid 7** (642 mg, 1.28 mmol, 78% yield) as a slightly yellow oil. Compound purity was established by TLC (one spot) analysis.

^{1}H NMR (400 MHz, $CDCl_3$) δ 4.29 – 4.16 (m, 4H), 4.15 – 4.08 (m, 2H), 2.42 – 2.33 (m, 2H), 2.33 – 2.23 (m, 4H), 1.74 – 1.49 (m, 8H), 1.47 – 1.35 (m, 2H), 1.26 (ddd, J = 14.0, 6.2, 3.1 Hz, 19H), 0.93 – 0.81 (m, 6H).

^{13}C NMR (100 MHz, $CDCl_3$) δ 179.41, 173.47, 172.93, 65.39, 64.93, 46.39, 34.24, 33.87, 31.74, 29.15, 29.00, 28.26, 25.37, 24.98, 24.27, 22.68, 17.93, 14.14.

HRMS (ESI-TOF) m/z: $[M+H]^+$ Calculated for $C_{27}H_{48}O_8$: 501.3422; found 501.3422.

Synthesis of **Lipidic acid 8**

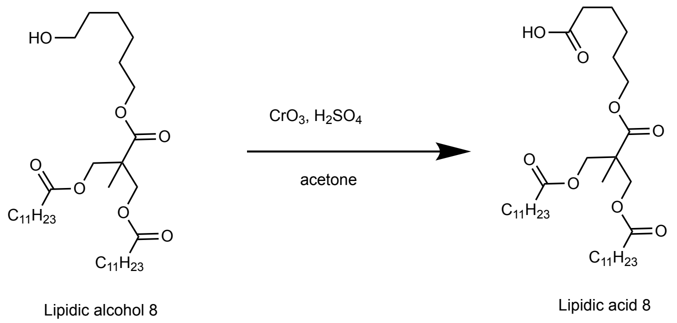

Lipidic acid 8 was formed from via **lipidic alcohol 8** following a protocol similar to that given for **lipidic acid 3** formation (described above). **Lipidic alcohol 8** (500mg, 0.835 mmol, 1 equiv), CrO_3 (209mg, 2.06 mmol, 2.5 equiv), H_2SO_4 (303 mg, 3.09 mmol, 3.7 equiv), and 6 ml acetone were subjected to the reaction condition detailed above. The residue was purified with silica gel column chromatography with 25% EtOAc/Hexane, 1% AcOH as eluent to afford **lipidic acid 8** as a colorless oil (410 mg, 0.68 mmol, 81% yield). Compound purity was established by TLC (one spot) analysis.

^{1}H NMR (400 MHz, $CDCl_3$) δ 4.29 – 4.17 (m, 4H), 4.15 – 4.04 (m, 2H), 2.40 – 2.32 (m, 2H), 2.32 – 2.22 (m, 4H), 1.73 – 1.56 (m, 8H), 1.47 – 1.35 (m, 2H), 1.25 (q, J = 6.8 Hz, 35H), 0.87 (t, J = 6.8 Hz, 6H).

^{13}C NMR (100 MHz, $CDCl_3$) δ 179.22, 173.55, 172.95, 65.42, 64.95, 46.43, 34.29, 33.79, 32.05, 29.75, 29.61, 29.48, 29.40, 29.26, 28.27, 25.61, 25.38, 25.04, 24.34, 22.82, 17.99, 14.25.

HRMS (ESI-TOF) m/z: $[M+H]^+$ Calculated for $C_{35}H_{64}O_8$: 635.4493; found 635.4493.

A2. Synthesis of bAC monomers

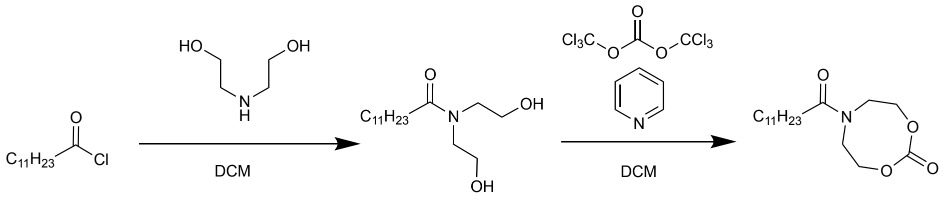

Lauroyl chloride bAC-1

Synthesis of **bAC-1**

Lauroyl chloride (315 mg, 1.44 mmol, 1 equiv) was dissolved in 1.5 ml dry DCM. Diethanolamine (908 mg, 8.64 mmol, 6 equiv) was added to 9.0 ml DCM with vigorous stirring. The acid chloride solution was added dropwise over 20 min at room temperature. After 5 h, the reaction mixture was washed with 10 ml aqueous 1 N HCl and brine, and dried over $MgSO_4$. The solvent was removed *in vacuo*, and the acylated diethanolamine (white solid) product was placed under high vacuum for 16 h. Acylated diethanolamine was then dissolved in dry DCM (36.0 ml), and pyridine (890 µL, 8.64 mmol, 6 equiv) was added. At -20 °C, triphosgene (170 mg, 0.576 mmol, 0.4 equiv) was dissolved in dry DCM (10.0 ml) and added slowly dropwise over 0.5 h. The reaction mixture was allowed to warm to room temperature (25 °C) and stirred for an additional 1 h. An NMR was taken to ensure complete consumption of starting materials. The organic layers were then washed with 25.0 ml aqueous 1 N NH_4Cl solution, brine and dried over $MgSO_4$. The solvent was removed *in vacuo* to provide the crude product as a slightly yellow oil. Purification was accomplished by silica gel flash column chromatography (40-60% EtOAc/Hexane, 1% AcOH as eluent) affording **bAC-1** (220 mg, 0.71 mmol, 51% yield) as a white solid. Compound purity was established by TLC (one spot) analysis.

^{1}H NMR (400 MHz, $CDCl_3$) δ 4.41 (dt, *J* = 10.8, 5.1 Hz, 4H), 3.66 (dt, *J* = 7.2, 4.9 Hz, 4H), 2.35 – 2.27 (m, 2H), 1.63 (q, *J* = 7.3 Hz, 2H), 1.35 – 1.22 (m, 16H), 0.88 (t, *J* = 6.7 Hz, 3H).

^{13}C NMR (100 MHz, $CDCl_3$) δ 173.76, 155.53, 70.18, 67.44, 49.86, 48.61, 32.01, 29.73, 29.71, 29.60, 29.54, 29.46, 29.43, 29.23, 24.94, 22.79, 14.22.

HRMS calculated for $C_{17}H_{31}NO_4$ [M+H]+: 314.44; found 314.52

HRMS (ESI-TOF) m/z: $[M+H]^+$ Calculated for $C_{17}H_{31}NO_4$: 314.4425; found 314.4462.

Synthesis of **bAC-2**

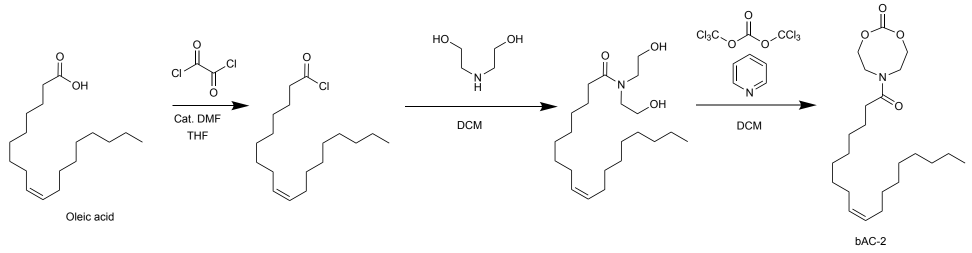

Oleic acid (500 mg, 1.77 mmol, 1 equiv) was dissolved in 5.9 ml dry THF and 10 drops of DMF were added. The solution was chilled on an ice bath for 5 min and oxalyl chloride (167 ul, 1.95 mmol, 1.1 equiv) was added dropwise with a syringe over 5 min. The reaction was allowed to warm up to room temperature and stirred for 1 h after addition, and the solvent was evaporated *in vacuo* to yield the crude acid chloride. The crude intermediate was dried under high vacuum for additional 2 hour, and then redissolved in dry DCM (2.0 ml). Diethanolamine (1.12 g, 10.62 mmol, 6 equiv) was resuspended in 11.0 ml DCM with fast stirring. The acid chloride solution was then added dropwise over 20 min at room temperature. After 5 h, the reaction mixture was washed with 15 ml aqueous 1N HCl and brine, and dried with $MgSO_4$. The organic layer was evaporated *in vacuo*, and the acylated diethanolamine (slightly yellow oil) product placed under high vacuum for 16 h. Acylated diethanolamine was then dissolved in dry DCM (45.0 ml), and pyridine (1.09 ml, 10.62 mmol, 6 equiv) was added. Solution was cooled to -20°C (brine/dry ice bath). Triphosgene (210 mg, 0.708 mmol, 0.4 equiv) was dissolved in dry DCM (12.0 ml) and added slowly dropwise over 0.5 h. The reaction mixture was allowed to warm to room temperature (25 °C) and stirred for an additional 1 h. An NMR was taken to ensure complete consumption of starting materials. The organic layers were then washed with 40 ml aqueous 1M NH_4Cl and dried with brine and $MgSO_4$ to yield the crude product. The residue was purified with silica gel flash chromatography with 40% EtOAc/Hexane as eluent to afford **bAC-2** as a slowly solidifying colorless oil (352 mg, 0.90 mmol, 51% yield). Compound purity was established by TLC (one spot) analysis.

^{1}H NMR (400 MHz, $CDCl_3$) δ 5.37 (dtt, J = 11.1, 7.5, 3.9 Hz, 2H), 4.44 (dq, J = 10.9, 6.6 Hz, 4H), 3.68 (dt, J = 7.4, 4.9 Hz, 4H), 2.34 (q, J = 8.7 Hz, 2H), 2.02 (dp, J = 12.9, 7.5 Hz, 4H), 1.69 (d, J = 7.4 Hz, 2H), 1.48 – 1.25 (m, 20H), 0.90 (dd, J = 9.2, 4.1 Hz, 3H).

^{13}C NMR (100 MHz, $CDCl_3$) δ 173.59, 155.40, 130.00, 129.79, 77.24, 70.13, 67.37, 49.82, 48.58, 33.33, 32.63, 31.92, 29.79, 29.74, 29.54, 29.37, 29.34, 29.17, 27.24, 27.20, 24.84, 22.70, 14.14.

HRMS (ESI-TOF) m/z: $[M+H]^+$ Calculated for $C_{23}H_{41}NO_4$: 396.3108; found 396.3100.

Synthesis of **bAC-3**

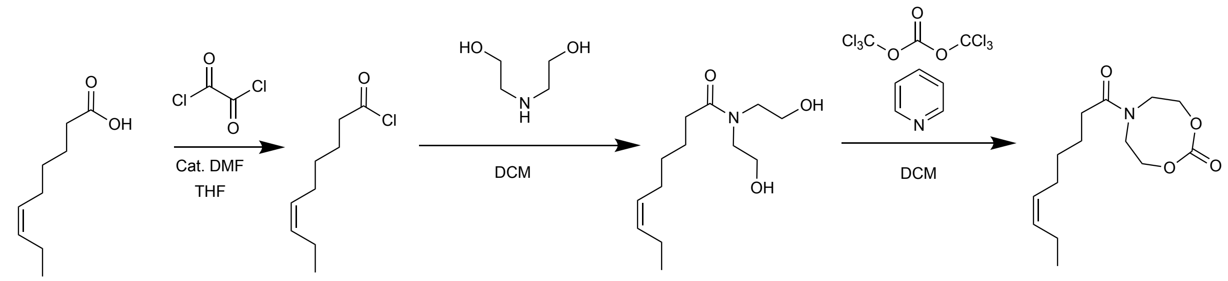

Lipidic acid 3 bAC-3

bAC-3 was formed from **lipidic acid 3** following a protocol similar to that given for **bAC-2** formation (described above). **Lipidic acid 3** (570mg, 3.65 mmol, 1 equiv), oxalyl chloride (343 ul, 4.01 mmol, 1.1 equiv), diethanolamine (2.31 g, 21.9 mmol, 6 equiv), triphosgene (433 mg, 1.46 mmol, 0.4 equiv), and pyridine (2.26 ml, 21.9 mmol, 6 equiv) were subjected to the reaction conditions detailed above. The amounts of solvents used in the reactions (THF, DCM) were scaled with the amount of substrate accordingly to maintain the same molar concentration. The residue after workup was purified by silica gel column chromatography with 40% EtOAc/Hexane as eluent to afford **bAC-3** as a slightly yellow oil (499 mg, 1.86 mmol, 51% yield). Compound purity was established by TLC (one spot) analysis.

^{1}H NMR (400 MHz, $CDCl_3$) δ 5.32 (ddt, J = 17.9, 11.2, 5.7 Hz, 2H), 4.40 (dt, J = 11.1, 5.0 Hz, 4H), 3.64 (dt, J = 9.9, 5.2 Hz, 4H), 2.35 – 2.26 (m, 2H), 2.03 (h, J = 7.1 Hz, 4H), 1.65 (p, J = 7.6 Hz, 2H), 1.38 (p, J = 7.5 Hz, 2H), 0.94 (t, J = 7.5 Hz, 3H).

^{13}C NMR (100 MHz, $CDCl_3$) δ 173.58, 155.51, 132.15, 128.72, 70.20, 67.42, 49.89, 48.67, 33.30, 29.50, 26.98, 24.56, 20.65, 14.48.

HRMS (ESI-TOF) m/z: $[M+H]^+$ Calculated for $C_{14}H_{23}NO_4$: 270.1700; found 270.1699.

Synthesis of **bAC-4**

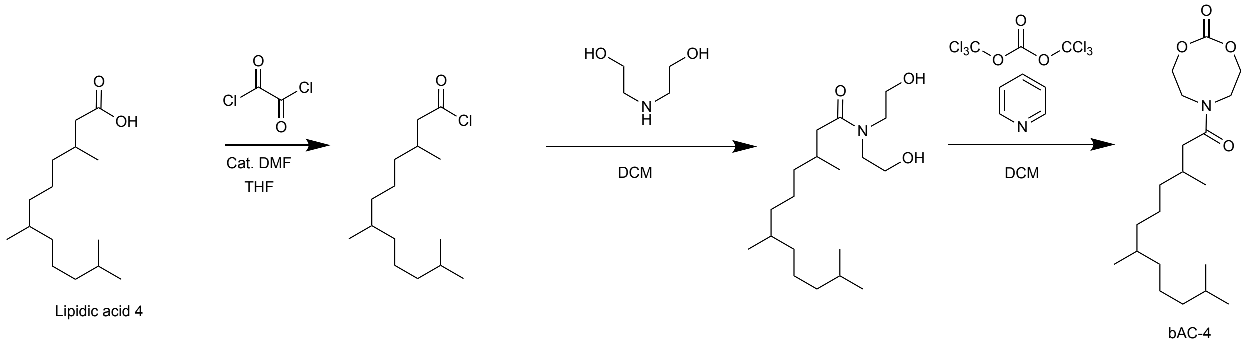

bAC-4 was formed from **lipidic acid 4** following a protocol similar to that given for **bAC-2** formation (described above). **Lipidic acid 4** (724 mg, 2.99 mmol, 1 equiv), oxalyl chloride (307 ul, 3.58 mmol, 1.1 equiv), diethanolamine (1.89 g, 17.94 mmol, 6 equiv), triphosgene (356 mg, 1.2 mmol, 0.4 equiv), and pyridine (1.85 ml, 17.94 mmol, 6 equiv) were subjected to the reaction condition detailed above. The amounts of solvents used in the reactions (THF, DCM) were scaled with the amount of substrate accordingly to maintain the same molar concentration. The residue after workup was purified by silica gel column chromatography with 50% EtOAc/Hexane as eluent to afford **bAC-4** as a yellow oil (499 mg, 1.86 mmol, 51% yield). Compound purity was established by TLC (one spot) analysis.

^{1}H NMR (400 MHz, $CDCl_3$) δ 4.41 (dt, J = 13.1, 5.1 Hz, 4H), 3.76 – 3.54 (m, 4H), 2.36 – 2.08 (m, 2H), 1.37 – 1.06 (m, 12H), 0.94 (d, J = 6.5 Hz, 3H), 0.85 (dd, J = 9.1, 6.6 Hz, 12H).

^{13}C NMR (100 MHz, $CDCl_3$) δ 173.20, 155.47, 70.27, 67.61, 49.99, 48.70, 40.77, 40.70, 39.45, 37.46, 37.32, 32.88, 30.11, 28.07, 24.89, 24.59, 22.83, 22.73, 19.99, 19.83.

HRMS (ESI-TOF) m/z: $[M+H]^+$ Calculated for $C_{20}H_{37}NO_4$: 356.2795; found 356.2788.

Synthesis of **bAC-5**

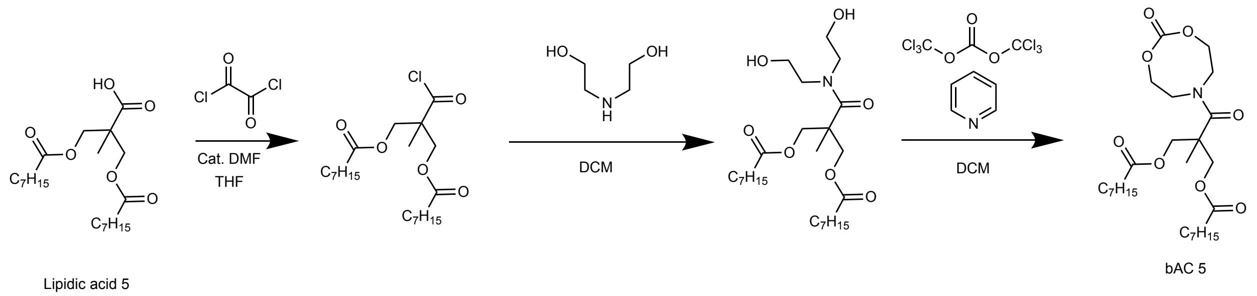

bAC-5 was formed from **lipidic acid 5** following a protocol similar to that given for bAC-2 formation (described above). **Lipidic acid 5** (600 mg, 1.36 mmol, 1 equiv), oxalyl chloride (140 ul, 1.63 mmol, 1.1 equiv), diethanolamine (855 mg, 8.13 mmol, 6 equiv), triphosgene (160 mg, 0.54 mmol, 0.4 equiv), and pyridine (856 ul, 8.13 mmol, 6 equiv) were subjected to the reaction condition detailed above. The amounts of solvents used in the reactions (THF, DCM) were scaled with the amount of substrate accordingly to maintain the same molar concentration. The residue after workup was purified by silica gel flash chromatography with 40% EtOAc/Hexane as eluent to afford **bAC-5** as a slightly yellow oil (319 mg, 0.64 mmol, 47% yield). Compound purity was established by TLC (one spot) analysis.

^{1}H NMR (400 MHz, $CDCl_3$) δ 4.42 (t, *J* = 4.9 Hz, 4H), 4.37 (d, *J* = 11.5 Hz, 2H), 4.24 (d, *J* = 11.5 Hz, 2H), 3.72 (t, *J* 5.0 Hz, 4H), 2.31 (t, *J* = 7.6 Hz, 4H), 1.59 (p, *J* = 7.2 Hz, 4H), 1.35 (s, 3H), 1.27 (ddd, *J* = 8.0, 5.6, 3.1 Hz, 16H), 0.91 – 0.83 (m, 6H).

^{13}C NMR (100 MHz, $CDCl_3$) δ 173.67, 172.61, 155.34, 68.74, 65.71, 50.33, 46.98, 34.35, 31.83, 29.25, 29.08, 25.05, 22.77, 17.80, 14.24.

HRMS (ESI-TOF) m/z: $[M+H]^+$ Calculated for $C_{26}H_{45}NO_8$: 500.3233; found 500.3235.

Synthesis of **bAC-6**

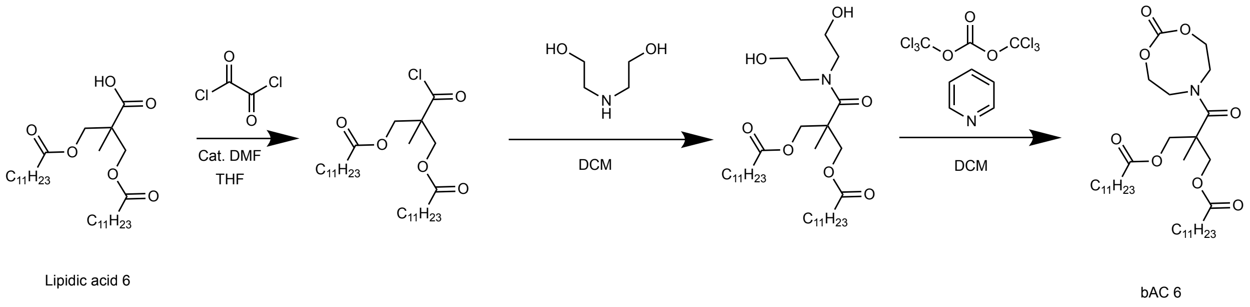

bAC-6 was formed from **lipidic acid 6** following a protocol similar to that given for **bAC-2** formation (described above). **Lipidic acid 6** (750 mg, 1.50 mmol, 1 equiv), oxalyl chloride (155 ul, 1.80 mmol, 1.1 equiv), diethanolamine (945 mg, 9.02 mmol, 6 equiv), triphosgene (178 mg, 0.60 mmol, 0.4 equiv), and pyridine (950 ul, 9.02 mmol, 6 equiv) were subjected to the reaction condition detailed above. The amounts of solvents used in the reactions (THF, DCM) were scaled with the amount of substrate accordingly to maintain the same molar concentration. The residue after workup was purified by silica gel flash column chromatography with 35% EtOAc/Hexane as eluent to afford **bAC-6** as a slowly solidifying colorless oil (388 mg, 0.63 mmol, 42% yield). Compound purity was established by TLC (one spot) analysis.

^{1}H NMR (400 MHz, $CDCl_3$) δ 4.41 (t, J = 4.9 Hz, 4H), 4.36 (d, J = 11.5 Hz, 2H), 4.23 (d, J = 11.5 Hz, 2H), 3.71 (t, J = 4.8 Hz, 4H), 2.30 (t, J = 7.6 Hz, 4H), 1.59 (d, J = 7.2 Hz, 4H), 1.34 (s, 3H), 1.25 (d, J = 5.5 Hz, 32H), 0.86 (t, J = 6.7 Hz, 6H).

^{13}C NMR (100 MHz, $CDCl_3$) δ 173.59, 172.55, 155.28, 68.67, 65.64, 50.25, 46.90, 34.29, 32.02, 29.71, 29.58, 29.44, 29.37, 29.24, 24.99, 22.79, 17.72, 14.22.

HRMS (ESI-TOF) m/z: $[M+H]^+$ Calculated for $C_{34}H_{61}NO_8$: 612.4470; found 612.4456.

Synthesis of **bAC-7**

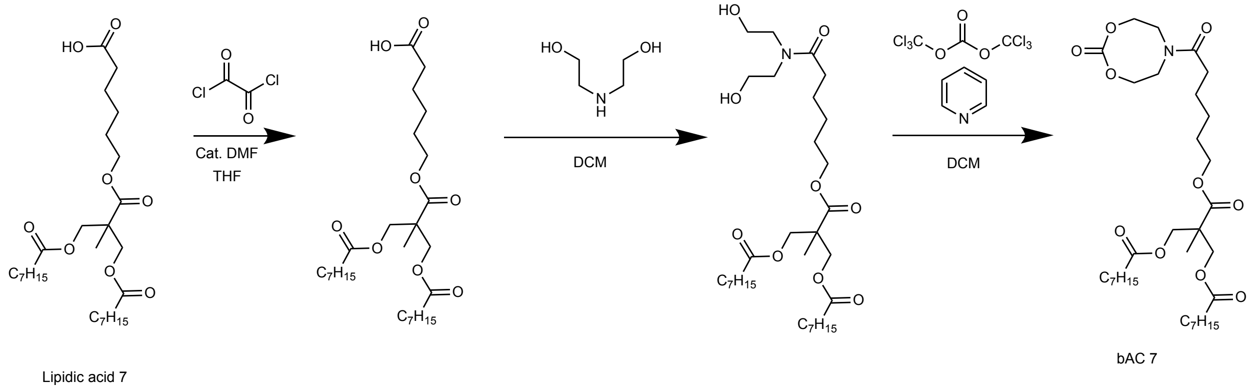

bAC-7 was formed from **lipidic acid 7** following a protocol similar to that given for **bAC-2** formation (described above). **Lipidic acid 7** (700 mg, 1.40 mmol, 1 equiv), oxalyl chloride (132 ul, 1.54 mmol, 1.1 equiv), diethanolamine (882 mg, 8.37 mmol, 6 equiv), triphosgene (269 mg, 0.91 mmol, 0.4 equiv), and pyridine (674 ul, 8.37 mmol, 6 equiv) were subjected to the reaction conditions detailed above. The amounts of solvents used in the reactions (THF, DCM) were scaled with the amount of substrate accordingly to maintain the same molar concentration. The residue after workup was purified by silica gel flash column chromatography with 65% EtOAc/Hexane as eluent to afford **bAC-7** as a slightly yellow oil (316 mg, 0.59 mmol, 42% yield). Compound purity was established by TLC (one spot) analysis.

^{1}H NMR (400 MHz, $CDCl_3$) δ 4.50 – 4.32 (m, 4H), 4.27 – 4.15 (m, 4H), 4.15 – 4.04 (m, 2H), 3.65 (q, J = 4.9 Hz, 4H), 2.30 (dt, J = 15.4, 7.4 Hz, 6H), 1.73 – 1.52 (m, 8H), 1.44 – 1.34 (m, 2H), 1.33 – 1.21 (m, 19H), 0.87 (t, J = 6.7 Hz, 6H).

^{13}C NMR (100 MHz, $CDCl_3$) δ 173.45, 173.23, 172.94, 155.48, 70.13, 67.22, 65.38, 65.09, 49.80, 48.68, 46.40, 34.27, 33.10, 31.77, 29.18, 29.03, 28.47, 25.64, 25.02, 24.39, 22.71, 17.98, 14.18.

HRMS (ESI-TOF) m/z: $[M+H]^+$ Calculated for $C_{32}H_{55}NO_{10}$: 614.3899; found 614.3899.

Synthesis of **bAC-8**

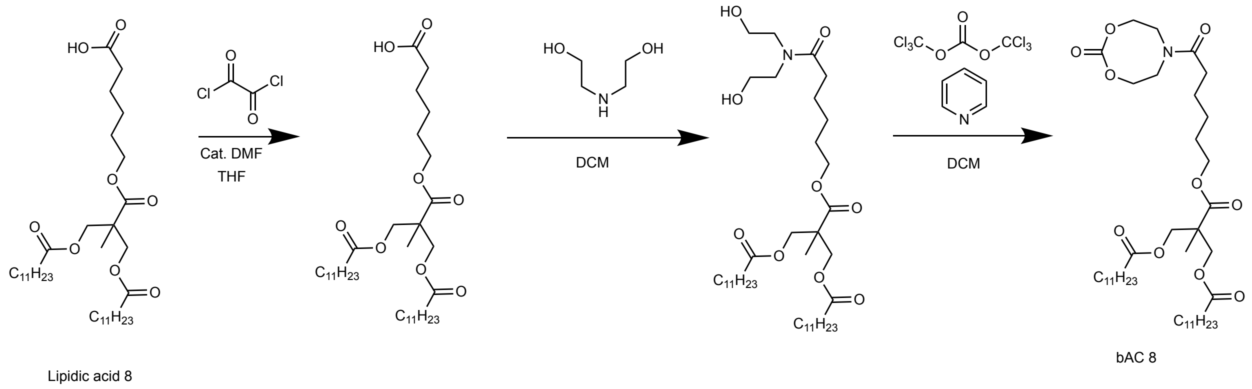

bAC-8 was formed from **lipidic acid 8** following a protocol similar to that given for **bAC-2** formation (described above). **Lipidic acid 8** (580 mg, 0.95 mmol, 1 equiv), oxalyl chloride (90 ul, 1.04 mmol, 1.1 equiv), diethanolamine (597 mg, 5.68 mmol, 6 equiv), triphosgene (112 mg, 0.38 mmol, 0.4 equiv), and pyridine (489 ul, 5.68 mmol, 6 equiv) were subjected to the reaction condition detailed above. The amounts of solvents used in the reactions (THF, DCM) were scaled with the amount of substrate accordingly to maintain the same molar concentration. The residue after workup was purified by silica gel flash column chromatography with 65% EtOAc/Hexane as eluent to afford **bAC-8** as a slightly yellow oil (299 mg, 0.41 mmol, 43% yield). Compound purity was established by TLC (one spot) analysis.

^{1}H NMR (400 MHz, $CDCl_3$) δ 4.38 (q, J = 5.1 Hz, 4H), 4.24 – 4.13 (m, 4H), 4.09 (t, J = 6.6 Hz, 2H), 3.63 (q, J = 4.7 Hz, 4H), 2.28 (dt, J = 17.3, 7.5 Hz, 6H), 1.70 – 1.49 (m, 8H), 1.41 – 1.31 (m, 2H), 1.23 (q, J = 6.3 Hz, 35H), 0.88 – 0.80 (m, 6H).

^{13}C NMR (100 MHz, $CDCl_3$) δ 173.35, 173.16, 172.86, 155.42, 70.03, 67.14, 65.30, 65.02, 49.71, 48.58, 46.34, 34.20, 33.02, 31.96, 29.66, 29.53, 29.39, 29.32, 29.18, 28.41, 25.57, 24.97, 24.33,

HRMS (ESI-TOF) m/z: $[M+H]^+$ Calculated for $C_{40}H_{71}NO_{10}$: 726.5150; found 726.5151.

B. Synthesis of MTC monomers (MTC-1, -4, -7, -8)

Synthesis of **MTC-1**

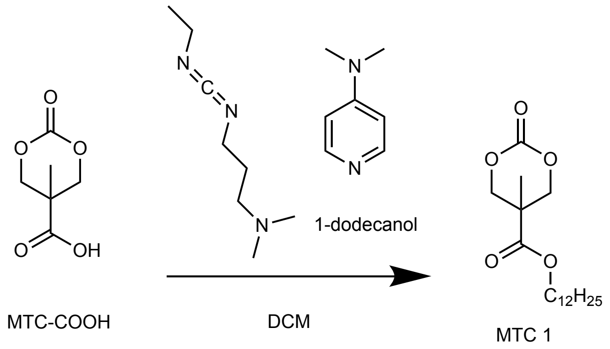

1-dodecanol (150 mg, 0.8 mmol, 1 equiv) and MTC-COOH (155 mg, 0.96 mmol, 1.2 equiv) were dissolved in DCM (1.5 ml) at room temperature (25°C). EDCI (187 mg, 1.2 mmol, 1.5 equiv) and DMAP (40 mg, 0.32 mmol, 0.4 equiv) were added and allowed to react for 16 h. The organic layers were washed with 2 ml aqueous 1N HCl and with brine, then dried with $MgSO_4$. The solvent was evaporated *in vacuo* to get the crude product. The residue was purified by silica gel flash column chromatography with 40% EtOAc/Hexane as eluent to afford **MTC-1** as a colorless oil (142 mg, 0.43 mmol, 54% yield). Compound purity was established by TLC (one spot) analysis. Additional spetra information could be found in the previous literature[1].

^{1}H NMR (400 MHz, $CDCl_3$) δ 4.76 – 4.59 (d, 2H), 4.27 – 4.15 (m, 4H), 1.77 – 1.58 (m, 2H), 1.46 – 1.17 (m, 21H), 0.87 (t, *J* = 6.7 Hz, 3H).

13C NMR (101 MHz, $CDCl_3$) δ 171.57, 147.94, 73.48, 66.88, 40.61, 32.36, 30.07 (broad), 30.00, 29.91, 29.79, 29.60, 28.85, 26.18, 23.14, 18.11, 14.57.

Synthesis of **MTC-4**

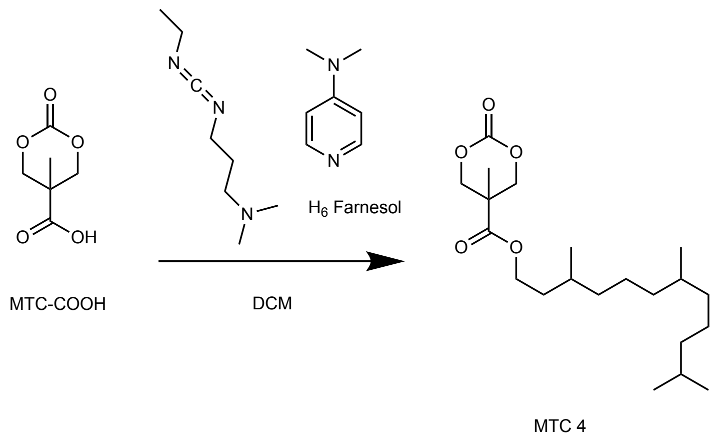

H6-Farnesol (200 mg, 0.88 mmol, 1 equiv) and MTC-COOH (168 mg, 1.05 mmol, 1.2 equiv) were dissolved in DCM (1.5 ml) at room temperature (25°C). EDCI (204 mg, 1.3 mmol, 1.5 equiv) and DMAP (43 mg, 0.35 mmol, 0.4 equiv) were added and allowed to react for 16 h. The organic layers were washed with 2 ml aqueous 1N HCl and with brine, then dried with $MgSO_4$. The solvent was evaporated *in vacuo* to get the crude product. The residue was purified by silica gel flash column chromatography with 50% EtOAc/Hexane as eluent to afford **MTC-4** as a colorless oil (198 mg, 0.54 mmol, 61% yield). Compound purity was established by TLC (one spot) analysis.

^{1}H NMR (400 MHz, $CDCl_3$) δ 4.72 – 4.61 (m, 2H), 4.27 – 4.13 (m, 4H), 1.68 (dddt, *J* = 12.2, 7.1, 4.8, 2.0 Hz, 1H), 1.58 – 1.40 (m, 4H), 1.31 – 0.98 (m, 15H), 0.94 – 0.81 (m, 12H).

^{13}C NMR (101 MHz, $CDCl_3$) δ 171.55, 147.91, 73.45, 65.38, 40.58, 39.79, 37.80, 37.72, 37.62, 35.77, 35.69, 33.19, 30.31, 28.41, 25.23, 24.74, 23.16, 23.06, 20.15, 20.08, 19.92, 18.08.

Synthesis of **MTC-7**

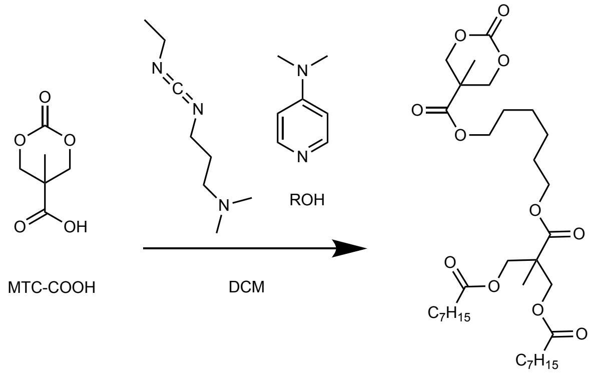

Lipidic alcohol 7 (250 mg, 0.42 mmol, 1 equiv) and MTC-COOH (1.2 equiv) were dissolved in DCM (2 ml) at room temperature (25°C). EDCI (97.7 mg, 0.63 mmol, 1.5 equiv) and DMAP (20.8 mg, 0.17 mmol, 0.4 equiv) were added and allowed to react for 16 h. The organic layers were washed with 2 ml aqueous 1N HCl and with brine, then dried with $MgSO_4$. The solvent was evaporated *in vacuo* to get the crude product. The residue was purified by silica gel flash column chromatography with 50% EtOAc/Hexane as eluent to afford **MTC-7** as a colorless oil (161 mg, 0.26 mmol, 61% yield). Compound purity was established by TLC (one spot) analysis.

^{1}H NMR (400 MHz, $CDCl_3$) δ 4.68 (dd, J = 11.1, 1.5 Hz, 2H), 4.33 – 4.14 (m, 8H), 4.11 (t, J = 6.6 Hz, 2H), 2.29 (t, J = 7.5 Hz, 4H), 1.73 – 1.53 (m, 8H), 1.41 – 1.34 (m, 4H), 1.34 – 1.18 (m, 22H), 0.91 – 0.84 (m, 6H).

^{13}C NMR (100 MHz, $CDCl_3$) δ 172.43, 171.97, 170.23, 72.15, 65.22, 64.37, 64.07, 45.41, 39.33, 33.27, 30.78, 28.19, 28.03, 27.49, 27.46, 24.57, 24.49, 24.02, 21.72, 17.00, 16.75, 13.19.

HRMS (ESI-TOF) m/z: $[M+H]^+$ Calculated for $C_{33}H_{56}NO_{11}$: 629.3895; found 629.3894.

Synthesis of **MTC-8**

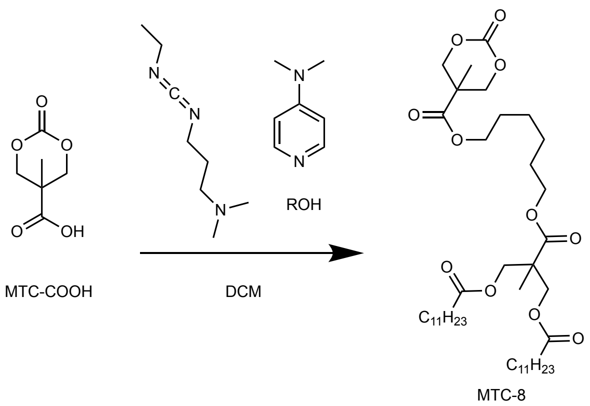

MTC-8 was formed from **lipidic alcohol 8** following a protocol similar to that given for **MTC-7** formation (describe above). **Lipidic alcohol 8** (316 mg, 0.65 mmol, 1 equiv), MTC-COOH (124.8 mg, 0.78 mmol, 1.2 equiv), EDCI (151 mg, 0.97 mmol, 1.5 equiv), DMAP (32 mg, 0.26 mmol, 0.4 equiv), and 1.5 ml DCM were subjected to the reaction conditions detailed above. The residue after workup was purified by silica gel flash column chromatography with 40% EtOAc/Hexane as eluent to afford **MTC-8** as a slightly yellow oil (269 mg, 0.36 mmol, 56% yield). Compound purity was established by TLC (one spot) analysis.

^{1}H NMR (400 MHz, $CDCl_3$) δ 4.69 (dd, *J* = 11.2, 1.5 Hz, 2H), 4.27 – 4.16 (m, 8H), 4.11 (t, *J* = 6.6 Hz, 2H), 2.29 (t, *J* = 7.5 Hz, 4H), 1.71 – 1.55 (m, 8H), 1.42 – 1.34 (m, 4H), 1.34 – 1.21 (m, 38H), 0.88 (t, *J* = 6.7 Hz, 6H).

^{13}C NMR (100 MHz, $CDCl_3$) δ 173.52, 173.06, 171.32, 73.24, 66.30, 65.46, 65.16, 46.51, 40.42, 34.37, 32.13, 29.83, 29.69, 29.55, 29.49, 29.35, 28.59, 28.56, 25.66, 25.59, 25.12, 22.90, 18.10, 17.84, 14.34.

HRMS (ESI-TOF) m/z: $[M+H]^+$ Calculated for $C_{41}H_{72}NO_{11}$: 741.5147; found 741.5150.

Supplementary References

1. McKinlay, C. J., Benner, N. L., Haabeth, O. A., Waymouth, R. M. & Wender, P. A. Enhanced mRNA delivery into lymphocytes enabled by lipid-varied libraries of charge-altering releasable transporters. *Proc. Natl. Acad. Sci. U.S.A.* **115,** E5859–E5866 (2018).
2. Muñoz, L., Guerrero, A., Synthesis of a New Deuterium-Labeled Phytol as a Tool for Biosynthetic Studies. *Synthesis* 44, 862-864 (2012).
