# Supplementary material for: Charge-altering releasable transporters enhance mRNA delivery in vitro and exhibit in vivo tropism
Source: Nat Commun. 2023 Nov 1;14:6983. doi: 10.1038/s41467-023-42672-x (PMC10620205; doi:10.1038/s41467-023-42672-x)
Supplement: Supplementary file 3 — Reporting Summary [file 41467_2023_42672_MOESM3_ESM.pdf]

nature portfolio

Corresponding author(s): Paul A. Wender

Last updated by author(s): Sep 25, 2023

Reporting Summary

Nature Portfolio wishes to improve the reproducibility of the work that we publish. This form provides structure for consistency and transparency in reporting. For further information on Nature Portfolio policies, see our Editorial Policies and the Editorial Policy Checklist.

Statistics

For all statistical analyses, confirm that the following items are present in the figure legend, table legend, main text, or Methods section.

n/a	Confirmed	
☐	☒	The exact sample size (*n*) for each experimental group/condition, given as a discrete number and unit of measurement
☐	☒	A statement on whether measurements were taken from distinct samples or whether the same sample was measured repeatedly
☐	☒	The statistical test(s) used AND whether they are one- or two-sided<br>*Only common tests should be described solely by name; describe more complex techniques in the Methods section.*
☒	☐	A description of all covariates tested
☒	☐	A description of any assumptions or corrections, such as tests of normality and adjustment for multiple comparisons
☐	☒	A full description of the statistical parameters including central tendency (e.g. means) or other basic estimates (e.g. regression coefficient) AND variation (e.g. standard deviation) or associated estimates of uncertainty (e.g. confidence intervals)
☐	☒	For null hypothesis testing, the test statistic (e.g. *F*, *t*, *r*) with confidence intervals, effect sizes, degrees of freedom and *P* value noted<br>*Give P values as exact values whenever suitable.*
☒	☐	For Bayesian analysis, information on the choice of priors and Markov chain Monte Carlo settings
☒	☐	For hierarchical and complex designs, identification of the appropriate level for tests and full reporting of outcomes
☒	☐	Estimates of effect sizes (e.g. Cohen's *d*, Pearson's *r*), indicating how they were calculated

Our web collection on statistics for biologists contains articles on many of the points above.

Software and code

Policy information about availability of computer code

Data collection	Flow cytometry data was acquired with Attune NxT and Cytek Aurora System. Animal imaging was collected with Ami HTX- Spectral Instruments Imaging and Aurora Aura Imaging Software.
Data analysis	Flow cytometry data was analyzed with Flowjo 10.9.0. Data analysis was performed with GraphPad Prism 9.2.0

For manuscripts utilizing custom algorithms or software that are central to the research but not yet described in published literature, software must be made available to editors and reviewers. We strongly encourage code deposition in a community repository (e.g. GitHub). See the Nature Portfolio guidelines for submitting code & software for further information.

Data

Policy information about availability of data

All manuscripts must include a data availability statement. This statement should provide the following information, where applicable:

- Accession codes, unique identifiers, or web links for publicly available datasets
- A description of any restrictions on data availability
- For clinical datasets or third party data, please ensure that the statement adheres to our policy

All the data generated in this study are provided in the Source Data file.

Research involving human participants, their data, or biological material

Policy information about studies with human participants or human data. See also policy information about sex, gender (identity/presentation), and sexual orientation and race, ethnicity and racism.

Reporting on sex and gender	Fresh peripheral blood from healthy human donors, of unknown sex and age, was collected, followed by density centrifugation to obtain bulk peripheral blood mononuclear cells (PBMCs) cells which were then frozen and stored in liquid nitrogen until later use and isolation of T cells.
Reporting on race, ethnicity, or other socially relevant groupings	NA
Population characteristics	NA
Recruitment	Fresh PBMCs were obtained from de-identified healthy donors at the Stanford Blood Center.
Ethics oversight	NA

Note that full information on the approval of the study protocol must also be provided in the manuscript.

Field-specific reporting

Please select the one below that is the best fit for your research. If you are not sure, read the appropriate sections before making your selection.

☒ Life sciences ☐ Behavioural & social sciences ☐ Ecological, evolutionary & environmental sciences

For a reference copy of the document with all sections, see nature.com/documents/nr-reporting-summary-flat.pdf

Life sciences study design

All studies must disclose on these points even when the disclosure is negative.

Sample size	For in vivo experiments: N = 3 or 4 mice for animal experiments and N=4 for in vitro experiments. Those sample sizes were chosen to ensure the accuracy of the data and accurate statistics. No statistical method was used to predetermine sample size.
Data exclusions	No data were excluded from the analyses.
Replication	All in vitro and in vivo assays were confirmed at least two times independently and successfully.
Randomization	Mice were randomly selected, and no algorithm was used.
Blinding	The Investigators were not blinded to allocation during experiments and outcome assessment. Blinding investigators was not necessary to perform this research.

Reporting for specific materials, systems and methods

We require information from authors about some types of materials, experimental systems and methods used in many studies. Here, indicate whether each material, system or method listed is relevant to your study. If you are not sure if a list item applies to your research, read the appropriate section before selecting a response.

Materials & experimental systems

n/a	Involved in the study	
☐	☒	Antibodies
☐	☒	Eukaryotic cell lines
☒	☐	Palaeontology and archaeology
☐	☒	Animals and other organisms
☒	☐	Clinical data
☒	☐	Dual use research of concern
☒	☐	Plants

Methods

n/a	Involved in the study	
☒	☐	ChIP-seq
☐	☒	Flow cytometry
☒	☐	MRI-based neuroimaging

Antibodies

Antibodies used	The antibody clones used were the following:<br>FITC anti-mouse CD45. Clone: 30-F11. BioLegend. Cat: 103108. Lot: B275661. Dilution: 1:300

PerCP/Cy5.5 anti-mouse CD19. Clone: 1D3/CD19. BioLegend. Cat: 152405. Lot: B289583. Dilution: 1:200
APC/Cy7 anti-mouse CD4. Clone: GK1.5. BioLegend. Cat: 100413. Lot: B251979. Dilution: 1:200
BV650 anti-mouse CD8a. Clone: 53-6.7. BioLegend. Cat: 100742. Lot: B277562. Dilution: 1:200
APC anti-mouse CD11c. Clone: N418. eBioscience. Cat: 17-0114-82. Lot: 2162250. Dilution: 1:400
BV421 anti-mouse F4/80. Clone: BM8. BioLegend. Cat: 123131. Lot: B323607. Dilution: 1:100
BV650 anti-human CD4. Clone: SK3. BD. Cat: 563876. Lot: 2294796. Dilution: 1:300
PerCP-cy5.5 anti-human CD8a. Clone RPA-T8. BD. Cat: 560662. Lot: 1328433. Dilution: 1:200
BV785 anti-human IFNγ. Clone 4S.B3. BioLegend. Cat: 502541. Dilution: 1:200
BV650 anti-human TNFα. Clone MAb11. BioLegend. Cat: 502937. Dilution: 1:800
BV711 anti-human CD107a. Clone H4A3. BioLegend. Cat: 328639. Dilution: 1:800
BV421 anti-human CD3. Clone OKT3. BioLegend. Cat: 317343. Dilution: 1:250
APC anti-human CD8. Clone SK1. BioLegend. Cat: 344721. Dilution: 1:250

Validation

All antibodies are commercially available and were validated by the manufacturers as documented on the website of the company.
FITC anti-mouse CD45. Clone: 30-F11.
https://www.biolegend.com/ja-jp/products/fitc-anti-mouse-cd45-antibody-99?GroupID=BLG1932
PerCP/Cy5.5 anti-mouse CD19
https://www.biolegend.com/en-us/products/percp-cyanine5-5-anti-mouse-cd19-antibody-13640?GroupID=BLG15527
APC/Cy7 anti-mouse CD4
https://www.biolegend.com/fr-ch/cell-health/apc-cyanine7-anti-mouse-cd4-antibody-1964?GroupID=BLG4745
BV650 anti-mouse CD8a
https://www.biolegend.com/fr-ch/products/brilliant-violet-605-anti-mouse-cd8a-antibody-7636?GroupID=BLG2559
APC anti-mouse CD11c
https://www.thermofisher.com/antibody/product/CD11c-Antibody-clone-N418-Monoclonal/17-0114-82
BV421 anti-mouse F4/80
https://www.biolegend.com/de-at/products/brilliant-violet-421-anti-mouse-f4-80-antibody-7199?GroupID=BLG5319
BV650 anti-human CD4
https://www.bdbiosciences.com/en-ca/products/reagents/flow-cytometry-reagents/research-reagents/single-color-antibodies-ruo/bv650-mouse-anti-human-cd4.563876
PerCP-cy5.5 anti-human CD8a
https://www.bdbiosciences.com/en-us/products/reagents/flow-cytometry-reagents/research-reagents/single-color-antibodies-ruo/percp-cy-5-5-mouse-anti-human-cd8.560662
BV785 anti-human IFNγ
https://www.biolegend.com/en-gb/cell-separation/brilliant-violet-785-anti-human-ifn-gamma-antibody-7986?GroupID=BLG10006
BV650 anti-human TNFα
https://www.biolegend.com/en-us/punchout/punchout-products/product-detail/brilliant-violet-650-anti-human-tnf-alpha-antibody-7680
BV711 anti-human CD107a
https://www.biolegend.com/en-us/products/brilliant-violet-711-anti-human-cd107a-lamp-1-antibody-12003?GroupID=GROUP28
BV421 anti-human CD3
https://www.biolegend.com/en-us/clone-search/brilliant-violet-421-anti-human-cd3-antibody-11976
APC anti-human CD8
https://www.biolegend.com/en-ie/cell-health/apc-anti-human-cd8-antibody-6531?GroupID=BLG10167

Eukaryotic cell lines

Policy information about cell lines and Sex and Gender in Research

Cell line source(s)

Jurkat cell line Clone E6-1 was acquired from ATCC (TIB-152). Nalm6-GL cells that constitutively express GFP and firefly luciferase were a generous gifts from Crystal Mackall Lab in Stanford University.

Authentication

None of the cell lines were authenticated in these studies, but low passage number cell lines were utilized.

Mycoplasma contamination

All cell lines tested negative for mycoplasma contamination.

Commonly misidentified lines
(See ICLAC register)

None

Animals and other research organisms

Policy information about studies involving animals; ARRIVE guidelines recommended for reporting animal research, and Sex and Gender in Research

Laboratory animals

C57BL/6J (#000664), BALB/cJ (#000651) and Ai14 (#007908) were purchased from Jackson Laboratory. All mice were 6 to 8 weeks old at the time of the experiments. All mice in this study were maintained under specific pathogen-free conditions, a 12-h light/12-h dark cycle and temperatures of ~18–23 °C with 40–60% humidity.

Wild animals	None
Reporting on sex	The majority of experiments were performed in female mice, however all observations were replicated in male mice at least once.
Field-collected samples	None
Ethics oversight	All mice experiments were performed according to the approved institutional animal care and use committee (IACUC) protocols of Stanford University.

Note that full information on the approval of the study protocol must also be provided in the manuscript.

Flow Cytometry

Plots

Confirm that:

- [x] The axis labels state the marker and fluorochrome used (e.g. CD4-FITC).
- [x] The axis scales are clearly visible. Include numbers along axes only for bottom left plot of group (a 'group' is an analysis of identical markers).
- [x] All plots are contour plots with outliers or pseudocolor plots.
- [x] A numerical value for number of cells or percentage (with statistics) is provided.

Methodology

Sample preparation	All samples were prepared as described in the Methods section. Briefly, cell suspensions were washed with PBS pH 7.4, stained with viability dye for 10min at RT, washed with PBS +2 % FBS and analyzed immediately by flow cytometry.
Instrument	Flow cytometry data was acquired with Attune NxT and Cytek Aurora System.
Software	Flow cytometry data was analyzed with Flowjo 10.9.0.
Cell population abundance	No cell sorting was performed in this study.
Gating strategy	Cells were gated using FSC/SSC to exclude cell debris and doublets. We gated live cells using eFluor 780 and NIR fixable viability dyes. The gating strategy for the Cre recombinase experiments is described in the Supplementary Information.

- [x] Tick this box to confirm that a figure exemplifying the gating strategy is provided in the Supplementary Information.
